# High‐Pressure Electrosynthesis of Succinic and Adipic Acids From CO_2_ and Ethylene in an Undivided Cell

**DOI:** 10.1002/cssc.70940

**Published:** 2026-08-21

**Authors:** Valtteri Oksanen, Markus Luukkonen, Kiia Malinen, Niko Heikkinen, Alexander Reznichenko, Tom Wirtanen

**Affiliations:** ^1^ Chemical and Polymer Synthesis VTT Technical Research Centre of Finland Espoo Finland; ^2^ School of Chemical Engineering Aalto University Espoo Finland

**Keywords:** C1 building blocks, carboxylation, carboxylic acids, electrochemistry, molecular electrochemistry

## Abstract

Electrosynthesis of succinic and adipic acids from ethylene and carbon dioxide is investigated in a high‐pressure undivided cell. In contrast to the previous studies, we demonstrate that the reaction can proceed without sacrificial anodes with improved current efficiencies for succinic and adipic acids. Mechanistic studies performed with cyclic voltammetry, mass spectrometry, and density‐functional theory are used to elucidate both the cathodic and anodic reaction mechanisms. Preliminary technoeconomic analysis of the synthesis is encouraging toward profitable and efficient synthesis of both succinic and adipic acids from ethylene and CO_2_ in the future.

## Graphical abstract

The first sacrificial anodeless electrosynthesis of succinic and adipic acids from CO_2_ and ethylene is reported. Oxidation of DMF, oxalate, and urea at carbon‐based anodes facilitate the conversion in an undivided cell. Bayesian optimization of the reaction enables combined diacid current efficiencies up to 53%, surpassing previous works. Technoeconomic analysis highlights economic potential of the conversion and future development needs.

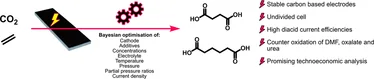

## 1 Introduction

Adipic (AA) and succinic (SA) acids are important building blocks for various chemical industries. Both monomers are mainly utilized in high‐volume polymers (e.g., PA, PU), as platform chemicals, or directly [1, 2, 3, 4, 5]. Currently, both acids are industrially produced oxidatively from petrochemicals—AA from KA‐oil (mixture of cyclohexanol and cyclohexanone) [1, 2, 3] and SA from butane or benzene through maleic anhydride or acid [6, 7]. Strikingly, adipic acid synthesis is responsible for 10% of global emissions of N_2_O, which is 300 times more potent greenhouse gas than CO_2_ [3], with total estimated emissions up to 8 ton of CO_2_ equiv. per ton of AA [8]. Comparably, the production of SA from fossil sources is estimated to emit 1.9 tons of CO_2_ per ton of product [8]. Consequently, environmental impacts of these emission‐intensive processes are amplified by their production volumes, which makes developing alternative synthesis routes a highly appealing target.

**SCHEME 1:**
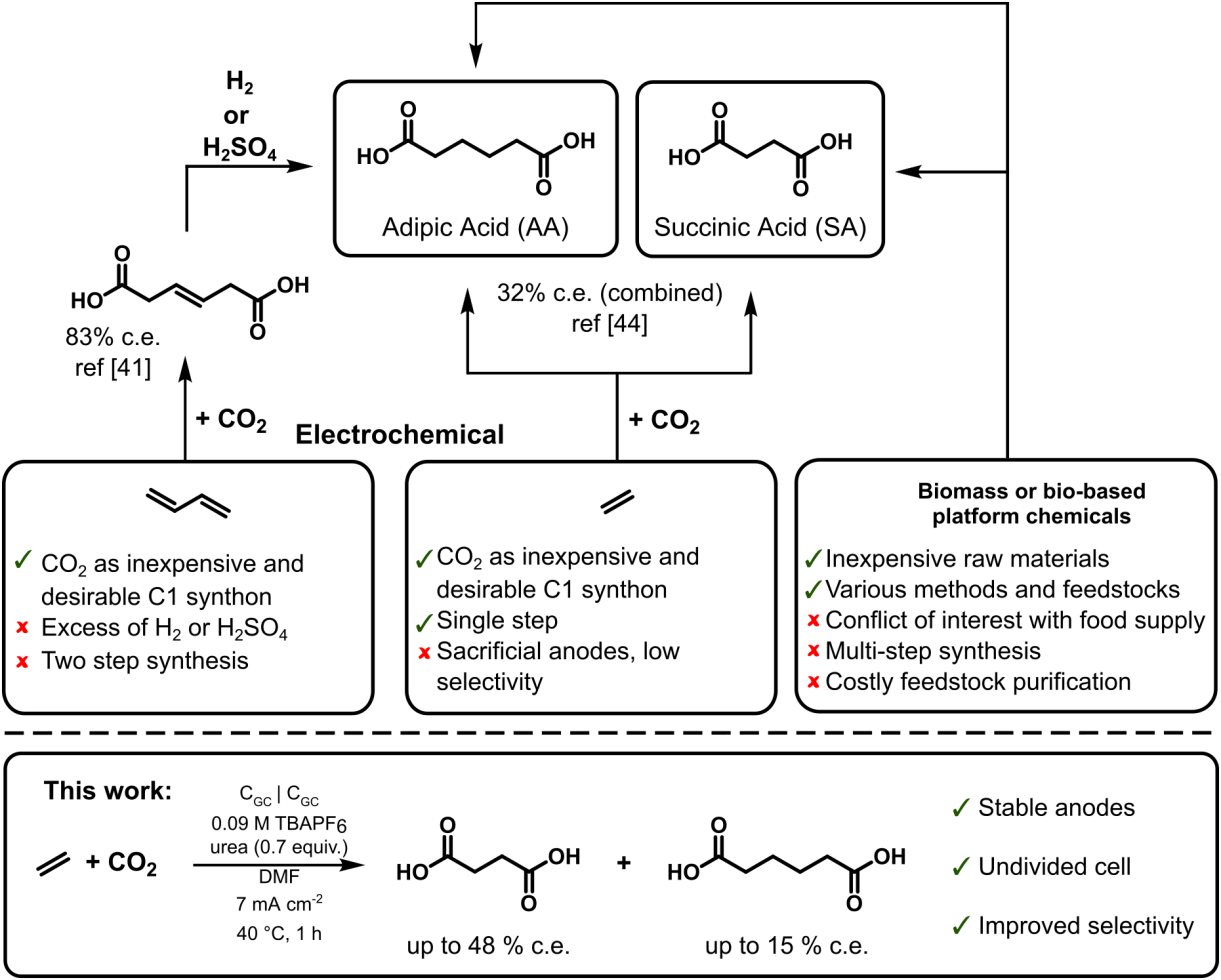
Various alternative routes for the synthesis of adipic and succinic acids from sustainable raw materials versus the scope of this work. c.e. indicates current efficiency.

Various bio‐based synthetic approaches have been reported for both SA and AA (Scheme 1, right). Microbial SA production from sugars has been demonstrated with several microorganisms, and SA and AA have also been successfully synthesized from sugars, (lignocellulosic) biomass and related platform chemicals [6, 7, 9, 10, 11, 12, 13]. Several of these routes have also been studied at pilot or small industrial scale [3, 7, 11]. Despite these developments, the production of both SA and AA from biomass remains challenging due to required feedstock pretreatment, product purification, and separation [3, 6, 10]. Moreover, the use of edible sugars as raw materials can create conflicts with food supply and land usage [14]. Thus, finding alternative starting materials and efficient synthesis routes for SA and AA would be desirable.

CO_2_ is a nontoxic, inexpensive, and widely‐available substrate for the synthesis of carboxylic acids [15, 16]. Accordingly, several strategies such as thermo‐, photo‐ and electrochemical methods have been explored for carboxylations of organic substrates [15, 19]. In this regard, electrochemical methods provide several advantages, such as mild reaction conditions, utilization of renewable energy, and scalability [20, 21, 22, 23, 24, 25, 26, 27, 28]. Furthermore, the direct utilization of electrons or electron holes for redox reactions provides economic, ecological and safe access to high‐energy intermediates by circumventing the need for stoichiometric redox reagents [29, 30], as has also been demonstrated with carboxylations targeting various industrially relevant (di)carboxylic acids [31, 32, 33, 34, 35, 36].

In the present context, most of the electrocarboxylations toward SA or AA have been either performed with ethylene or 1,3‐butadiene radical acceptors. For example, AA has been prepared by electrocarboxylation of butadiene to hexenedioic acid (Scheme 1, left) [34, 37, 38, 39, 40], followed by hydrogenation with an excess of hydrogen or by electroreduction in aqueous H_2_SO_4_. Similarly, SA can be obtained directly when ethylene is used as an acceptor (Scheme 1, middle) [41, 42]. Interestingly, it has been demonstrated that AA as well can be accessed from ethylene by increasing the total and ethylene partial pressures, which can lead to a chain growth of the propyl‐carboxylate radical intermediate formed during the reaction [43]. Importantly, all previous ethylene electrocarboxylations have been performed with sacrificial anodes [41, 42, 43], which undermines the feasibility of these protocols, an important aspect to consider in the synthesis of high‐volume commodity chemicals. Dissolving sacrificial anodes alter the interelectrode distance during electrolysis, and they are prone to surface passivation, which can increase the terminal voltage and electricity consumption over time [28, 44]. However, carboxylate anions are prone to oxidation at low potentials [32], and thus, careful design of anodic counter reactions is required to avoid the product decomposition in sacrificial anode free carboxylations.

We became interested in the electrosynthesis of SA and AA from CO_2_ and ethylene as the latter can be prepared from CO_2_ [45, 46], enabling synthesis of 100% CO_2_‐based SA and AA. The synthesis route has a potential to address the high environmental impact of manufacturing of both SA and AA, while their high annual combined volumes could significantly contribute to CO_2_ utilization in the chemical industry. Moreover, only modest combined current efficiencies for SA and AA (32%) have been reported to date [43]. Our main target in this study was to develop reaction conditions for stable anodes, which we see as a potential stepping stone toward electrochemical SA and AA production.

## 2 Results and Discussion

We commenced our studies in the simplest possible cell configuration, an undivided cell with a constant current operation. Since ethylene has a poor solubility in most organic solvents, we constructed a high‐pressure batch cell for promoting its solubility (SI). The reactor comprised of a stainless‐steel (SS) reactor cup, PTFE‐insert, and a lid equipped with isolated feedthroughs and gas line with a pressure meter. Electrodes were assembled into a 3D‐printed electrode holder (PEEK) inside the reactor (SI).

**FIGURE 1:**
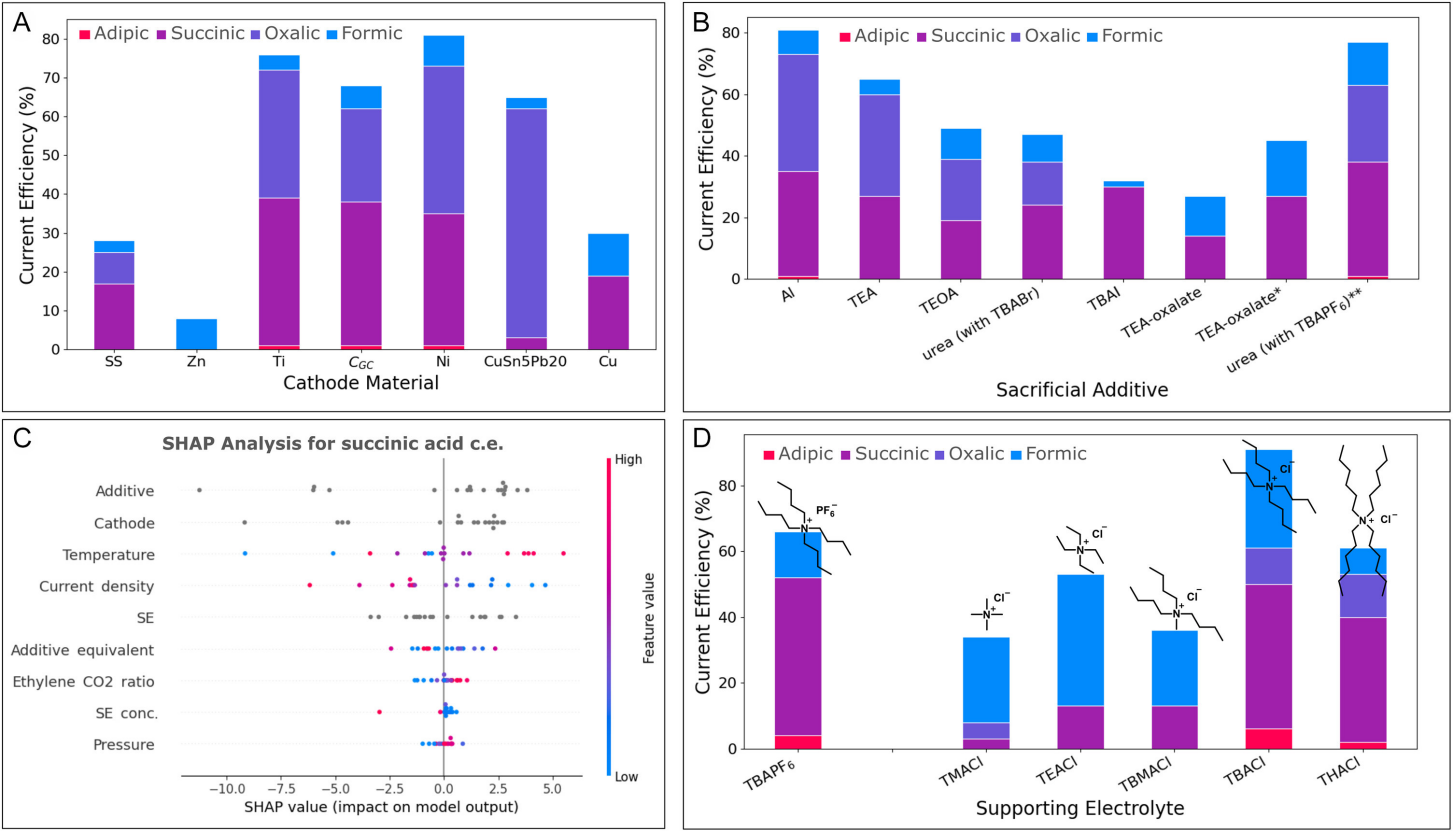
Optimization studies. (A) The impact of the cathode material on the product distribution on CO_2_ electroreduction in the presence of ethylene in DMF with Al anode and 30 mA cm^−2^ current density. (B) The impact of sacrificial reagents on sacrificial anodeless ethylene carboxylation with Ni cathode and C_GC_ anode at 30 mA cm^−2^ current density, benchmarked against use of Al anode. *C_GC_ cathode, 10 mA cm^−2^, 1,1′‐dimethylferrocene **C_GC_ cathode, 10 mA cm^−2^, TBAPF_6_ instead of TBABr. (C) Beeswarm plot of feature importance in succinic acid synthesis from CO_2_ and ethylene in DMF. (D) The effect of the supporting electrolyte cations on the product distribution, benchmarked against Bayesian optimized conditions with TBAPF_6_ supporting electrolyte.

We then set our objective for optimizing the current efficiency (c.e.) for SA and initiated our studies by screening various cathodes based on a previous mechanistic reasoning that the reaction proceeds via ${\mathrm{CO}}_{2}^{•-}$ [43], and that cathodes with a low electrocatalytic activity for CO_2_ reduction in aqueous media, such as SS, Ni, Ti, and C_GC_, can generate CO_2_ radical anions in organic aprotic solvents [47, 48, 49, 50, 51]. At this stage, sacrificial aluminum anode was used under 5 bar CO_2_ and 25 bar ethylene partial pressures in DMF containing 0.1 M tetrabutylammonium bromide (TBABr) as an electrolyte at a room temperature (Figure 1A). The best results were obtained with titanium (Ti), glassy carbon (C_GC_, Sigradur G, HTW Hochtemperatur‐Werkstoffe GmbH) and nickel (Ni) cathodes (1h, 30 mA cm^−2^), yielding SA in 38, 37, and 34% c.e., respectively. In addition, adipic acid (ca. 1% c.e.), oxalic acid and formic acid were detected in the reaction mixtures. Curiously, materials with higher electrocatalytic activity for CO_2_ reduction, such as Cu or Zn, resulted in more prominent over‐reduction to other products.

We then proceeded with replacing sacrificial anode by screening various molecular counter‐oxidations with low oxidation potentials. For this purpose, a stable C_GC_ anode and a Ni cathode were employed in DMF with 0.1 M TBABr as the supporting electrolyte under 5 bar CO_2_ and 25 bar ethylene using 30 mA cm^−2^ current density and 1 h electrolysis (Figure 1B). The highest c.e. for SA (30%) was obtained with tetrabutylammonium iodide (TBAI). Moreover, triethylamine (TEA, 27%), urea (24%), triethanolamine (TEOA, 19%), and tetraethylammonium oxalate (TEA‐oxalate, 14%) also furnished SA. We then continued our experiments with urea and oxalate oxidations. The former is a relatively inexpensive additive, and the latter could provide an anodic pathway for producing ${\mathrm{CO}}_{2}^{•-}$. Furthermore, both oxidations can produce CO_2_ although the urea oxidation has not been studied in detail in aprotic organic solvents, to the best to our knowledge [52]. For these experiments, we utilized 10 mA cm^−2^ c.d. and C_GC_ cathode under 4 bar CO_2_ and 40 bar ethylene. Interestingly, the oxalate oxidation, which was mediated with 1,1′‐dimethylferrocene [53], furnished 27% c.e. of SA (average of two experiments). Furthermore, urea additive provided an improved c.e. of 36% for SA, which was also demonstrated with other tetrabutylammonium counter anions. Replacing bromide anion with BF_4_^−^ and PF_6_^−^ provided SA in 35 and 38% c.e., respectively (Table S2, Entries 10 and 11).

We then proceeded to optimize SA c.e. by studying selected reaction conditions with Bayesian optimization (BO) protocol. For this purpose, we utilized recently developed open‐source BayBE framework [54]. Explored parameters consisted of additive (urea or no additive), the amount of additive (0–0.66 mmol vs. mmol electrons), supporting electrolyte type (TBABr or TBAPF_6_) and concentration (0.05–0.5 M), cathode material (SS, Ti, C_GC_, or Ni), current density (5–100 mA cm^−2^), ethylene to CO_2_ ratio (1–25), temperature (0–60 °C), and pressure (15 – 55 bar). After 13 experiments, we obtained SA and AA in 48% and 4% c.e, respectively (Table 1, Entry 1). SHAP (SHapley Additive exPlanations) analysis (Figure 1C) of the results, which explains how each feature impacts machine learning (ML) model prediction of the outcome (i.e., SA c.e.) [55], indicates that: (i) urea additive favors the formation of SA, (ii) C_gc_ provides higher SA c.e. in comparison to other examined cathodes, (iii) elevated temperatures (40 °C) and low current densities (5–10 mA cm^−2^) promote SA formation, and (iv) high pressure and ethylene to CO_2_ ratios have a positive correlation with SA c.e. However, the latter impact was less significant compared to the other parameters. The amount of additive did not have a clear correlation on SA c.e. (Figure 1C) despite having a medium impact on the ML model output. The impact of supporting electrolyte concentration was limited within the studied range.

**TABLE 1:** Control studies for the electroreduction of CO_2_ in the presence of ethylene.

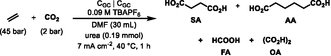						
Entry	Modification to the selected conditions	SA c.e., %^a^	AA c.e., %^a^	OA c.e., %^a^	FA c.e., %^a^, ^c^	Total c.e., %^a^, ^c^
1	None^b^	48	4	0	14	66
2	No electricity	0	0	0	0	0
3	No CO_2_	0	0	n.r.	14	14
4	No ethylene	0	0	62	7	69
5	0.5 vol% H_2_O	39	1	0	11	51
6	C_Gr_ anode	48	0	0	10	58
7	BDD anode	42	4	8	16	70
8	BDD cathode	46	7	0	18	71
9	C_Gr_ cathode	0	0	43	5	48
10	1 bar CO_2_, 50 bar ethylene	40	9	0	12	61
11	800 rpm stirring	38	7	0	14	59
12	Entries 8, 10, 11 combined	39	10	0	10	59
13	BDD cathode, 800 rpm, 1 bar CO_2_, 80 bar ethylene	26	15	0	7	48
14	No urea	40	7	0	19	66
15	Al anode, 1 bar CO_2_, 80 bar ethylene	61	5	0	0	66

*Note:* Pressures are reported as partial pressure of both ethylene and CO_2_.

Abbreviation: n.r., not recorded.

a Yields and current efficiencies were calculated from integrals of ^1^H NMR using 1,3,5‐trimethoxybenzene as an internal standard.

b Average of four experiments.

c Assuming only cathodic pathway for FA.

Next, we investigated what effect varying the quaternary ammonium cation (QAC) structure has for the SA c.e. (Figure 1D). For this purpose, we employed quaternary ammonium chlorides, which were more readily available. Tetrabutylammonium chloride (TBACl) provided SA (44%) and AA (6%) in c.e. similar to TBAPF_6_, indicating a small role from the counter‐anion. Curiously, when the length of the alkyl substituents in QAC was decreased, also SA c.e. diminished to 13% (TEACl) and 3% (TMACl). In addition, tributylmethylammonium chloride (TBMACl) reduced the SA c.e. to 13%. It has previously been shown that the adsorbance of hydrophobic substrates, such as acrylonitrile, into the electrode surface is enhanced by increasing the hydrophobicity of the electric double layer consisting of QACs [56]. Similarly, more lipophilic quaternary ammonium salts, such as TBA^+^ instead of TMA^+^ and TEA^+^, could increase the near‐electrode concentration of ethylene, which in turn could promote ethylene carboxylation over CO_2_ reduction to oxalate or formate. However, increasing the hydrophobicity of the supporting electrolyte further by employing tetrahexylammonium chloride (THACl) slightly reduced SA c.e. from 44% to 38%. Interestingly, it has been reported that the onset potentials for CO_2_ reduction in aprotic media shift negatively when the length of the alkyl substituents in QAC are increased [57, 58]. Therefore, we hypothesize that this shift in the onset potential may hinder CO_2_ reduction or promote other reduction processes. In the future, the fine‐tuning of the QAC properties could unlock higher selectivities for ethylene carboxylation by simultaneously promoting CO_2_ reduction and near‐electrode concentration of ethylene.

We then repeated the synthesis three times in selected BO conditions to probe the reaction reproducibility (Table 1, Entry 1). Standard deviation was 4% for SA, 3% for AA and 4% for FA. Thereafter, a series of control experiments was performed to provide additional insights on the synthesis. The reaction did not proceed either in the absence of electricity (Entry 2) or without CO_2_ or ethylene (Entries 3–4). In latter cases, formic acid (Entry 3) and oxalic acid (Entry 4) were obtained as the main products, respectively. The formic acid production and the lack of SA/AA formation in the absence of CO_2_ indicates that the formic acid might be formed via DMF decomposition and that carboxylic acids originate from CO_2_, respectively. The reaction can be performed also in the presence of 0.5 vol% water with slightly reduced c.e. 39% (Entry 5). Furthermore, other nonsacrificial anodes than C_GC_ can be employed, as demonstrated by the good performance of graphite (Sigrafine V2100, SGL Carbon) and boron‐doped diamond (BDD, Merck) anodes, which resulted in 48% and 42% SA c.e., respectively (Entries 6 and 7). However, no AA formation was observed with a graphite anode, whereas BDD provided the same c.e. for adipic acid as C_GC_ anode. Interestingly, BDD can also be employed as a cathode instead of C_GC_, providing SA in 46% c.e. and AA in 7% c.e. (Entry 8). However, the use of C_Gr_ cathode provided only oxalic (43%) and formic (5%) acids (Entry 9). The reaction can be tuned toward more pronounced production of AA (9% c.e.) by lowering CO_2_ partial pressure into 1 bar and increasing the ethylene partial pressure to 50 bar (Entry 10). Increasing the magnetic stirring speed from 500 to 800 rpm increased AA c.e. from 4% to 7% (Entry 11). When BDD electrodes, higher stirring speed and modified partial pressures are used in conjunction, AA c.e. of 10% was obtained (Entry 12). With BDD cathode, further increase of ethylene partial pressure to 80 bar resulted in production of SA and AA in 58:42 weight ratio and improved AA c.e. to 15% (Entry 13). Interestingly, the reaction also proceeds in the absence of urea, which resulted in slightly diminished c.e. from 48% to 40% for SA and slightly improved c.e. from 4% to 7% for AA (Entry 14). SA and AA are obtained in 61% and 5% c.e., respectively, when optimized conditions are used with an aluminum anode in conjunction with 80 bar ethylene partial pressure (Entry 15).

**FIGURE 2:**
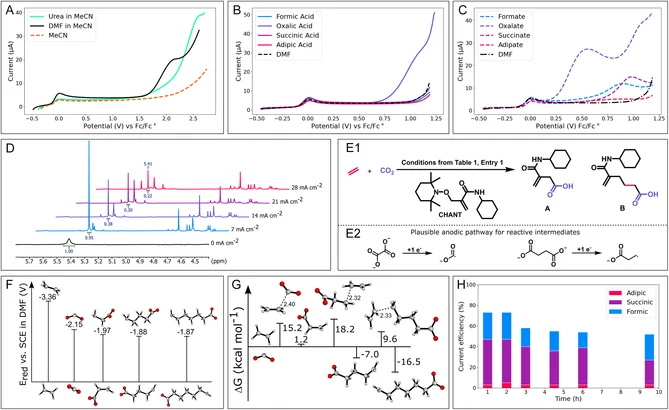
Mechanistic and control studies. (A) Oxidative CV of urea and DMF oxidation in MeCN. (B) Oxidative CV of carboxylic acid products in DMF. (C) Oxidative CV of carboxylates, generated *in situ* with NaOMe, in DMF. For clarity, only the forward scan of CV is presented in (A–C). CVs were recorded using C_GC_ working electrode, Pt anode, and Ag/Ag_2_S microreference electrode [59] and all the potentials are reported against Fc/Fc^+^ couple (D) ^1^H NMR studies on oxidation of urea and DMF as function of applied current densities. (E) 1: Radical trapping experiments with CHANT as the radical scavenger. 2: Plausible anodic pathways for formation of C_3_ and CO_2_ radical anions. (F) Calculated one‐electron reduction potentials versus SCE. (G) DFT reaction coordinate. (H) Distribution of current efficiencies over different carboxylic acid products over time period of 9.5 h.

The discrepancy between the impact of urea in ML‐model and control experiment w/o urea warranted us to perform additional experiments with cyclic voltammetry (CV). First, we employed 0.1 M TBAPF_6_ in DMF as the electrolyte with circular C_GC_ working electrode. However, the oxidation of urea could not be distinguished from the DMF onset potential (SI). Thus, we exchanged solvent from DMF to MeCN for its broader electrochemical window. In MeCN, DMF has a lower peak oxidation potential of 2.18 V (vs. Fc/Fc^+^) in comparison to 2.62 V (vs Fc/Fc^+^) of urea (Figure 2A). However, both compounds have similar onset potentials, which encouraged us to further study the oxidation under constant current electrolysis, where the applied potential is not controlled. We then performed the reaction by varying the current densities from 7 to 28 mA cm^−2^ using 0.12 mmol of urea per mmol of e^−^ during the electrolysis (Figure S2D). Interestingly, the urea conversion increased from 5% to 78% across the series, and the analysis of ^1^H NMR spectra displayed the emergence of various peaks with increased conversion. Furthermore, N_2_ formation was not detected from the reactor headspace with GC/MS, indicating that the urea oxidation products remain dissolved in DMF. Moreover, the attempts to concentrate the oxidation products visible in ^1^H NMR with rotary evaporator indicate that the compounds are volatile, which may simplify the downstream processing.

Further important aspect to consider is the product stability in an undivided cell, as it is known that the reductive carboxylations typically yield carboxylates as intermediates, which are more prone for oxidation than the carboxylic acids [32]. Therefore, we studied the oxidative stability of FA, OA, SA, and AA with CV in DMF. In the employed potential window of 0–1.65 V (vs. Ag/Ag_2_S), SA, AA and FA were stable, whereas OA was oxidized at onset potential of (1.26 V vs. Ag/Ag_2_S; 0.79 V vs. Fc/Fc^+^) (Figure 2B). Thereafter, we deprotonated the acids by adding 2 equiv. NaOMe into the electrolyte. Consequently, the oxidation potentials of carboxylates (OA = 0.55 V, FA = 0.89 V, SA = 0.99 V vs. Fc/Fc^+^) were significantly lower than their respective acids (Figure 2C). The oxidation potential of adipate could not be determined, presumably due to its poor solubility in DMF. Interestingly, oxalic acid was not detected in most of the carboxylation experiments. Based on CV, oxalic acid and oxalate dianion are oxidized before urea and DMF. Considering this, we propose that oxalate (or oxalic acid), that is formed during the reaction at the cathode, is later oxidized at the anode, which reduces the overall current efficiency of the synthesis by forming a chemical “short‐circuit”. Furthermore, also other carboxylates have lower oxidation potentials than DMF and urea, indicating that also they should be oxidized preferentially. However, up to 53% and 61% combined c.e. of SA/AA/OA are obtained (Table 1, Entry 8; Figure 1D, TBACl). This indicates that the carboxylates are protonated, or otherwise protected, from the oxidative degradation during the electrolysis. In our earlier study, we have observed that anodic DMF oxidation can supply protons for the reaction in α‐hydrocarboxylic acid synthesis [32]. In the current study, either DMF or urea oxidations, the ratio depending on the applied current density, could function similarly.

In order to provide additional mechanistic insight on the reaction we also have analyzed the reaction mixtures (Table 1, Entry 1) with HPLC‐ESI‐HRMS. In addition to SA and AA, we detected monocarboxylic (propionic and pentanoic), and dicarboxylic (octanedioic and decanedioic) acids in the reaction mixtures (Supporting Information). Intriguingly, the formation of propionic and pentanoic acids suggests indirectly the presence of C_3_ and C_5_ radical anions. Furthermore, when the reaction was conducted in the presence of a CHANT radical trap [60], which is known to be resistant against nonradical false positive pathways, we also detected the corresponding adducts of the C_3_‐radical anion (Figure 2E, **B**), and CO_2_‐radical anion (**A**), although we cannot rule out the formation of the latter by reduction of CHANT radical trap followed by coupling of generated radical anion with CO_2_.

The cathodic reaction mechanism was then further examined by combining previous experimental results with computational studies at PW6B95‐D3BJ/def2‐TZVPD/CPCM(DMF) // PBE0‐D3BJ/def2‐TZVP/CPCM(DMF) level of theory (Figure 2F‍–‍G. Tentatively, the reaction is initiated by CO_2_ reduction into CO_2_‐radical anion, which is experimentally supported by the accumulation of oxalic acid as a side product in several instances (e.g., with sacrificial anodes). In addition, calculated one‐electron reduction potentials indicate that the ethylene reduction potential (−3.36 V vs. SCE in DMF) is considerably more negative than that of CO_2_ (−2.15 V vs. SCE in DMF). The latter is in a good agreement with an experimental value of −2.21 V (vs. SCE) with Hg electrode in DMF [61]. The reaction then proceeds by a coupling of CO_2_‐radical anion and ethylene (Δ*G* ^TS^ = 15.2 kcal mol^−1^) in a slightly endergonic (Δ*G* = 1.2 kcal mol^−1^) process. This intermediate can be then terminated to succinate either by the reduction of the formed C_3_‐radical anion into a carbanion (−1.97 V vs. SCE in DMF), followed by a reaction with CO_2_ (EC mechanism), or via reaction with CO_2_ followed by reduction (CE mechanism). The former pathway has been reported for several electrochemical olefin carboxylations [36, 62, 63, 64], but the latter also seems to be energetically accessible (Δ*G* ^TS^ = 19.1 kcal mol^−1^, Δ*G* = 19.0 kcal mol^−1^; Figure S44). Alternatively, the propyl‐carboxylate radical can react with ethylene to form (CH_2_CH_2_)_*n*_ chain extension products with Δ*G* ^TS^ of 18.2 (*n* = 1) and 9.6 kcal mol^−1^ (*n* = 2).

However, presently, we cannot rule out that the detected radical adducts **A** and **B** (Figure 2E1), which were detected in minute quantities with HPLC/ESI‐HRMS, are not formed through anodic Kolbe‐type oxidation of oxalate and succinate (Figure 2E2). In this respect, the following experiments indicate against the involvement of Kolbe‐pathway in AA synthesis. First, adipic acid formation is observed with sacrificial Al anode (Table 1, Entry 15). Second, we did not detect any formation of adipic acid when dipotassium succinate was electrolyzed in the reaction conditions in the absence of CO_2_ and urea. Third, when reaction is stopped, cell is depressurized, and electrolysis is then continued in the absence of ethylene, no further AA formation was detected (Table S9).

The economic feasibility of the synthesis was investigated by examining operating and capital expenses of both the electrosynthesis and a (hypothetical) downstream separation (for further details, see Supporting Information). Several assumptions (e.g., selective urea counter oxidation) and projections (e.g., increased acid concentration) were made due to the open questions at this stage of the development. The process was found to be largely OPEX‐driven, with raw material and electricity costs being major contributors to the final cost of SA and AA produced. Based on these considerations, the technoeconomic analysis looks quite promising, and conversion of captured CO_2_ and ethylene into a mixture of succinic, adipic, and formic acids could become economically viable. Notably, production costs of SA and AA were found to be below 2000 € t^−1^, which puts them on par with current fossil‐based products (Figure S34).

Based on the techno‐economic assessment, we wanted to investigate the reaction performance in longer reaction times by extending the reaction to 9.5 h (Figure 2H). During the first 6 h, the current efficiency slowly decreases from 44% to 36%, and after that, the current efficiency is diminished further to 24%. Current efficiency for AA remained at 3% even at 9.5 h. The experiment provided final concentration of 0.07 M of SA:AA, and 0.1 M if continued for 24 h (see further discussion in Supporting Information). Alongside decrease in current efficiencies, we observed the formation of deposition on the cathode surface (Figure S9), when the cell was opened after 6 h. Interestingly no deposition was observed in experiments after 1 h, suggesting that deposition could be originate from the reduction of electrogenerated species, such as urea or DMF oxidation products.

## 3 Conclusion

In this study we have reported that ethylene and CO_2_ can be efficiently converted into succinic and adipic acids in an undivided high‐pressure electrolysis cell using stable electrodes. Bayesian optimization of the reaction conditions delivered combined current efficiencies up to 53% for succinic and adipic acids, which is a considerable improvement over previous works with sacrificial anodes (27%–32%) [41, 43]. Our results show that the urea can function as an inexpensive sacrificial additive in cathodic aprotic electrosynthesis; however, further studies need to be performed for the full deciphering of its role and to characterize the anodic oxidation products of urea. Mechanistic studies indicate that the reaction is initiated via the reduction of CO_2_ followed by reaction with ethylene. Techno‐economic assessment demonstrates that the ethylene dicarboxylation is an economically feasible route to synthesize both adipic and succinic acids. We are currently pursuing for increasing the current densities in the synthesis and optimizing the reaction conditions toward AA and higher molecular weight dicarboxylic acids that were detected with HPLC‐ESI‐HRMS measurements.

## Author Contributions

**Tom Wirtanen**: conceptualization (equal), investigation (supporting), methodology (equal); supervision (lead), writing – original draft (supporting), writing – review and editing (equal). **Valtteri Oksanen** conceptualization (equal), investigation (lead), methodology (equal), writing – original draft (lead), writing – review and editing (equal). **Markus Luukkonen:** investigation (supporting). **Kiia Malinen**: investigation (supporting). **Niko Heikkinen:** investigation (supporting). **Alexander Reznichenko**: supervision (supporting); writing – review and editing (supporting).

## Funding

This study was supported by Academy of Finland (348889).

## Conflicts of Interest

The authors declare no conflicts of interest.

## Supporting information

The authors have cited additional references within the Supporting Information [65, 66, 67, 68, 69, 70, 71, 72, 73, 74, 75, 76, 77, 78, 79, 80].

## Data Availability

The data that supports the findings of this study are available in the supporting information of this article.
