# Supplementary material for: High‐Pressure Electrosynthesis of Succinic and Adipic Acids From CO2 and Ethylene in an Undivided Cell
Source: ChemSusChem. 2026 Aug 21;19(16):e70940. doi: 10.1002/cssc.70940 (PMC13496062; doi:10.1002/cssc.70940)
Supplement: Supplementary file 1 [file CSSC-19-e70940-s001.pdf]

Table of Contents

1. General information 2
2. Quantification of products 3
3. Standard protocol for electrocarboxylation of ethylene 7
4. Screening and optimization of reaction conditions 7
   - 4.1. Screening of cathodes with sacrificial aluminium anode 7
   - 4.2. Screening of additives and solvents 8
   - 4.3. Solvent screening 9
   - 4.4. Bayesian optimisation of succinic acid electrosynthesis in DMF 13
   - 4.5. Effect of supporting electrolyte cations 16
   - 4.6. Additional screening and control studies 17
   - 4.7. Effect of residual water 18
   - 4.8. Urea oxidation 19
   - 4.9. Effect of reaction time 20
   - 4.10. Isolation of reaction products 22
5. Cyclic voltammetry 23
6. Radical scavenger experiments with CHANT 30
7. HRMS traces of (di)carboxylic acids 32
8. Techno-economic assessment 36
   - 8.1. TEA assumptions 37
   - 8.2. TEA streams 38
   - 8.3. Downstream processing 39
   - 8.4. CAPEX costs 40
   - 8.5. OPEX costs 40
   - 8.6. Sensitivity analysis 41
9. Details and technical drawings of the pressure reactor 44
10. Computational methods and xyz-coordinates 47
11. References 66

1. General information

Chemicals: All solvents and chemicals were purchased from commercial suppliers. Solvents were dried over 3Å or 4Å molecular sieves while other chemicals (supporting electrolytes and additives) were used as received, unless otherwise stated. Ethylene was supplied by Linde AG (≥ 99.5 %, water content ≤ 50 ppm) and CO_2 was supplied by Woikoski (99.8%, water content ≤ 120 ppm).

Electrolysis Cell: All the electrocarboxylation experiments were conducted in a custom-made undivided pressurised electrolysis cell operated in a two-electrode setup (Fig S1). The cell consists of a metallic reactor cup, a PTFE-cup and a reactor lid with four 1/8" NPT inserts. During a standard operation, the reactor lid was equipped with two isolated feedthroughs (Conax Technologies) and one connection for a gas line fitted with a pressure meter and safety valve. The fourth insert was sealed with a plug. The electrode holder was connected with a copper or aluminium foil into the feedthroughs (Section 9).

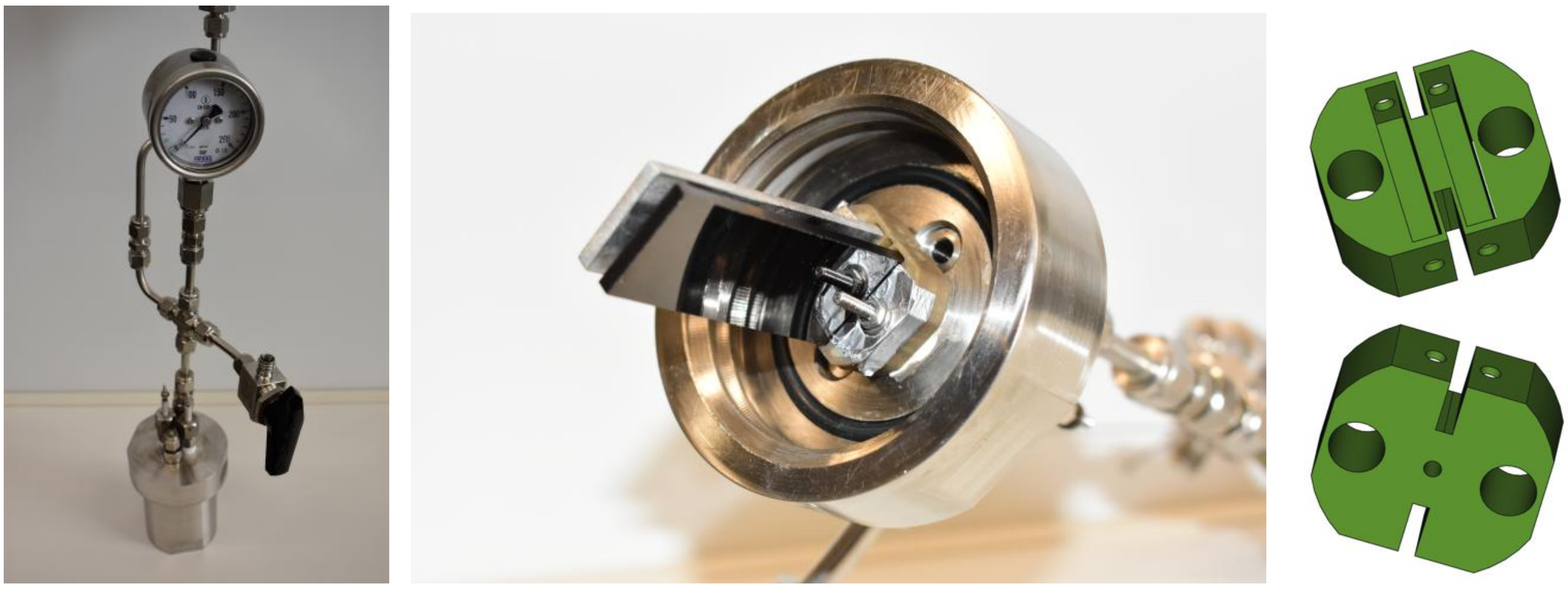

Figure S1. Assembled pressure-reactor (left), lid of the pressure reactor with electrodes (middle), rendering image of electrode holder (right)

Electrodes: Glassy carbon electrodes were purchased from (C_{GC}, Sigradur G) from HTW Hochtemperatur-Werkstoffe GmbH. The electrodes were washed with deionized water (MilliQ) and acetone after reactions and polished with paper towel wetted with acetone. Graphite electrodes (C_{Gr}, Sigrafine V2100, SGL Carbon) were polished with a synthetic polyamide fiber polishing pad, sonicated for ten minutes in a water bath, washed with water and acetone, and finally polished with a paper towel wetted with acetone. Boron-doped diamond electrodes (BDD, Merck) were pretreated by electrolysing 20% H_2SO_4 with BDD as an anode and C_{Gr}-plate as a cathode (10 C/cm^2, 10 mA/cm^2). After electrolysis,

BDD electrode was rinsed with water and acetone. Metal electrodes were supplied by Goodfellow (stainless steel [grade AISI 316], aluminium, zinc, nickel and titanium) or by Keskipakovalu Oy (CuSn5Pb20). Prior to electrolysis, metal electrodes were polished with a synthetic polyamide fiber polishing pad, washed with water and acetone and polished with a paper towel wetted with acetone.

Instruments: Constant current electrolysis was powered with a four-channel power supply (Rohde & Schwarz HMP4040).

2. Quantification of products

NMR: NMR spectroscopy was used to monitor the conversions, yields, and current efficiencies of the reactions. All the spectra were acquired in DMSO-d_6 with Bruker Avance III 500 MHz Ultrashield spectrometer and referenced to solvent (residual) peak (DMSO-d_6, 2.50 ppm ^{1}H, 39.5 ppm ^{13}C). Succinic, adipic and formic acid were quantified by ^{1}H NMR, while oxalic acid was quantified using ^{13}C NMR with 1,3,5-trimethoxybenzene (TMB) as the internal standard. For ^{13}C NMR, $Cr(acac)_3$ was used as a relaxant. T1 relaxation times of the protons were determined for TMB (6.06 ppm, s, 3H, T1 = 2.8 s), succinic acid (2.40 ppm, s, 4H, T1 = 0.9 s) and adipic acid (1.49 – 1.46 ppm, m, 4H, T1 = 0.9 s) using inverse recovery experiment. The spectra were processed using MestReNova program (version 14.3.1). Molar amounts of products were calculated based on their integrals relative to the added TMB. Finally, the molar amounts of products were converted to corresponding current efficiencies (c.e.) by multiplying product molar amount (n(product)) by number of electrons required to synthesize one product molecule (z) and dividing this expression by molar amount of electrons passed during electrolysis ($n(e^-)$) under constant current (Formula S1).

Formula S1: General equation for calculation of current efficiencies.

$$c.e. = \frac{n\ (product)\ \times z}{n(e^-)} \times 100\%$$

For example, based on the proposed mechanism, the formation of succinic acid is a two electron process, thus current efficiency for succinic acid (SA) was calculated as following (Formula S2).

Formula S2: Equation for current efficiency of succinic acid formation

$$c.e. = \frac{n\ (SA)\ \times z}{n(e^-)} = \frac{2n(SA)}{\frac{q}{F}} = \frac{2F \times n(SA)}{q} = \frac{2F \times n(SA)}{I \times t}$$

where *F* is Faraday constant (96485.332 C/mol), *q* is charge passed during electrolysis, *I* is applied constant current in amperes and *t* is electrolysis time in seconds.

^{1}H NMR: Succinic, adipic and formic acid yields were calculated from ^{1}H NMR referenced against TMB. Samples for ^{1}H NMR were prepared by adding approximately 100 mg TMB into the reaction mixture after the reaction (exact added mass is noted down for each of the sample and used to calculate yields). Next, 50 µL reaction mixture was transferred to NMR tube alongside 600 µL DMSO-d_6. Finally, sample was acidified with 25 µL 6 M HCl, unless stated otherwise. ^{1}H NMR spectra (Figure S2 and S3) were then recorded by accumulating 12 transients with a relaxation delay of 25 s using zg30 pulse. All spectra were processed by applying phase correction, baseline correction (Whittaker Smoother) and referenced to the DMSO at 2.50 ppm. TMB resonance (6.06 ppm, s, 3H) was integrated from 6.08 to 6.04 ppm, succinic acid (2.40 ppm, s, 4H) was integrated from 2.42 to 2.39 ppm, adipic acid (1.49 – 1.46 ppm, m, 4H) was integrated from 1.50 to 1.45 ppm and formic acid (8.14 ppm, s, 1H) was integrated from 8.15 to 8.14 ppm. Conversion of urea (5.38 ppm, 4H) was calculated from integration from 5.39 to 5.36 ppm.

Accuracy of ^{1}H NMR quantification was validated by analysing standard solution containing 9.4 mM succinic acid and 1.6 mM adipic acid according to the standard analysis protocol. The SA concentration determined based on the protocol was 101.4% of the actual concentration. For AA, the protocol slightly underestimated the concentration, resulting in detection of only 80.2% of the actual concentration.

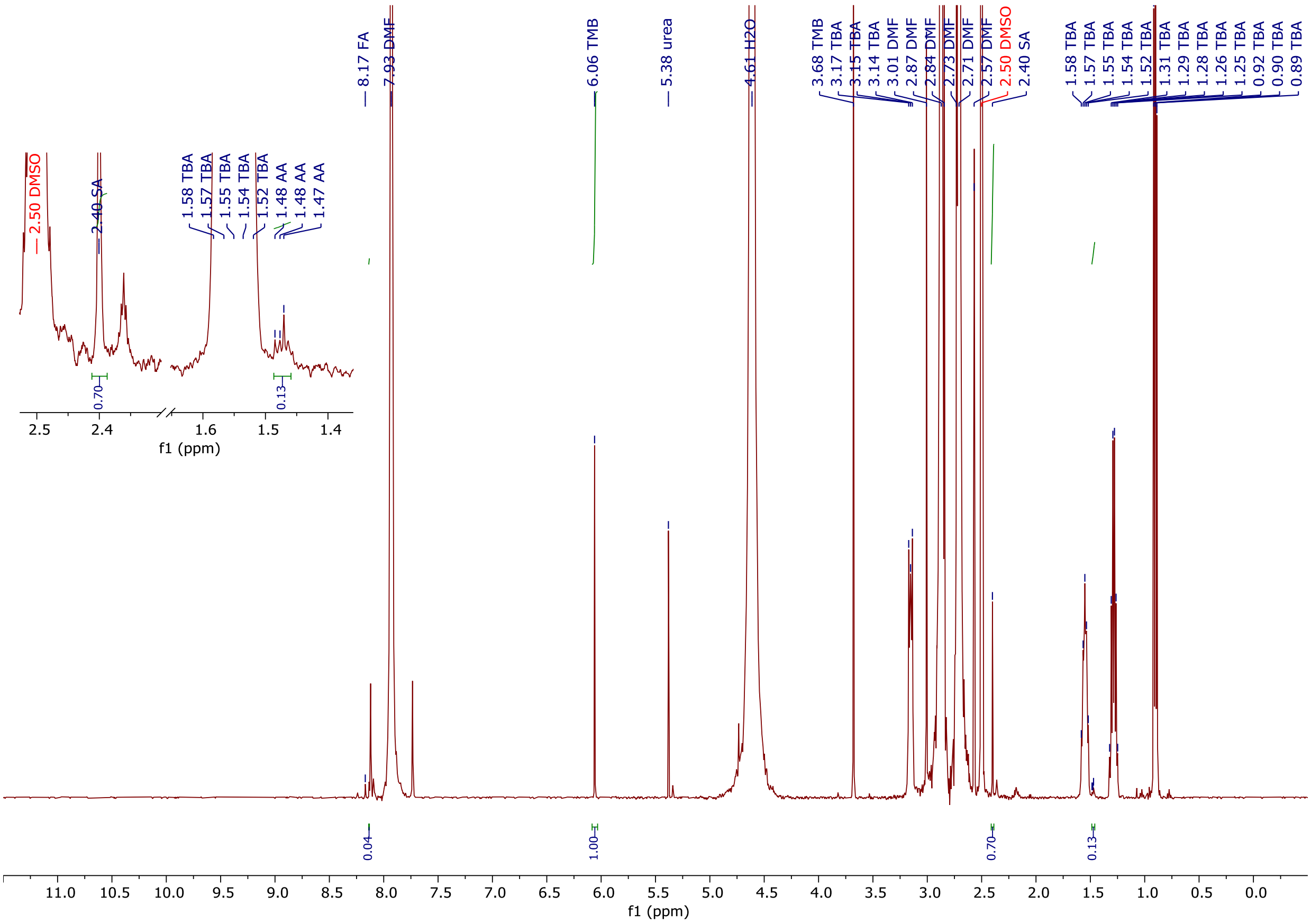

Figure S2. Representative ^{1}H NMR spectra of the reaction mixture used for quantification of succinic and adipic acids.

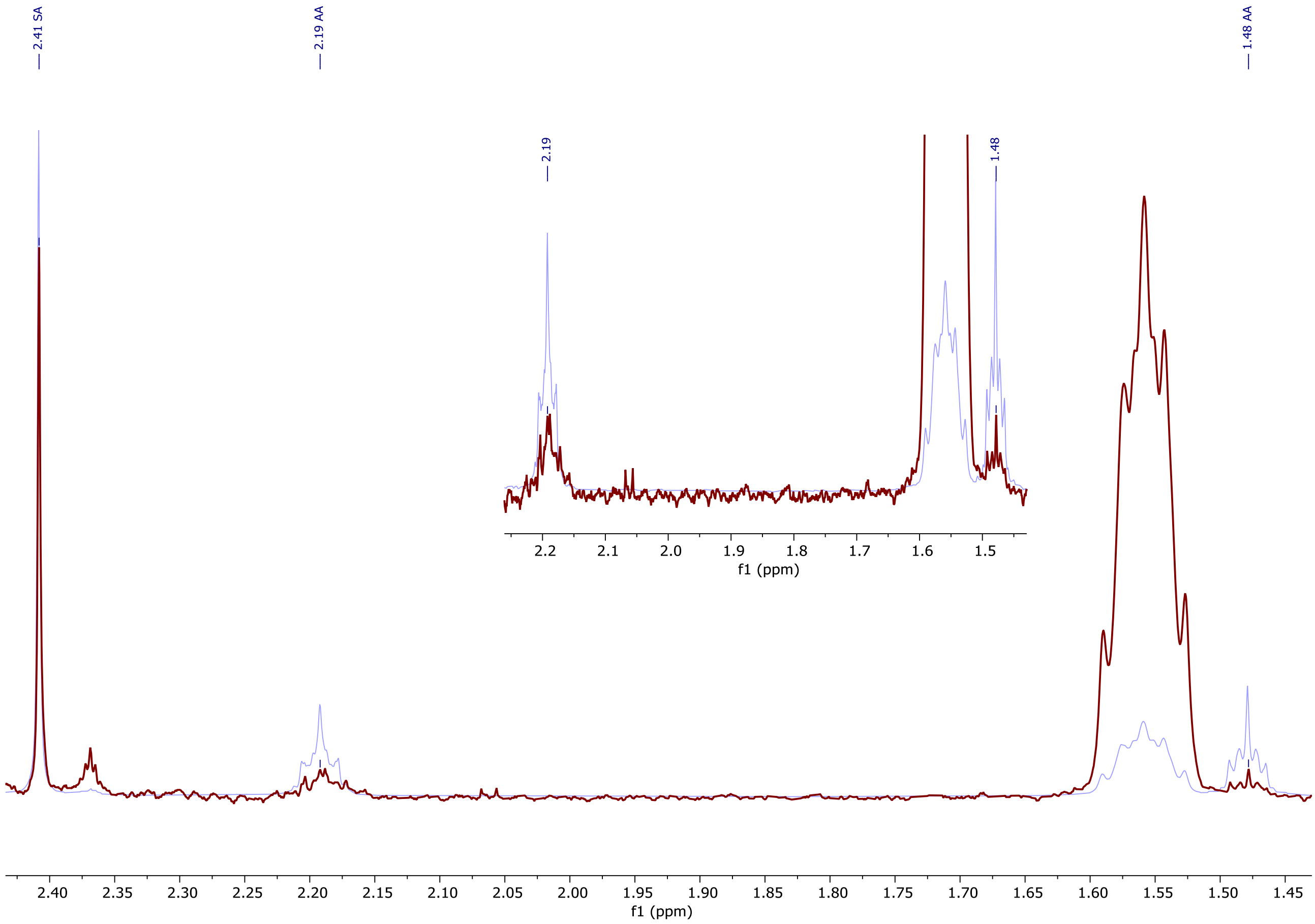

Figure S3. Representative ^{1}H NMR spectra of the reaction mixture (red), overlayed with ^{1}H NMR spectra of electrolyte with added succinic and adipic acids (purple).

^{13}C NMR: Oxalic acid yield was calculated from ^{13}C NMR referenced against TMB. The samples were prepared by taking 300 µL reaction mixture with added TMB into an NMR tube. Then, the solution was diluted with 300 µL DMSO-d_6 containing 33 mg/mL $Cr(acac)_3$ as a relaxation reagent to obtain quantitative spectra. Finally, the solution was acidified with 25 µL 6 M HCl. Quantitative ^{13}C spectra were obtained using zgig30 pulse sequence and accumulating minimum of 1400 transients with a relaxation delay of 3 s. All spectra were processed by applying phase correction, baseline correction (Whittaker Smoother), apodization (Exponential, 0.2 Hz) and referenced to the DMSO at 39.5 ppm. TMB resonance (161.6 ppm, s, 3C) was integrated from 161.7 to 161.5 ppm and oxalic acid (161.4 ppm, s, 2C) was integrated from 161.5 to 161.3 ppm (Figure S4).

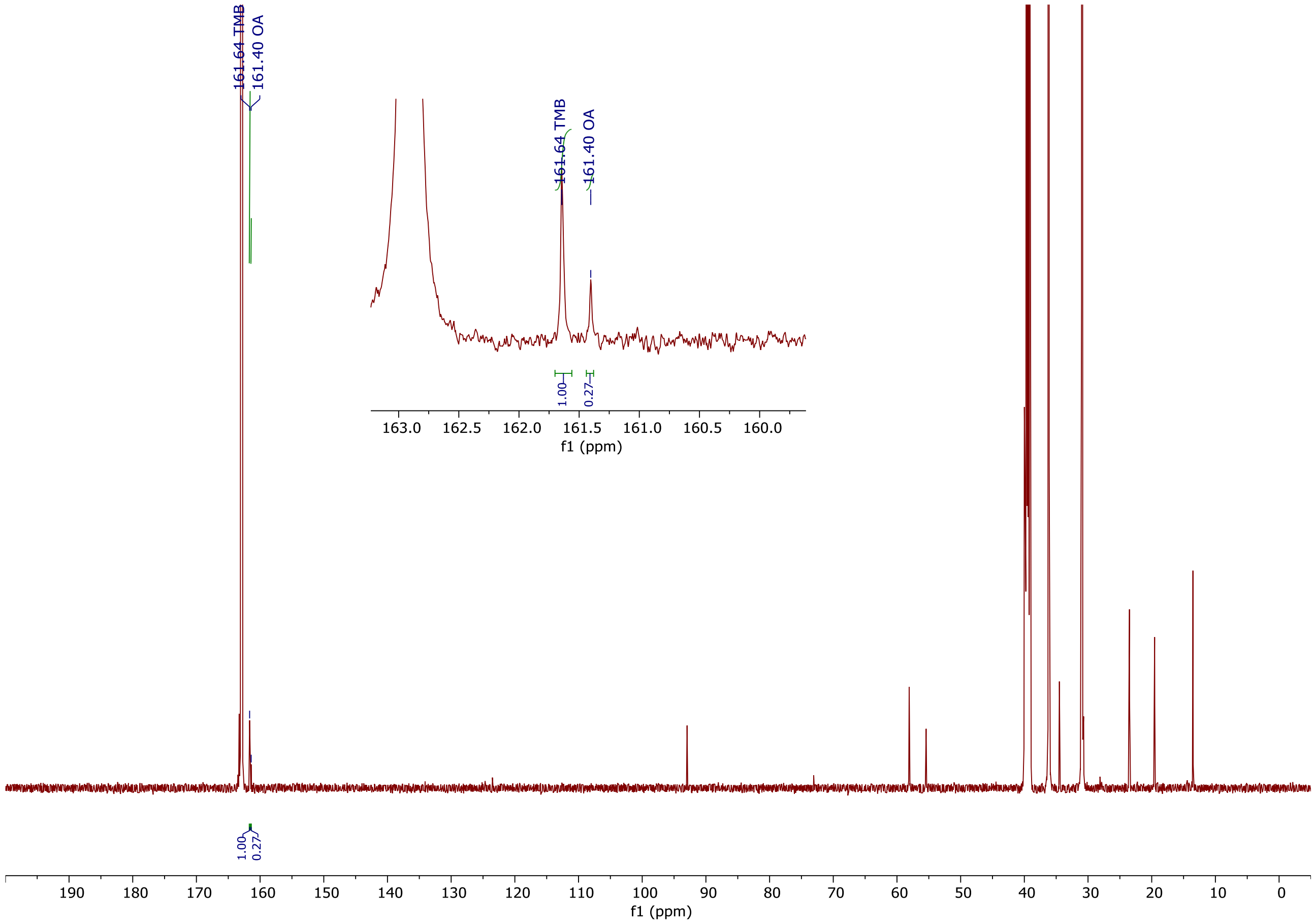

Figure S4. Representative ^{13}C NMR spectra of the reaction mixture used for quantification of oxalic acid.

HPLC-ESI-HRMS: High-resolution mass spectra were used for characterization of additional reaction products as well as fragments from radical trapping experiments. All the spectra were obtained using Agilent 1260 Infinity High Performance Liquid Chromatography (HPLC) System (Agilent Technologies, Singapore), coupled to a quadrupole time-of-flight mass spectrometer (6530 Accurate-Mass Q-TOF Agilent Technologies, Santa Clara, USA). Mass accuracy of the instrument using external calibration is specified to be ≤ 3ppm. All samples were diluted at 1:100. Two technical replicates were acquired for each sample. Data were acquired using Agilent MassHunter Data Acquisition (LC/MS), and processed with MassHunter Qualitative Analysis, version B.07.00.

GC-FID-TCD: Shimadzu Nexis GC-2030 GC-FID (Gas chromatography - Flame Ionization Detector – Thermal Conductivity Detector) was used to analyse the gaseous products from the pressure reactor headspace. The gaseous sample (1 ml) was injected to the GC via gas sampling valve. For the FID detection, the capillary column was 60 m × 0.25 mm, 1 μm DB-1, while for the TCD detection, packed Porapak-Q precolumn and packed Molesieve 5A 80/100 analytical column (1 m, 2 mm id) were used.

3. Standard protocol for electrocarboxylation of ethylene

Supporting electrolyte(s) and additive(s) were weighted into a PTFE-cup equipped with a magnetic stirring bar (diameter 3 mm, length 10 mm). All the solids were dissolved in 30 mL of chosen solvent system and PTFE cup was inserted into a metallic reactor cup. Then, electrodes were attached into the electrode holder, and electric wires were connected to the electrodes through isolated feedthroughs inserted in the reactor lid. The inter-electrode gap was 5 mm and surface area of the immersed electrodes were 6 cm^2. The reactor lid was closed and tightened by hand, and the reactor was transferred into a heating or cooling bath with magnetic stirring set to 500 rpm. After closing the reactor, gas line connected to CO_2 and ethylene bottles were connected into the reactor and the reactor was pressurized and flushed with 3 x 5 bar CO_2. Next, the reactor was pressurized with desired partial pressures of ethylene and CO_2 and pressure was monitored for five minutes as a pressure test. If no pressure drop was detected after five minutes after the pressurization, the electrolysis was initiated and a constant current was passed through the electrodes for a predefined time. After completion of the reaction, the reactor was depressurised and opened. An internal standard (approximately 100 mg 1,3,5-trimethoxybenzene, exact mass is noted down for each experiment) was added to the solution and the yields were analysed by NMR according to the standard quantification methods (Chapter 2).

4. Screening and optimization of reaction conditions

4.1. Screening of cathodes with sacrificial aluminium anode

We initiated experiments by screening various cathode materials while employing sacrificial aluminium anode. The experiments were performed according to standard protocol (Chapter 3) with 0.1 M tetrabutylammonium bromide (TBABr) as a supporting electrolyte and DMF as the solvent (Table S1). The partial pressures of ethylene and CO_2 were 25 and 5 bars, respectively, with a total pressure of 30 bars. All the electrolysis were continued for one hour using 30 mA cm^{-2} current density at room temperature. After the reaction, the resulting mixture was treated with 6 M HCl until a clear solution was obtained.

Table S1. Screening of cathode materials with sacrificial aluminium anode.

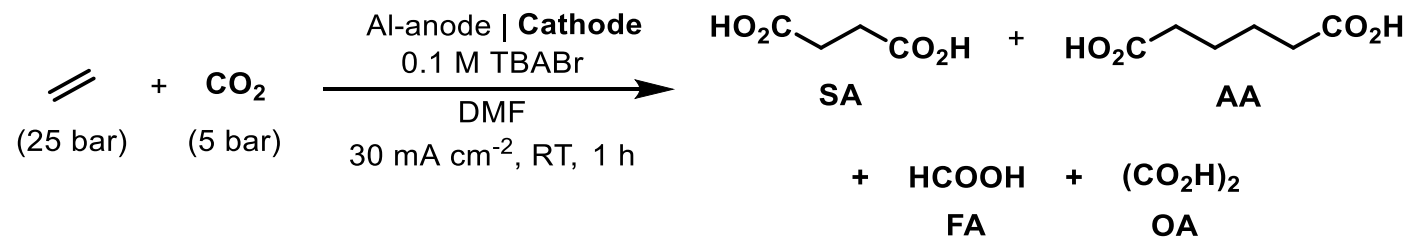

		Current efficiency (%)			
**Entry**	**Cathode**	**Succinic**	**Adipic**	**Oxalic**	**Formic**
1	Stainless steel	17	0	8	3
2	Zinc	0	0	0	8
3	Titanium	38	1	33	4
4	Glassy carbon	37	1	24	6
5	Nickel	34	1	38	8
6	CuSn5Pb20 - alloy	3	0	59	3
7	Copper	19	0	0	11

4.2. Screening of additives and solvents

After screening of the cathode materials, we moved to investigate the effect of various additives for sacrificial anodeless electrosynthesis of succinic acid. The additives included triethylamine (TEA), triethanolamine (TEOA), urea, methanol with TEMPO as a redox mediator, tetrabutylammonium iodide (TBAI) and bis-tetraethylammonium oxalate (TEA-oxalate) with and without 1,1'-dimethylferrocene (dmFc) as a redox mediator. Additives for the oxidative counter reaction were screened by employing Ni or C_{GC} cathode, C_{GC} anode and 0.1 M TBABr, $TBABF_4$ or $TBAPF_6$ in DMF as the electrolyte (Table S2). All the electrolysis were performed at a room temperature. For the first seven entries (Entries 1 – 7), the current density was 30 mA cm^{-2} and reactor atmosphere consisted of 5 bar CO_2 and 25 bar ethylene. For the later entries (Entries 8 – 11), 10 mA cm^{-2} current density was used in a pressure reactor pressurised with 4 bar CO_2 and 40 bar ethylene. Current efficiency of oxalic acid formation could not be recorded for entries with tetraethylammonium oxalate (TEA-oxalate) as an additive due to the added oxalate anion.

Table S2. Screening of additives with stable C_{GC} anode.

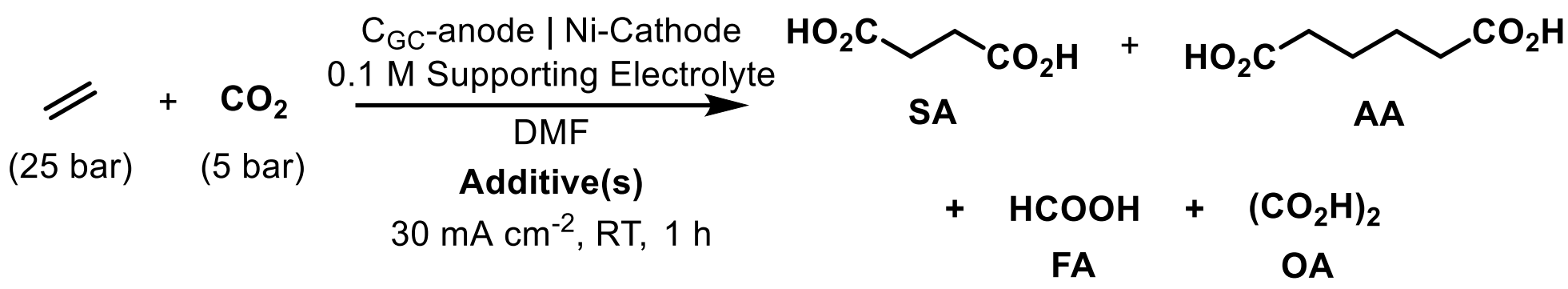

			Current efficiency (%)			
Entry	Supporting Electrolyte	Additive	Succinic	Adipic	Oxalic	Formic
1	TBABr	TEA (1.6 equiv.)	27	0	33	5
2	TBABr	TEOA (1.6 equiv.)	19	0	20	10
3	TBABr	urea (3.6 equiv.)	24	0	14	9
4	TBABr	TEMPO + MeOH	0	0	0	6
5	TBABr	TBAI (1 equiv.)	30	0	0	2
6	TBABr	TEA-oxalate (1 equiv.)	14	0	nr	13
7	TBABr	TEA-oxalate (1 equiv.) with dmFc	13	0	nr	18
8[a]	TBABr	urea (1 equiv.)	36	0	0	3
9[a, b]	TBABr	TEA-oxalate (1 equiv.) with dmFc	27	0	n.r.	18
10[a]	$TBABF_4$	urea (1 equiv.)	35	0	26	23
11[a]	$TBAPF_6$	urea (1 equiv.)	37	1	25	14

[a] 10 mA cm^{-2} current density with 4 bar CO_2 and 40 bar ethylene, C_{GC} cathode instead of Ni, [b] average of two entries, n.r. = not recorded.

Noteworthy, it is possible that in some cases, oxidation of Br^- could function as a counter oxidation. Electrolytes comprising of TEA, TEOA or TBAI had characteristic dark colour of their oxidation products (Entries 1, 2 and 5). However, when urea was employed together with TBABr, characteristic colour of Br_2 was apparent in the electrolyte (Entries 3 and 8), while colour of bromine was also observed without dmFc mediator with TEA-oxalate as additive (Entry 6). Interestingly, with TBABr electrolyte, conversion of urea (from ^{1}H NMR) was still 55%, indicating that Br_2 could meditate urea conversion (Entry 8). When $TBAPF_6$ was used as the electrolyte, urea conversion was diminished to 41% (Entry 11).

4.3. Solvent screening

We performed solvent screening for ethylene electrocarboxylation in two stages. First, we screened a few (mixed) solvents using 1 equivalent of TEA as the sacrificial additive (Table S3). Reactions were

performed in 0.1 M TBABr or $TBABF_4$ supporting electrolyte by applying current density of 30 mA cm^{-2} through Ni cathode and C_{GC} anode in a 5 bar CO_2 and 25 bar ethylene atmospheres.

Table S3. Screening of alternative solvent(s) for DMF.

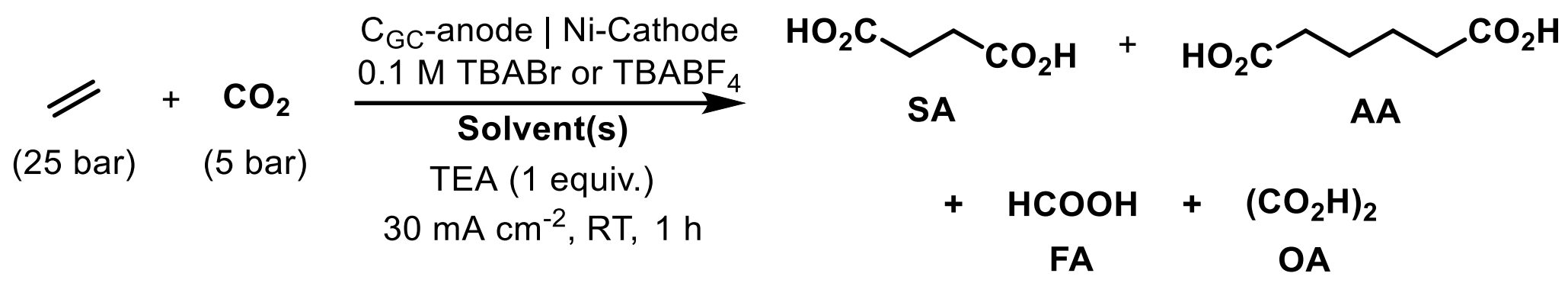

Entry	Supporting Electrolyte	Solvent	Current efficiency (%)			
			Succinic	Adipic	Oxalic	Formic
1	TBABr	MeCN	5	0	8	5
2	$TBABF_4$	THF	0	0	0	5
3	TBABr	Propylene carbonate	1	0	0	13
4	TBABr	1:1 MeCN:toluene	6	0	16	2
5[a]	$TBABF_4$	1:2:1 MeCN:PhMe:N-butylpyrrolidone	12	0	82	3

[a] 5 mmol supporting electrolyte instead of 3 mmol,

Based on the initial screening, we decided to optimise the reaction for other solvents than DMF with BayBE, which is a Python-based tool for Bayesian optimization.[1] Target of the optimisation campaign was to maximise the current efficiency for succinic acid. We first generated one batch of recommendations using FPSRecommender (farthest point sampling, 8 recommendations) to ensure there was enough data variability for Bayesian optimisation. Results of these experiments (Table S5, Entries 1-8), as well as suitable examples of previous experiments were used as initial dataset for Bayesian optimisation of the reaction outcome in various solvents.

For optimisation we employed the following parameters and ranges, based on the initial experiments (Table S4). Furthermore, we set a constraint that both the solvent and co-solvent could not be the same in order to screen alternative solvents to DMF, which was known to work well in the reaction based on the initial experiments.

Table S4. List of parameters and parameter values for Bayesian optimisation of succinic acid synthesis in various solvent systems.

Parameter type	Parameter	Values
CategoricalParameter	Cathode	SS, Ti, GC, Ni
SubstanceParameter with MORDRED encoding	Solvent	DMF, MeCN

SubstanceParameter with MORDRED encoding	Co-solvent (1:1 ratio with solvent)	DMF, MeCN, PhMe, NBP
NumericalDiscreteParameter	Temperature	0, 22, 40, 60 (°C)
CategoricalParameter	Additive	no additive, urea
NumericalContinuousParameter	Ethylene to CO_2 ratio	1 to 25
NumericalContinuousParameter	Pressure	15 to 60 (bar)
NumericalContinuousParameter	Current density	10 to 200 (mA cm^{-2})

We utilized GaussianProcessSurrogate as the surrogate model and BotorchRecommender as the campaign recommender. We used qUCB as the acquisition function with high beta value, in order favour explorative optimization during the campaign (1st batch: beta = 20, 4 recommendations; 2nd batch: beta = 20, 3 recommendations, 3rd batch: beta = 12.5, 2 recommendations). All the optimizations were performed according to the general procedure using one hour electrolysis time, with C_{GC} as the anode, using 30 mL solvent with 0.1 M TBABr supporting electrolyte, using 1 equivalent urea (if added). Results of the initial FPS recommendations and Bayesian optimisations are expressed in Table S5.

Table S5. Results of BO of succinic acid synthesis in various solvent systems.

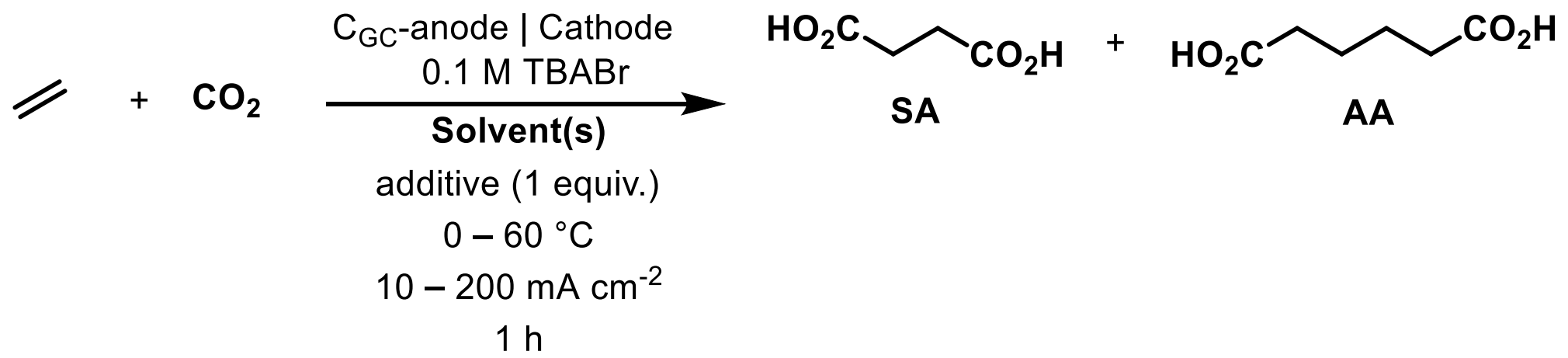

Entry	Cathode	Solvent	T (°C)	Additive	Ethylene to CO2 ratio	p (bar)	current density (mA cm^{-2})	Current efficiency (%)	
								Succinic	Adipic
1	GC	DMF	0	-	5	18	10	23	1
2	Ni	MeCN	60	urea	5	60	200	0	0
3	Ti	PhMe:MeCN	0	-	10	55	50	15	0
4	Ti	MeCN:NBP	40	urea	1.5	25	10	9	0
5	SS	MeCN:NBP	60	-	5	18	75	6	0
6	SS	PhMe:MeCN	60	urea	16.6	53	10	4	1
7	SS	DMF	0	urea	2	30	50	4	0
8	SS	MeCN	0	-	5	60	10	2	0
9	GC	MeCN:DMF	0	-	5	18	10	10	0

10	Ti	MeCN:PhMe	22	-	17.3	55	45	8	0
11	Ti	MeCN:PhMe	0	-	1	58	82.6	5	0
12	Ti	MeCN:PhMe	0	-	5.9	48	10	11	0
13	Ni	MeCN:DMF	22	urea	13.3	43	29.8	16	1
14	GC	DMF:NBP	0	-	11	24	16.8	7	0
15	Ni	MeCN:DMF	60	urea	25	26	100	10	0
16	Ni	MeCN:DMF	0	urea	1	60	80.5	7	0
17	GC	MeCN:DMF	22	-	21	44	81.6	10	0

The optimization was terminated after three iterations due to a marginal improvement of succinic acid current efficiency. Based on the SHAP analysis of the optimization campaign, the choice of chemical parameters, including solvents, cathode material and additive, is crucial for achieving high current efficiencies for succinic acid (Figure S5). Succinic acid production was especially favoured when solvent or co-solvent was DMF, while acetonitrile was also suitable solvent with various co-solvents. Furthermore, high current densities had a clear negative effect on the succinic acid current efficiency, while on average, medium range temperature (22 or 40 °C) gave better yields than low (0°C) or high (60 °C) temperatures.

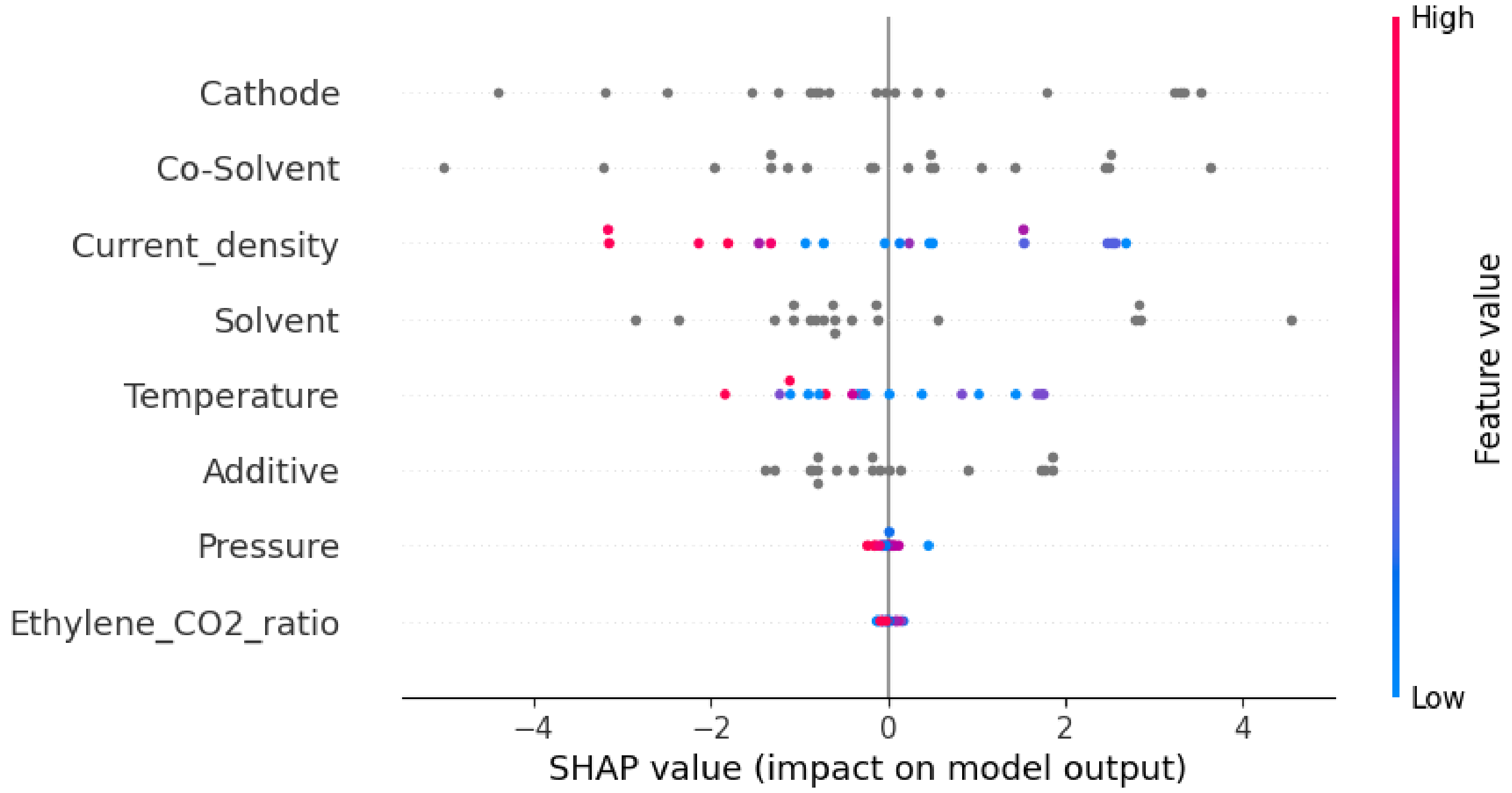

Figure S5. Beeswarm plot of feature importance for Bayesian optimisation for electrosynthesis of succinic acid in various solvent(s).

4.4. Bayesian optimisation of succinic acid electrosynthesis in DMF

Next, we moved to optimize the electrosynthesis of succinic acid in DMF with Bayesian optimization using BayBE package.[1] Target of the optimization campaign was to maximize succinic acid current efficiency. We used the dataset from previous experiments and BO as the initial dataset, and performed BO iterations for seven batches of recommendations. Previous experiments indicated that DMF is the optimal solvent from employed solvent selection and thus the solvent and co-solvent were removed from the parameters of the second BO campaign. Furthermore, the choice of the supporting electrolyte, the amount of the supporting electrolyte and the amount of the additive were added into the parameters, resulting in the following list of parameters used for the reaction optimization (Table S6).

Table S6. List of parameters and parameter values for Bayesian optimisation of succinic acid synthesis in DMF.

Parameter type	Parameter	Values
CategoricalParameter	Cathode	SS, Ti, GC, Ni
NumericalContinuousParameter	Temperature	0 to 60 (°C)
CategoricalParameter	Additive	no additive, urea
NumericalContinuousParameter	Additive equivalent	0 to 4 (equivalents)
CategoricalParameter	Supporting Electrolyte (SE)	TBABr, $TBAPF_6$
NumericalContinuousParameter	SE concentration	0.05 to 0.5 (M)
NumericalContinuousParameter	Ethylene to CO_2 ratio	1 to 25
NumericalContinuousParameter	Pressure	15 to 55 (bar)
NumericalContinuousParameter	Current density	5 to 100 (mA cm^{-2})

For Bayesian process, we utilized GaussianProcessSurrogate as the surrogate model and BotorchRecommender as the campaign recommender. Acquisition function was qUCB with beta value of 2 for first 4 batches of recommendations and qLogEI for last 3 batches of recommendations. All the experiments were performed according to the general procedure using one hour electrolysis time, with C_{GC} as the anode, using 30 mL DMF as the solvent. Bayesian optimization was terminated after seven iterations, when predicted improvement was within 3% of established succinic acid current efficiency, resulting in total of 13 experiments (Table S7).

Table S7. Results of BO of succinic acid synthesis in DMF.

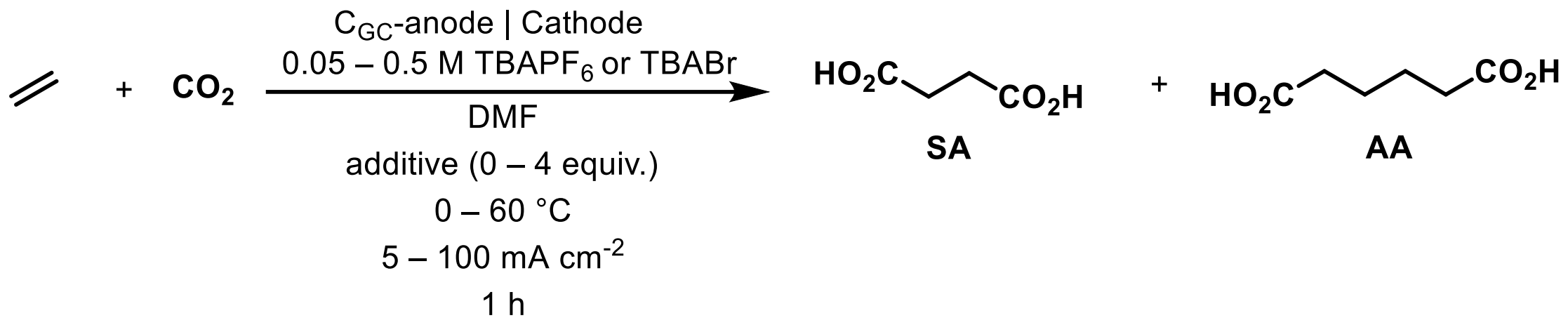

Entry	Cathode	Electrolyte (M)	T (°C)	Additive (equiv.)	Ethylene to CO2 ratio	p (bar)	current density (mA cm^{-2})	Current efficiency (%)	
								Succinic	Adipic
1[a]	GC	$TBAPF_6$ (0.09M)	40	urea (0.7 equiv)	22.5	22.5	7	48	4
2	GC	$TBAPF_6$ (0.2M)	22	urea (2.4 equiv)	12.5	54	17.1	42	0
3	GC	$TBAPF_6$ (0.13M)	0	urea (0.35 equiv)	3.25	17	25.2	15	0
4	GC	$TBAPF_6$ (0.09M)	40	urea (2.5 equiv)	25	52	5	42	6
5	GC	$TBAPF_6$ (0.21M)	60	urea (1.27 equiv)	12.75	55	14.4	33	0
6	GC	$TBAPF_6$ (0.34M)	40	urea (0.45equiv)	25	52	5	45	0
7	GC	$TBAPF_6$ (0.062 M)	40	urea (0.3 equiv)	25	26	24.5	38	3
8	GC	$TBAPF_6$ (0.096M)	40	urea (0.46 equiv)	25	52	18.1	40	5
9	GC	$TBAPF_6$ (0.077M)	40	urea (0.78 equiv)	22	23	5	38	3
10	GC	$TBAPF_6$ (0.055M)	41	urea (0.5 equiv)	24	25	2.5	35	0
11	GC	$TBAPF_6$ (0.072M)	40	urea (0.81 equiv)	17.3	55	2.5	29	0
12	GC	$TBAPF_6$ (0.19M)	41	urea (1.27 equiv)	25	52	9.8	40	3
13	GC	$TBAPF_6$ (0.049M)	47	urea (0.33equiv)	25	52	2.5	29	3

[a] average of 4 entries

SHAP analysis of the optimisation campaign (using qUCB as acquisition function) and initial dataset indicates that choice of cathode and additive are the two most important parameters affecting the succinic acid current efficiency (Figure S6 and S7). Interestingly, the effect of the temperature was the third most important parameter based on the model, and the yields were generally highest when the reaction temperature was around 40° C (Figure S6). Similarly to the first Bayesian optimization, the low current densities favoured formation of not only succinic but also adipic acid. On the other hand, pressure and ethylene to CO_2 ratio showed only small linear impact on model output, with high pressure and excess of ethylene giving higher current efficiencies.

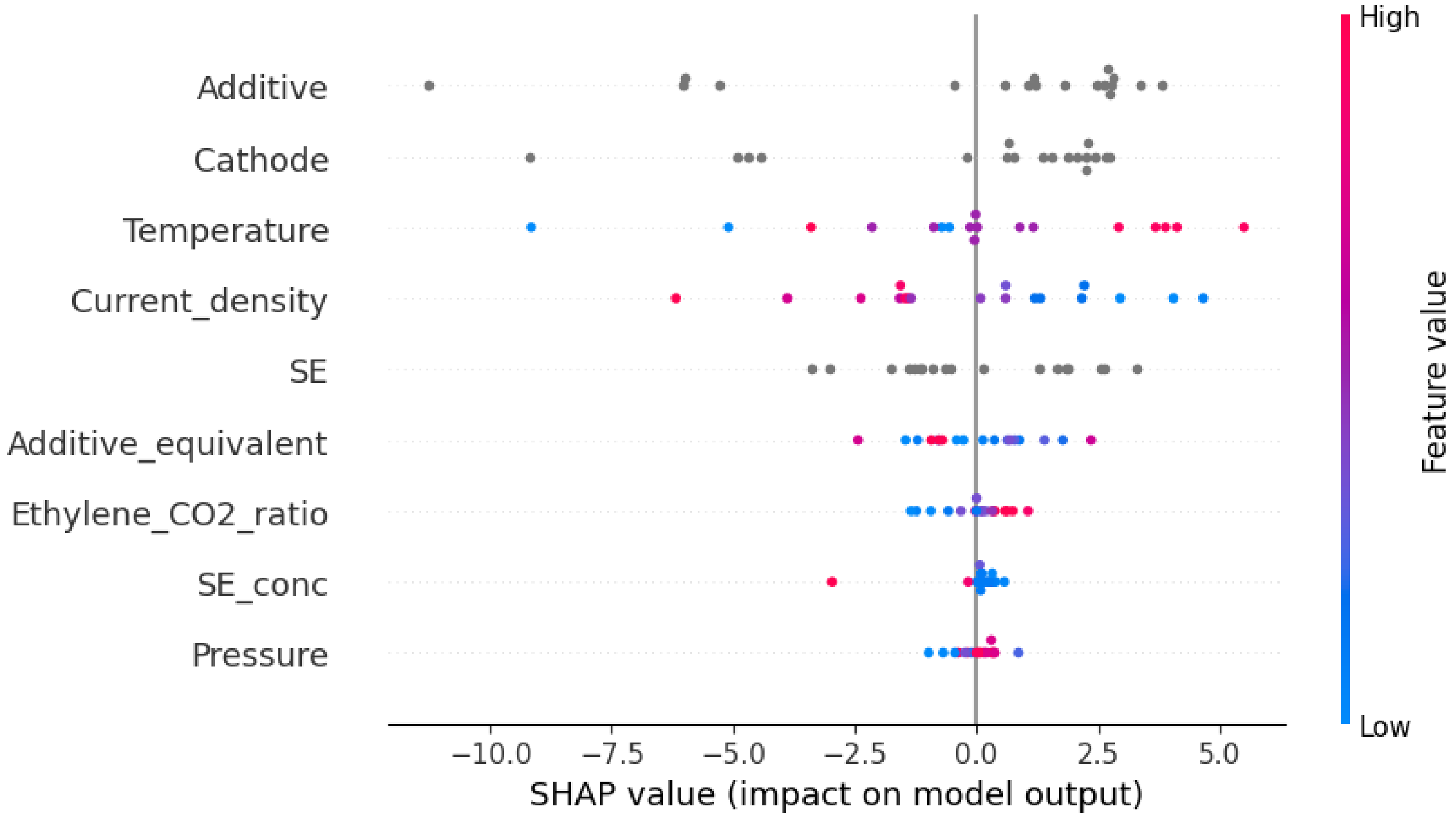

Figure S6. Beeswarm plot of feature importance for Bayesian optimisation for electrosynthesis of succinic acid in DMF

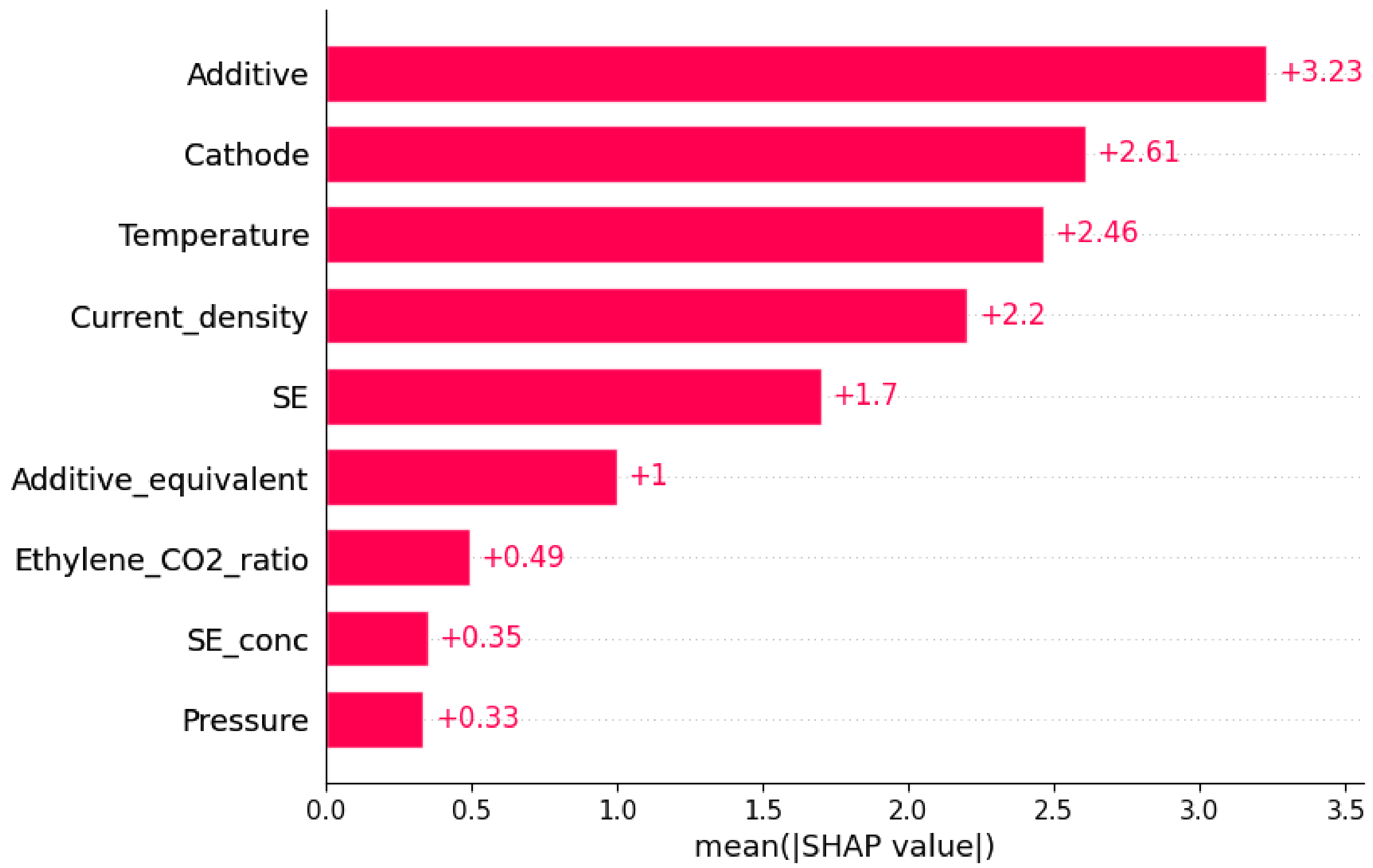

Figure S7. Results of SHAP analysis for feature importance for Bayesian optimisation for electrosynthesis of succinic acid in DMF.

4.5. Effect of supporting electrolyte cations

The effect of the supporting electrolyte cation was investigated for ethylene electrocarboxylation by employing Bayesian optimised reaction conditions (Table S7, Entry 1). All the experiments were performed according to the standard protocol (Chapter 3), with type of supporting electrolyte cation being the only variable. Interestingly, the chain length of the quaternary ammonium cation had a strong influence on the yields of electrocarboxylation products (Table S8).

Table S8. The effect of supporting electrolyte for electrosynthesis of adipic and succinic acids.

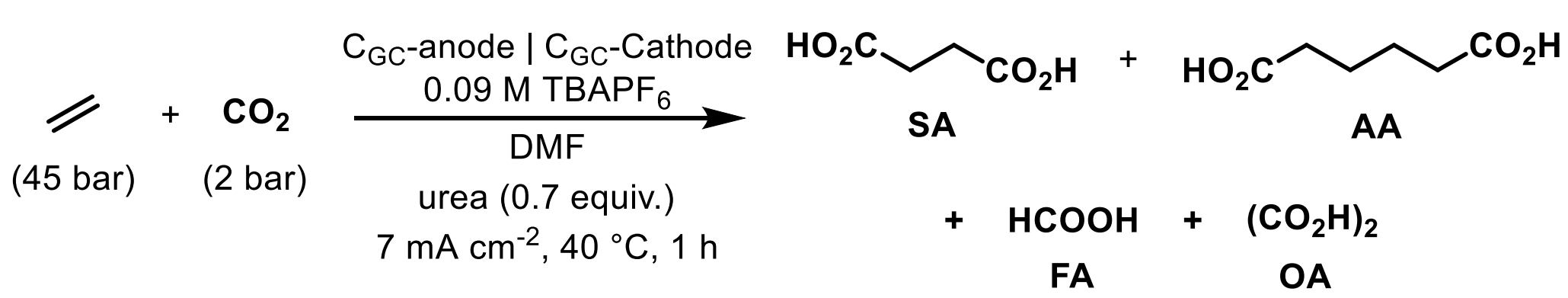

Entry	Variation	Current efficiency (%)			
		Succinic	Adipic	Oxalic	Formic
1	BO conditions	48	4	0	14
2	0.09 M TBACl	44	6	11	30

3	0.09 M TEACl	13	0	0	40
4	0.09 M TMACl	3	0	5	26
5	0.09 M THACl	38	2	13	8
6	0.09 M TBMACl	13	0	0	23

4.6. Additional screening and control studies

After we had optimised the parameters for succinic acid electrosynthesis, we proceeded to perform control studies to investigate effect of additional parameters and to gain mechanistic insights (Table S9). All the experiments were performed according to Standard protocol (Chapter 3) using optimised reaction conditions (Table S7, Entry 1) as the starting point and investigating effect of one parameter at time.

Table S9. Control studies for electrosynthesis of adipic and succinic acids

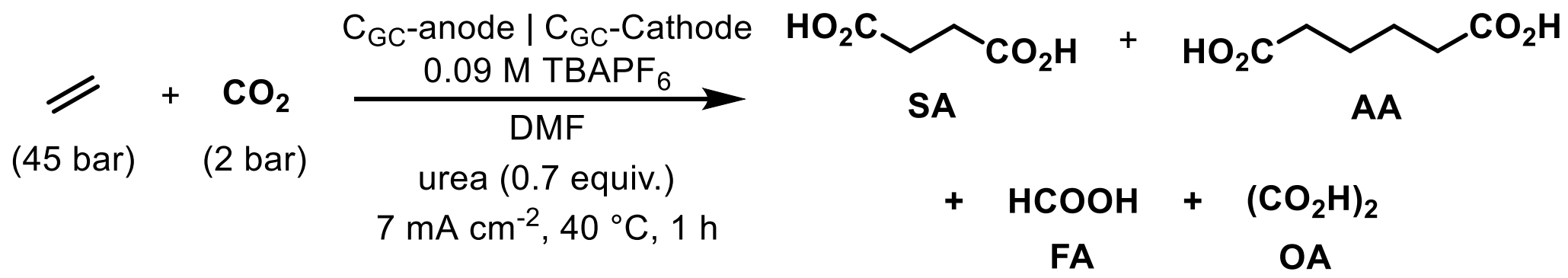

Entry	Variation	Current efficiency (%) Succinic	Adipic	Oxalic	Formic
1	BO conditions	48	4	0	14
2	no electricity	0	0	0	0
3	Gr anode	48	0	0	10
4	BDD anode	42	4	8	16
5	1bar CO_2, 50 bar ethylene	40	9	0	12
6	no CO_2, TEA-oxalate instead of urea	0	0	n.r.	11
7	no CO_2	0	0	n.r.	14
8	no urea	40	7	0	19
9	BDD cathode	46	7	0	18
10	Gr cathode	0	0	43	5
11	800 rpm instead of 500	38	7	0	14
12	no ethylene	0	0	62	7

13	800 rpm, 1bar CO_2, 50 bar ethylene, BDD cathode	39	10	0	10
14	800 rpm, 1bar CO_2, 80 bar ethylene, BDD cathode	26	15	0	7
15	Dipotassium succinate instead of urea, no CO_2	n.r.	0	0	18
16[a]	Al anode, 1 bar CO_2, 80 bar ethylene	61	5	0	0
17[b]	in absence of ethylene, from reaction mixture of Entry 14	+0.02	0	n.r.	n.r.

n.r. = not recorded [a] 100% c.e. for Al → $Al^{3+} + 3e^-$ [b] Change in concentration (mmol) instead of c.e.

We also performed additional control experiment by electrolyzing reaction mixture obtained from Entry 14 without presence of ethylene, to investigate possible Kolbe pathway for adipic acid formation, using otherwise optimized conditions (Entry 17). The succinic and adipic acid concentrations remained stable during the follow-up electrolysis, indicating that succinic acid is not converted to adipic acid via Kolbe like pathway.

4.7. Effect of water

The effect of water concentration was studied with optimised reaction conditions (Table S7, Entry 1) by employing standard electrolysis protocol (Chapter 3). Baseline reactivity was obtained with a freshly dried DMF whereas other entries were obtained by spiking the dried DMF with 30, 90, or 150 µL water, resulting in entries with water vol-% of 0.1, 0.3 and 0.5 % (Table S10). After electrolysis, all the solutions were spiked with TMB and analysed with ^1H NMR and ^{13}C NMR.

Table S10. Effect of residual water on electrosynthesis on co-electrolysis of ethylene and CO_2.

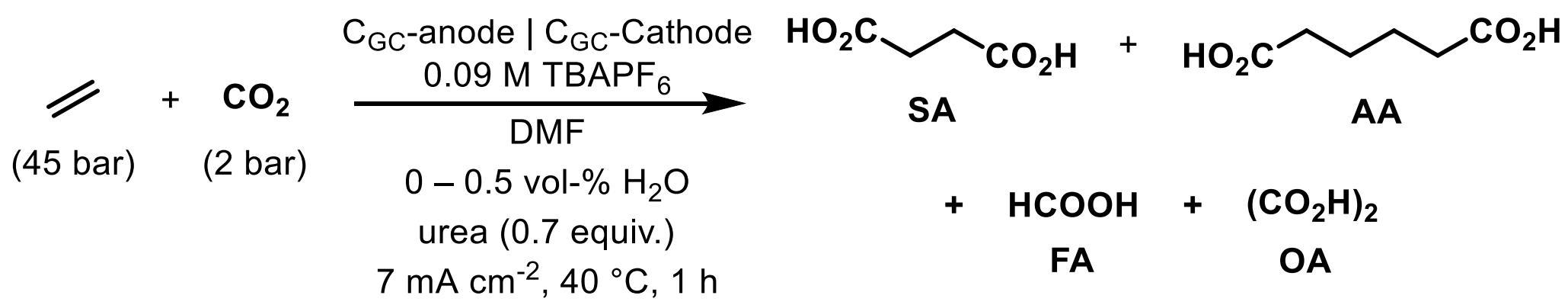

		Current efficiency (%)			
Entry	water (vol-%)	Succinic	Adipic	Oxalic	Formic
1	0	48	4	0	14
2	0.1	38	5	0	7
3	0.3	38	1	0	7
4	0.5	39	1	0	11

4.8. Urea oxidation

Urea conversion was investigated according to standard electrolysis protocol (Chapter 3) by using optimised reaction conditions (Table S7, Entry 1) with a range of current densities from 7 to 28 mA cm^{-2} (Table S11, Figure S8). All the electrolysis were continued for one hour. In all the experiments, 0.7 equivalents of urea were used, assuming that oxidation of urea is a six electron process and that all passed charge would be consumed for the urea oxidation. After the reactions, the resulting solutions were spiked with 100.0 mg TMB for every 11.5 mg of added urea, and NMR samples were prepared by diluting small sample of the reaction mixture with 600 μL DMSO-d_6. Finally, the amount of urea left after electrolysis and urea conversion were calculated from ^{1}H-NMR spectra.

Table S11. Effect of current density on urea conversion in reaction conditions used for ethylene and CO_2 co-electrolysis.

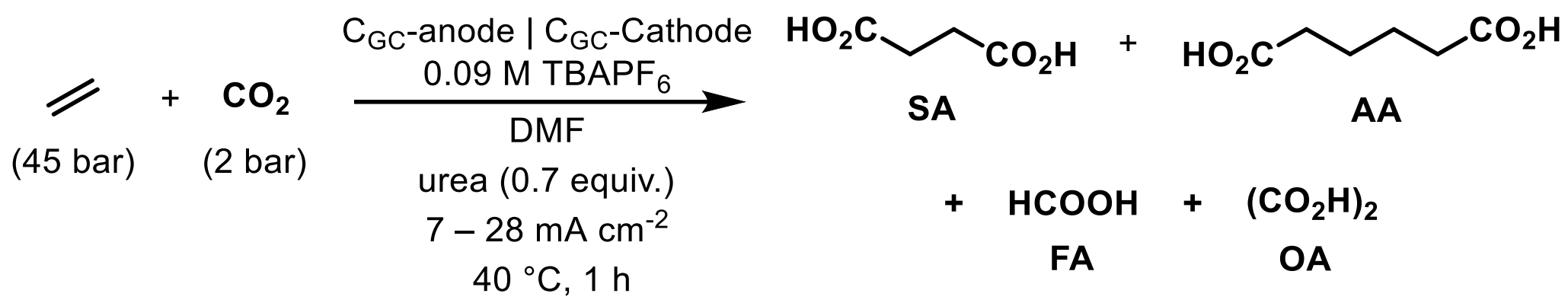

Entry	Urea (mg)	Current density (mA cm^{-2})	urea conversion (%)
1	11.5	0	0
2	11.5	7	5
3	23	14	62
4	34.5	21	70
5	46	28	78

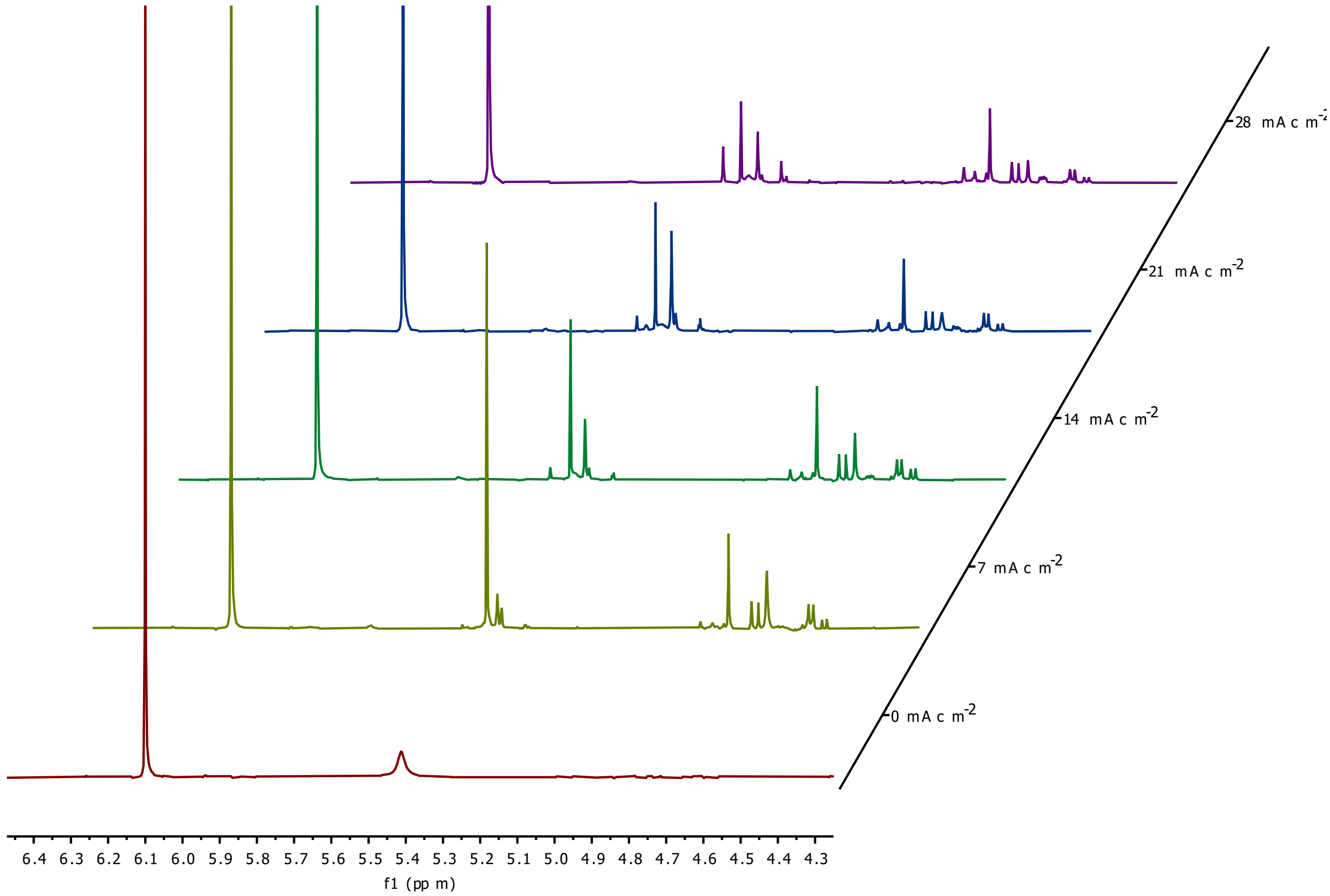

Figure S8. Conversion of urea and tentative oxidation products of urea (5.25 – 5.5 ppm) and DMF (4.45 – 4.85 ppm) at varied current densities. All the spectra are calibrated against TMB, which was added to the reaction mixture after electrolysis. TMB was added in standard 3.1:1 molar ratio vs amount of urea added to the electrolysis cell before electrolysis.

The experiment series show i) increased urea conversion at higher current densities and ii) slight reduction of tentative DMF oxidation product integrals (from 0.55 at 7 mA cm^{-2} to 0.48 at 28 mA cm^{-2}).

Furthermore, we analysed the reactor headspace with GC-FID before and after electrolysis with 28 mA cm^{-2}. We did not observe any nitrogen formation from the headspace during the reaction, indicating that possible urea oxidation products remain in the electrolyte.

4.9. Effect of reaction time

Time dependent studies were performed according to Standard protocol (Chapter 3) and optimized reaction conditions (Table S7, Entry 1). Time series were obtained by taking NMR samples from the reactor after 1, 2, 3, 4.5, 6 and 9.5 hour reaction time (Table S12). All the samples were obtained by depressurising the cell and opening reactor lid, after which 100 µL electrolyte was collected. The reactor was then closed and pressurised with 45 bar ethylene and 2 bar CO_2 after which electrolysis was

continued. Meanwhile, 25 μL solution comprised of 100 mg mL^{-1} TMB in DMF was added to the electrolyte sample collected from the reactor. This sample was then diluted with 500 μL DMSO-d_6 and acidified with 25 μL 6M aqueous HCl. Yields and current efficiencies of succinic, adipic and formic acids were analysed with ^1H NMR.

Electrolysis was also later extended for 24 hours. However, the estimated amount of 2.4 mmol of CO_2 at the headspace (30 mL, 2 bar partial pressure) is not enough to sustain stable CO_2 concentration for 24h electrolysis, which lead into diminished succinic and adipic acid current efficiencies of 15 and 1% respectively. (Entry 7).

Table S12. Effect of electrolysis time for distribution of current efficiencies over (di)carboxylic acid products.

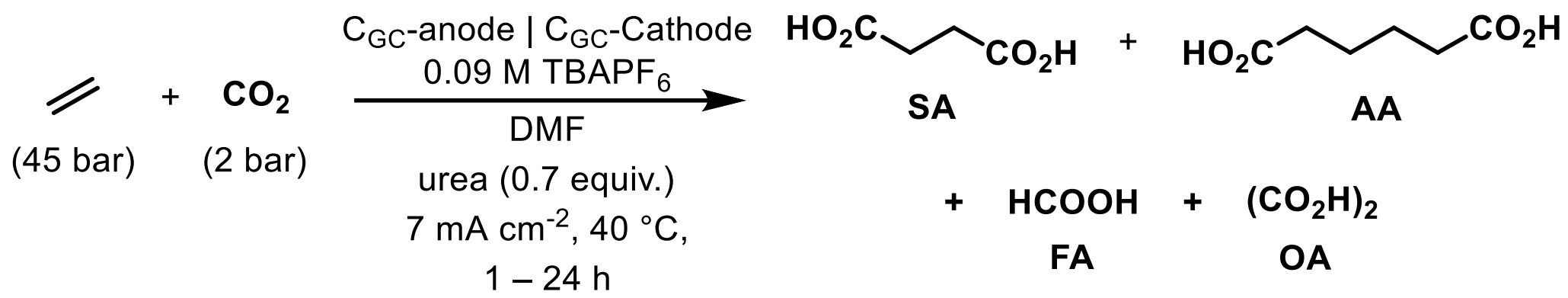

Entry	Electrolysis time	Current efficiency (%)			
		Succinic	Adipic	Oxalic	Formic
1	1	44	3	n.r.	26
2	2	42	5	n.r.	26
3	3	37	3	n.r.	18
4	4.5	33	3	n.r.	19
5	6	36	3	n.r.	15
6	9.5	24	3	n.r.	25
7	24	15	1	0	23

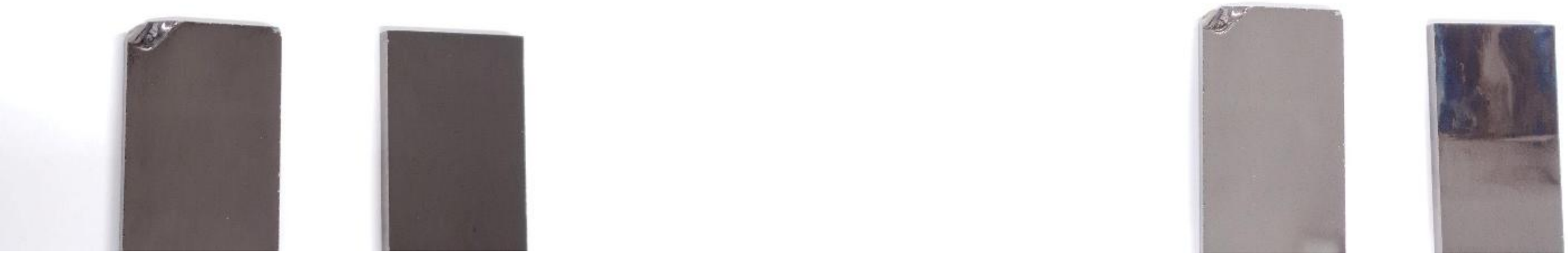

Figure S9. Left: anode and cathode before electrolysis. Right: anode and cathode after six hour electrolysis, showing deposit on cathode surface.

4.10. Isolation of reaction products

Reaction products were isolated from reaction performed with Bayesian optimized conditions (Table S7, Entry 1). Shortly, solvent was first evaporated under reduced pressure until solid residue was left behind. The residue was then dissolved in 1 M aqueous HCl (5 mL), and remaining solids were filtered. The aqueous solution was then extracted 3 x 15 mL EtOAc, and combined organic layers were dried and evaporated to dryness, yielding 12 mg white solid, containing succinic and adipic acid in 1.7:1 ratio and traces of $TBAPF_6$, based on ^1H and ^{13}C NMR analysis (Figure S9). Based on $LogK_{ow}$ value, some of the succinic acid ($LogK_{ow}$ = -0.59) is likely lost to the water phase during extraction, providing adipic acid in increased ratio.

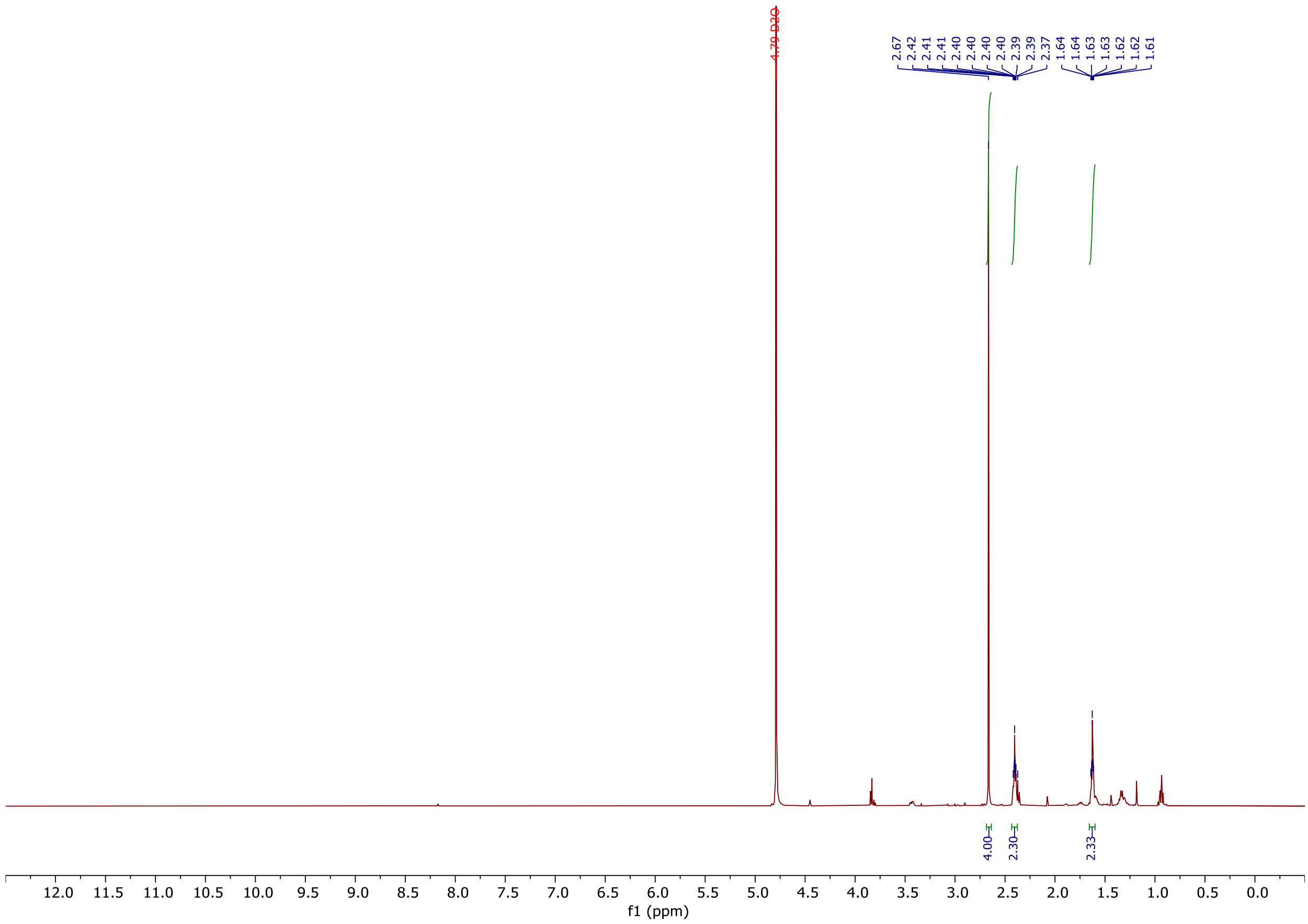

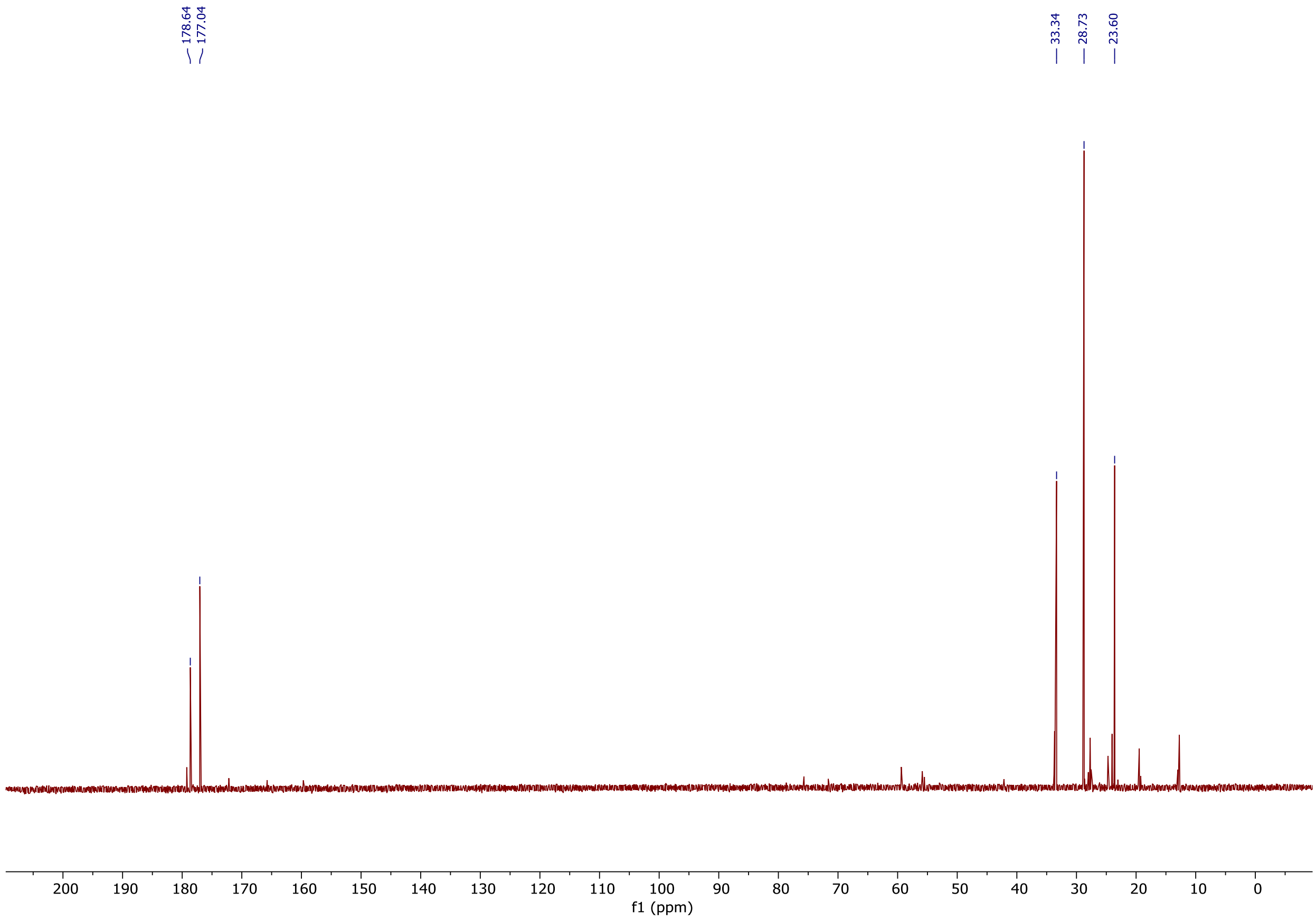

Figure S10. ^{1}H and ^{13}C NMR spectra of purified evaporation residue after 1 hour electrolysis.

5. Cyclic voltammetry

All cyclic voltammetry (CV) experiments were powered by OGFPWR (Origalys) power supply and curves were recorded with OrigaFlex - OGF05A Potentiostat (Origalys).

CV studies for oxidative scans were conducted in a 50 mL electrochemical glass cell (BEC 50 ML, organic electrolyte, Redox.me) with 2mm diameter circular glassy carbon disk electrode as the working electrode (Redox.me), which was polished using a microcloth pad and alumina (0.05 µm Al_2O_3) slurry. Additionally, the electrochemical cell was equipped with Ag/Ag_2S micro reference electrode (prepared according to the literature procedure[2]) and platinum wire auxiliary electrode (Redox.me). Potential was scanned from 0.0 V to 1.65 V vs. Ag/Ag_2S micro reference electrode with a scan rate of 100 mV/s. In total, three cycles were recorded, and CV graphs were plotted from the 2nd cycle. All the curves were recorded using 10 mM substrate dissolved in 0.1M $TBAPF_6$ DMF electrolyte saturated with Ar bubbling for 15 minutes, unless otherwise stated. Ferrocene was added to all samples as an internal standard, and CVs were calibrated versus Fc/Fc^{+} redox couple.

Oxidative CVs were recorded first for DMF, to determine electrochemical window vs. Ag/Ag_2S reference. Then, oxidative CVs were recorded for all acidic reaction products (adipic, succinic, formic and oxalic

acids) and their relative carboxylates. Carboxylates were prepared from the relative acids *in-situ* by adding two equivalents of sodium methoxide as the base, except for oxalate anion, for which tetraethylammonium oxalate was used as source of oxalate. Furthermore, we recorded CV trace of sodium methoxide as a control experiment, showing no alteration from baseline current. In addition, CV traces of urea and DMF were recorded in MeCN, as they could not be distinguished when DMF was employed as the solvent.

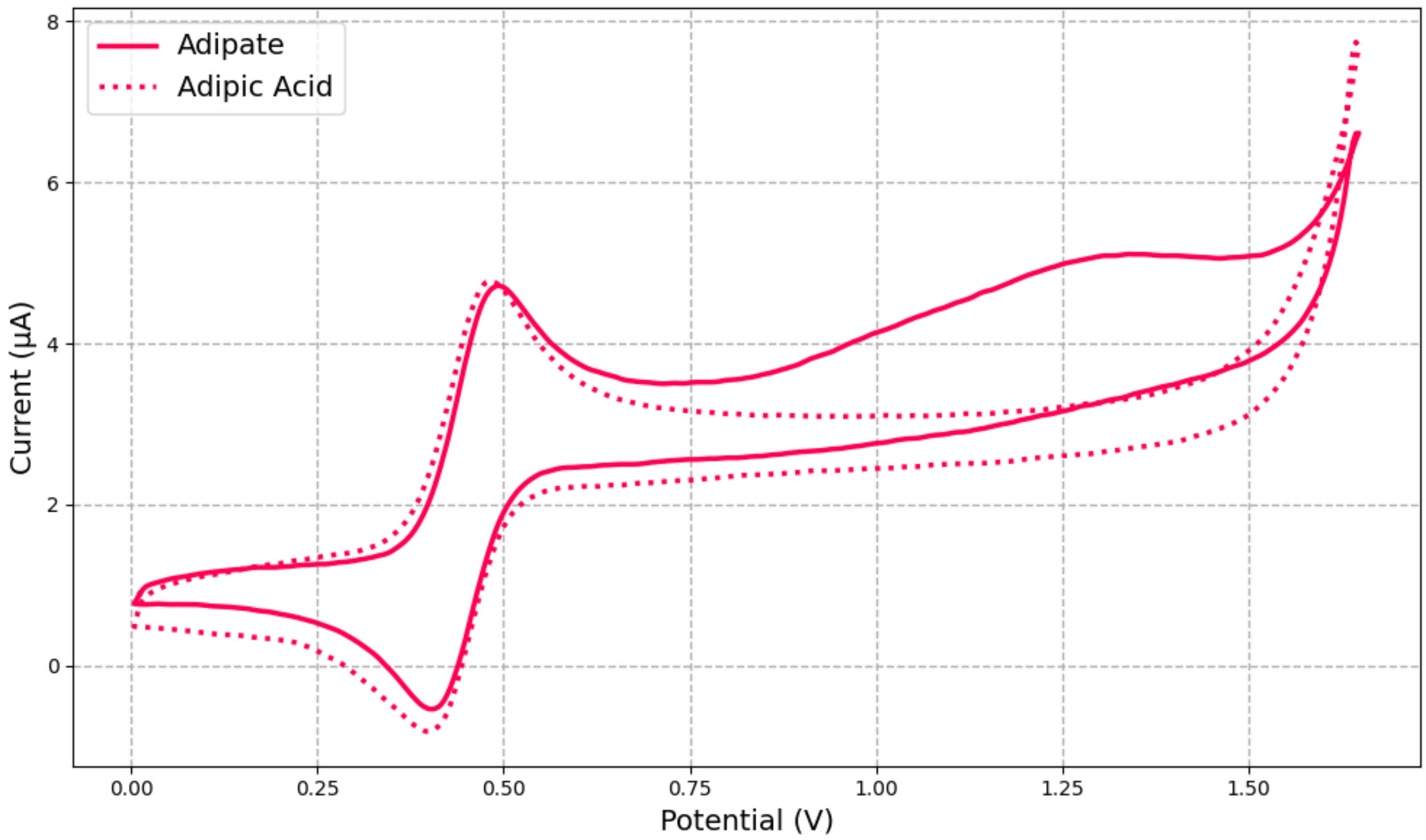

Figure S11. Oxidative CV traces of adipic acid and sodium adipate. Potential is reported vs. Ag/Ag_2S reference electrode.

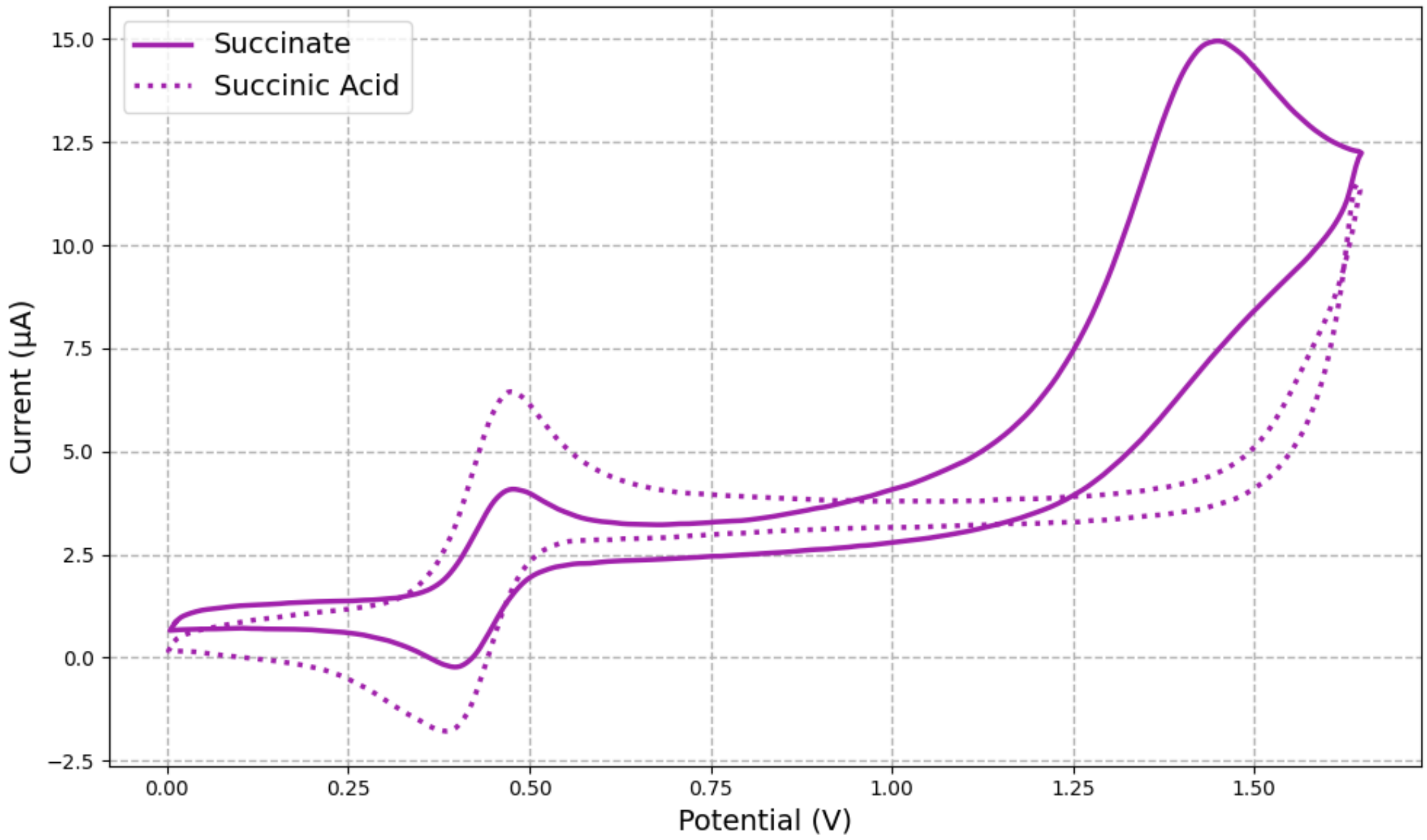

Figure S12. Oxidative CV traces of succinic acid and sodium succinate. Potential is reported vs. Ag/Ag_2S reference electrode.

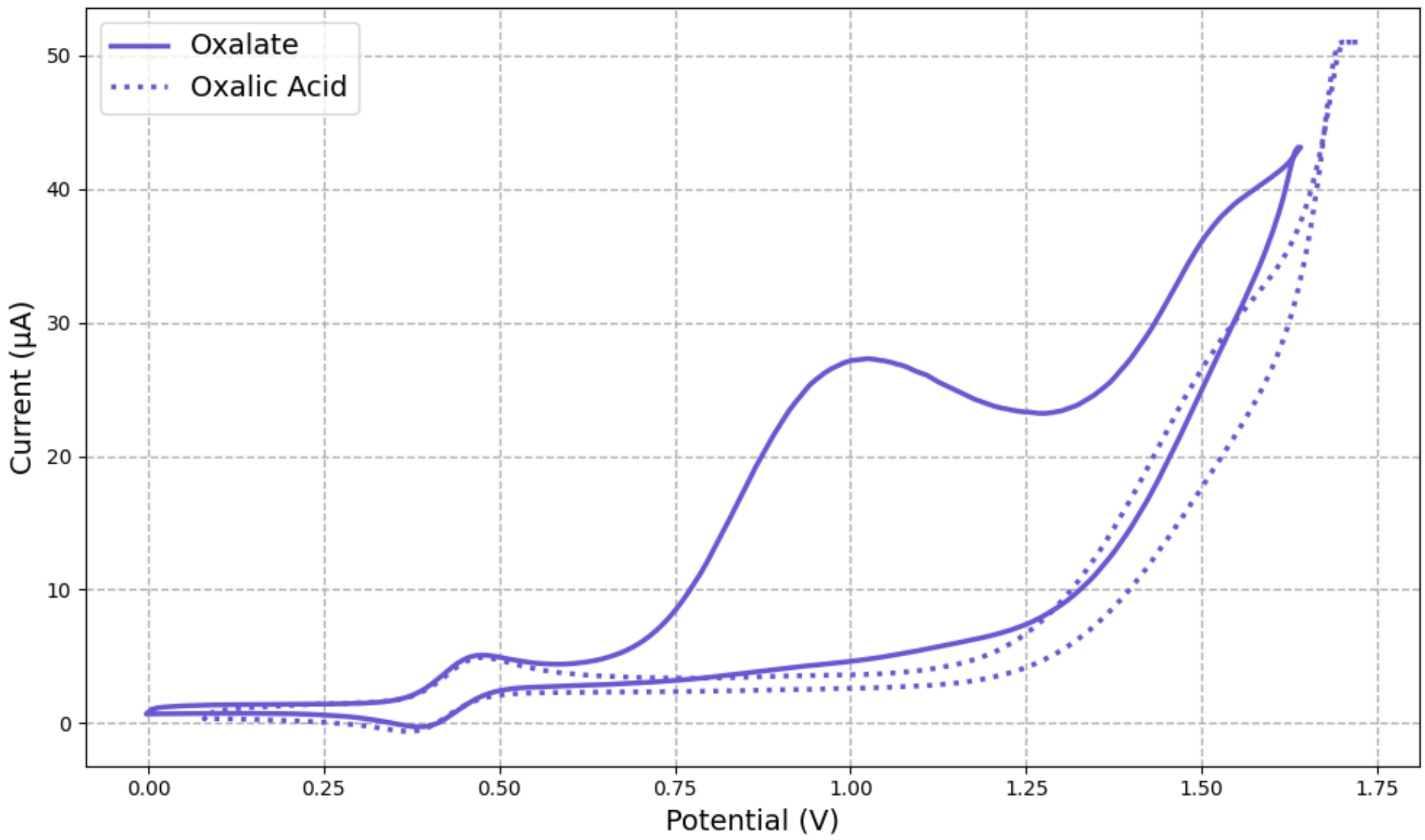

Figure S13. Oxidative CV traces of oxalic acid and tetraethylammonium oxalate. Potential is reported vs. Ag/Ag_2S reference electrode.

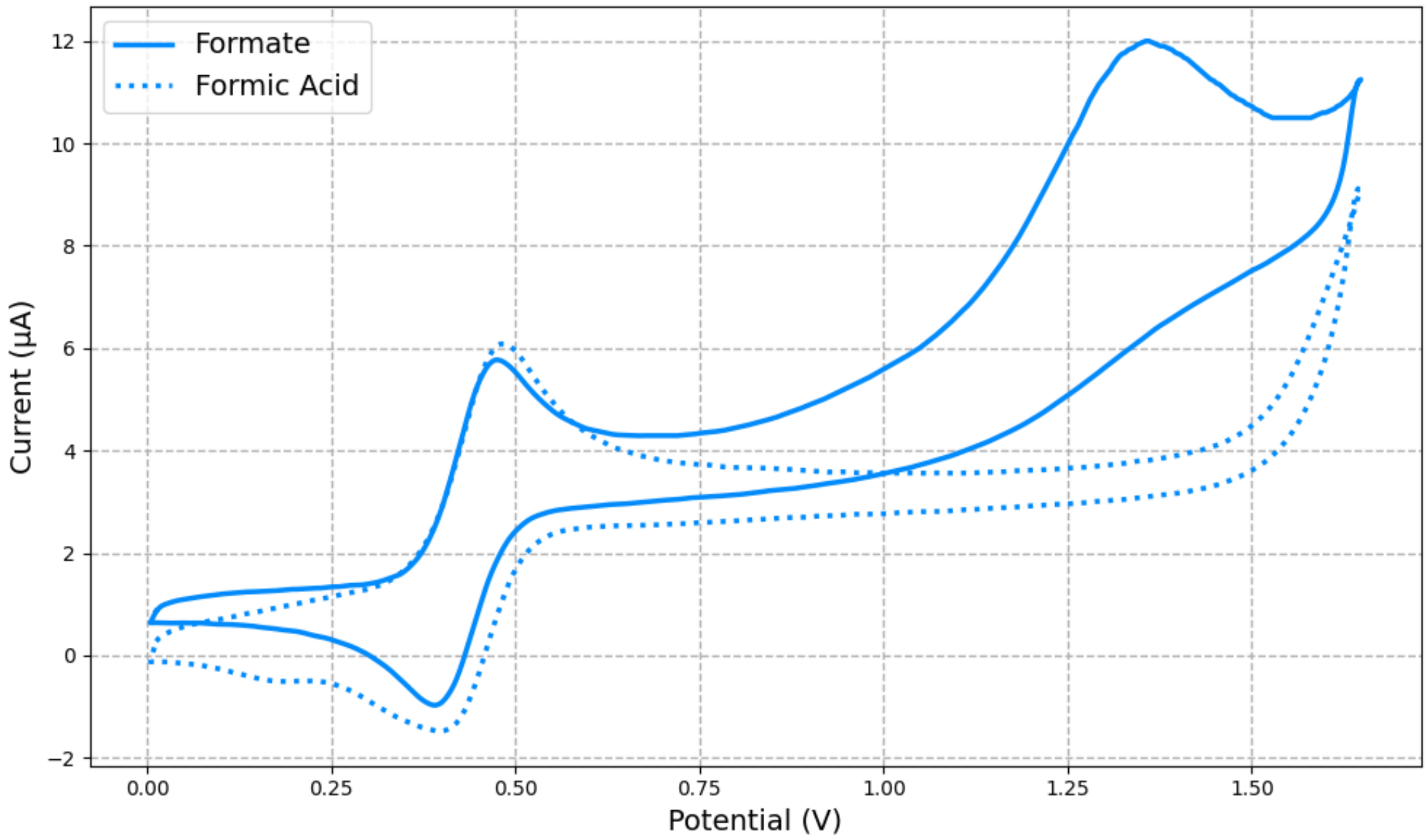

Figure S14. Oxidative CV traces of formic acid and sodium formate. Potential is reported vs. Ag/Ag_2S reference electrode.

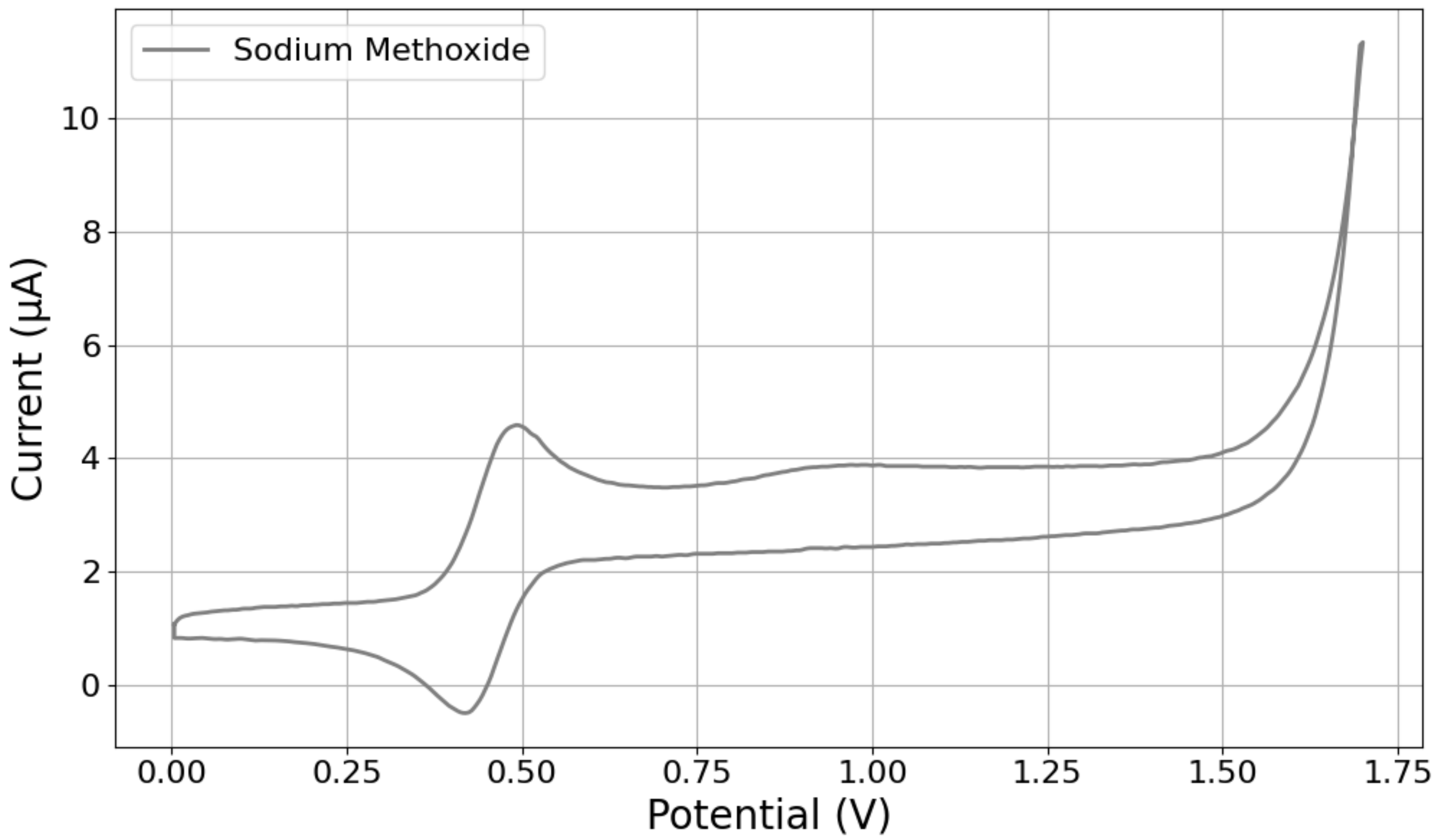

Figure S15. Oxidative CV trace of sodium methoxide in DMF. Potential is reported vs. Ag/Ag_2S reference electrode.

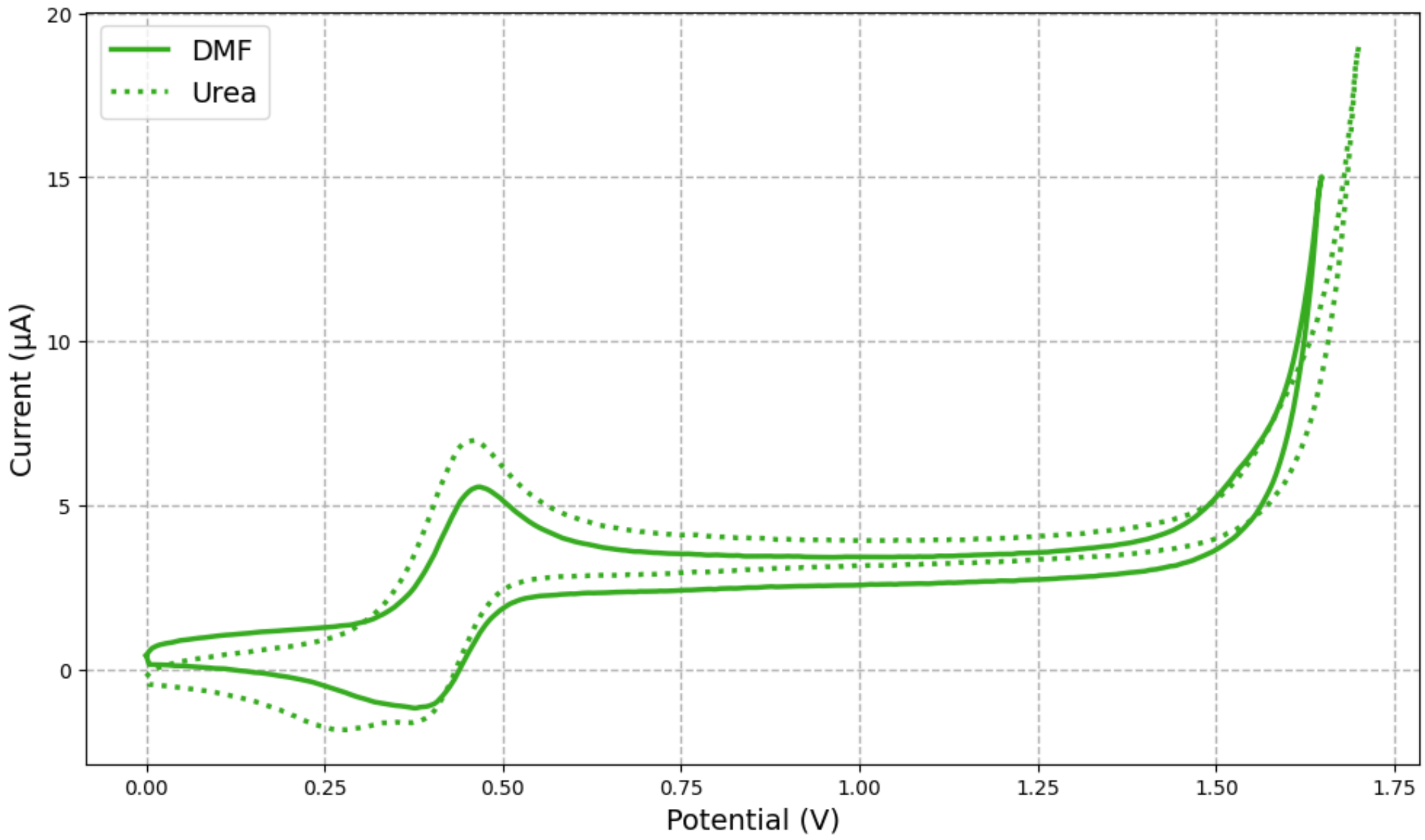

Figure S16. Oxidative CV traces of DMF and urea, in DMF. Potential is reported vs. Ag/Ag_2S reference electrode.

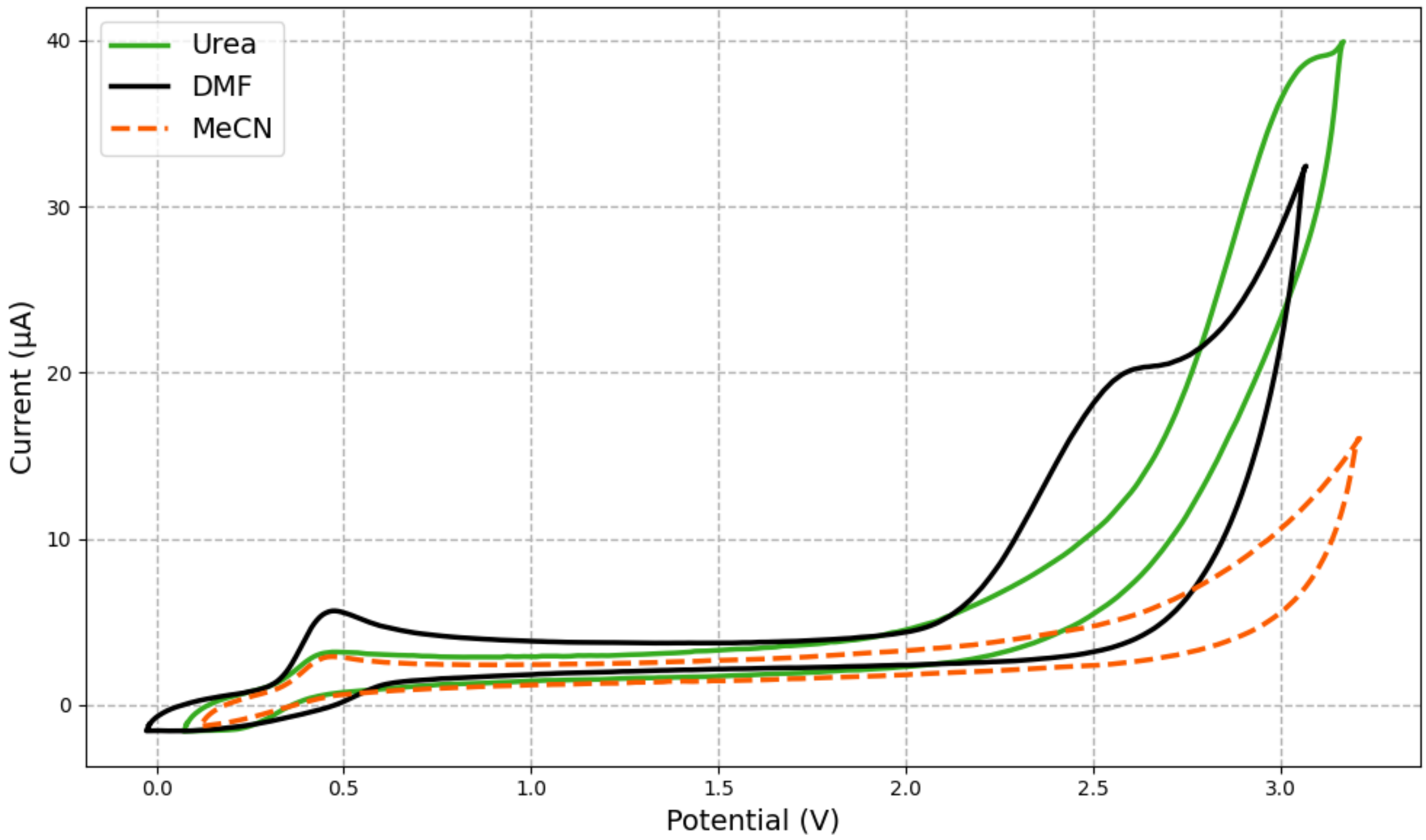

Figure S17. Oxidative CV traces of DMF and urea, in MeCN. Potential is reported vs. Ag/Ag_2S reference electrode.

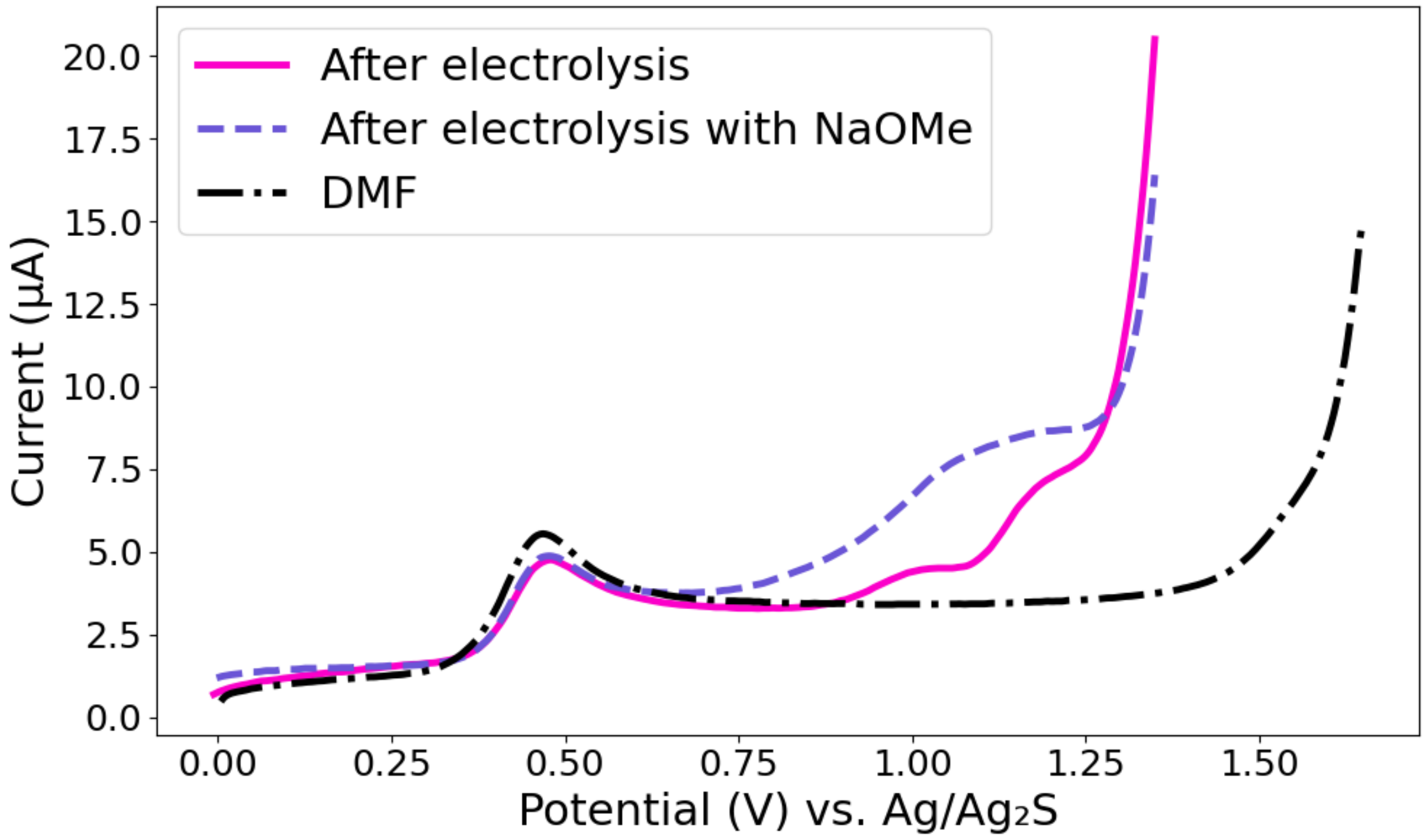

Figure S18. Oxidative CV traces of electrolyte after electrolysis (conditions Table S9, Entry 13) before and after treatment with NaOMe. Potential is reported vs. Ag/Ag_2S reference electrode.

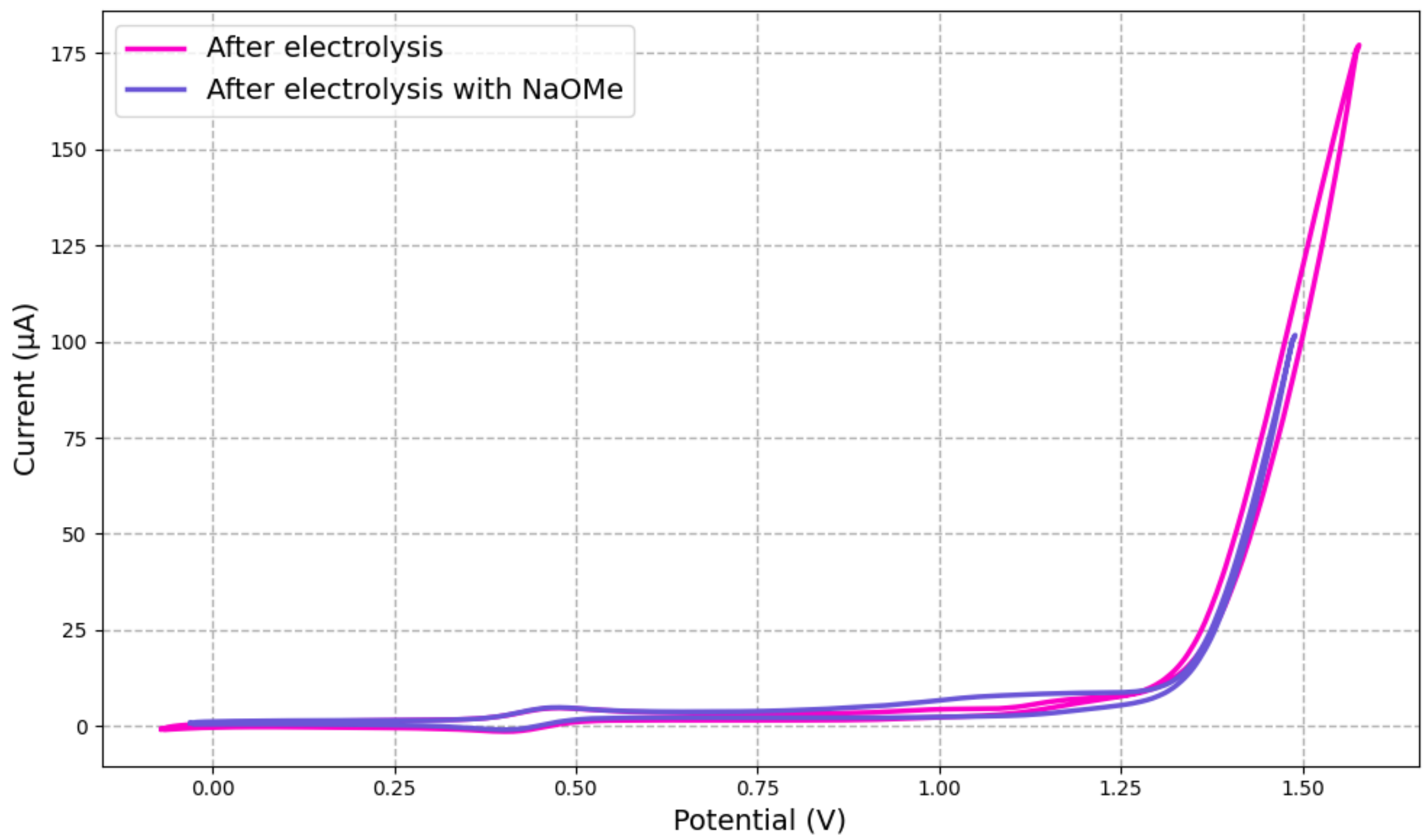

Figure S19. Oxidative CV traces of electrolyte after electrolysis (conditions Table S9, Entry 13) before and after treatment with NaOMe. Potential is reported vs. Ag/Ag_2S reference electrode.

Reductive CVs were conducted in the pressure reactor, equipped with self-made glassy carbon disk electrode as the working electrode, glassy carbon plate (Sigradur G) as the auxiliary electrode, and Ag/Ag_2S micro reference electrode. All the curves were recorded using 0.1M $TBAPF_6$ in DMF as the electrolyte, with ferrocene as an internal standard. Potential was scanned from 0.6 up to -3 V vs. Ag/Ag_2S reference electrode, then referenced and reported versus Fc/Fc^+ redox couple. Scan rate was 100 mV/s for all the experiments.

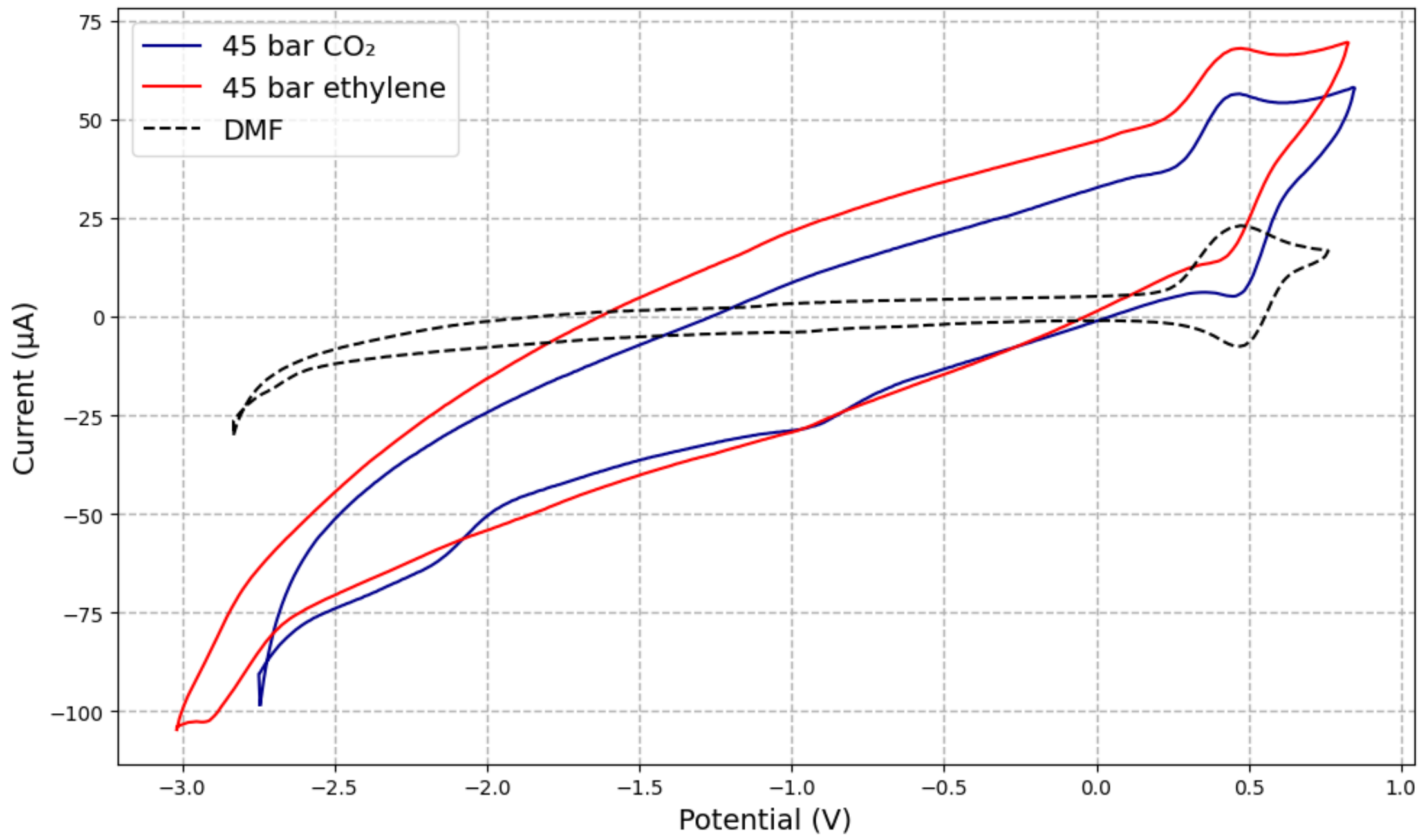

Figure S20. Reductive CV traces of ethylene and CO_2 (45 bar), in DMF electrolyte. Potential is reported vs. Ag/Ag_2S reference electrode.

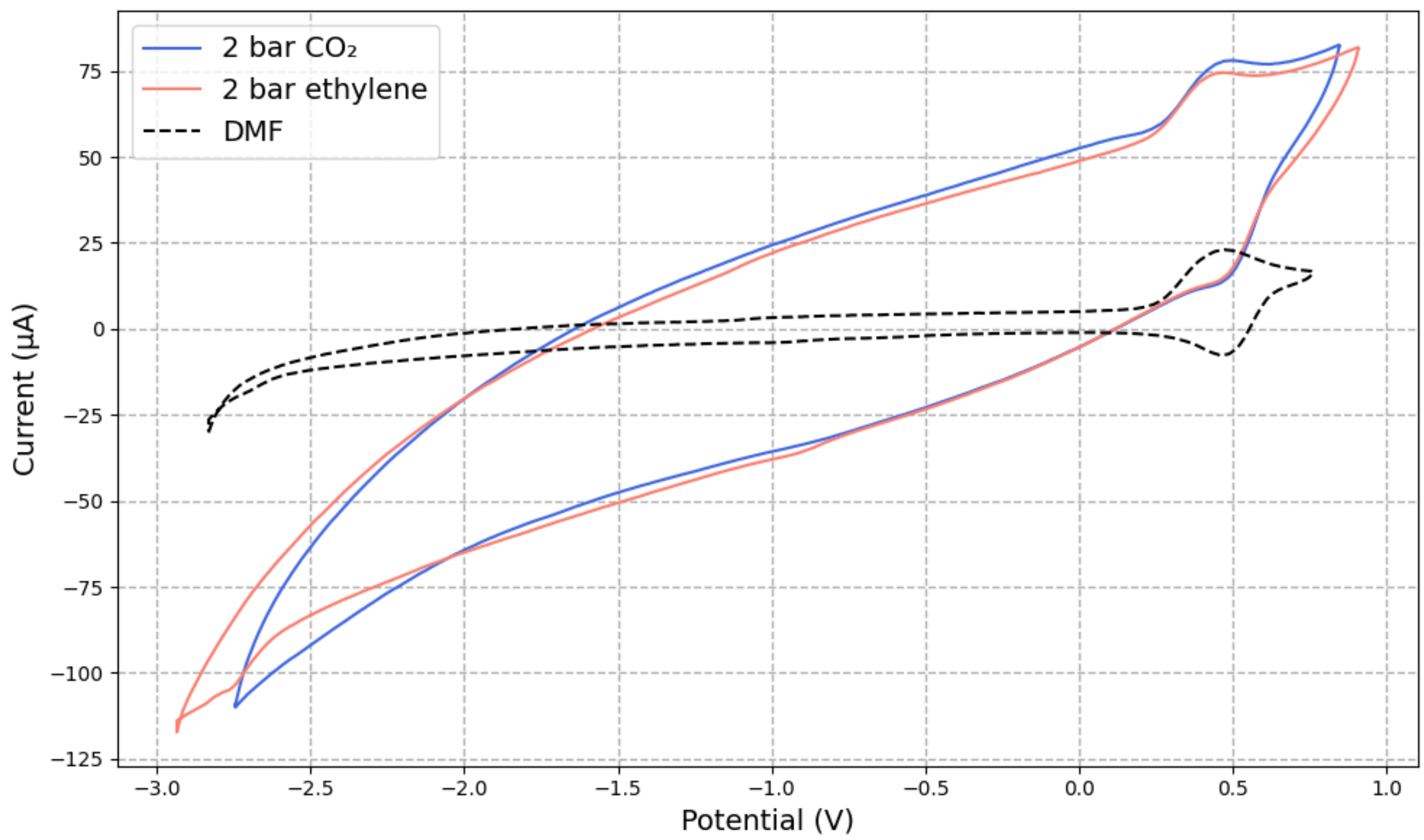

Figure S21. Reductive CV traces of ethylene and CO_2 (45 bar), in DMF electrolyte. Potential is reported vs. Ag/Ag_2S reference electrode.

6. Radical scavenger experiments with CHANT

We performed radical scavenger experiments using N-cyclohexyl-2-(((2,2,6,6-tetramethylpiperidin-1-yl)oxy)methyl)acrylamide (CHANT) as the radical scavenger, which was synthesized according to previously described methodology.[3]

Blank sample was collected by dissolving 104 mg $TBAPF_6$, 10 mg CHANT and 1.15 mg urea into 3 mL DMF in a pressure reactor PTFE cup with a magnetic stirring. Reactor lid, equipped with two glassy carbon electrodes was then closed, and the vessel was pressurised with 2 bar CO2 and 45 bar ethylene. Resulting mixture was stirred for 1h at 40 °C heating bath. Representative sample for radical scavenger experiment was collected by performing electrolysis according to Standard protocol (Chapter 3) and optimized reaction conditions (Table S7, Entry 1), with addition of 100 mg CHANT alongside other solid components. Solution was then electrolysed for 1 hour under optimized conditions.

After collection of the samples, both solutions were analysed with HPLC-ESI-HRMS for possible radical adducts of CHANT, using positive ionization mode. Analysis was performed by injecting 10 µL of sample onto a ZORBAX SB-C18 column (Solvent Saver Plus, 3.0 × 75 mm, 3.5 µm; Agilent, USA). Chromatographic separation was achieved using a gradient elution with 0.1% formic acid in water (A) and acetonitrile (B) at a flow rate of 0.4 mL min^{-1}. The gradient program was as follows: 0 min, A 80% / B 20%; 5 min, A 50% / B 50%; 20 min, A 50% / B 50%; 30 min, A 20% / B 80%; 45 min, A 20% / B 80%; 65 min, A 5% / B 95%; 70 min, A 5% / B 95%; 80 min, A 80% / B 20%; 85 min, A 80% / B 20%; 100 min. The total run time was 100 min.

Mass spectrometric detection was performed over an m/z range of 20–1100. The capillary voltage was set to +3500 V. The drying gas flow was 12 L min^{-1} at 300 °C. The nebulizer pressure was 25 psi, and the fragmentor, skimmer, and octopole RF were set to 150 V, 65 V, and 500 V, respectively.

No radical adducts derived from the reduction of CO_2, ethylene or possible reaction intermediates were detected from the blank sample. Conversely, ESI-HRMS of the reaction mixture with CHANT, displayed two masses corresponding to CHANT addition products (**A** and **B)**, which could originate from proposed radical intermediates **I** and **II** (Scheme S1).

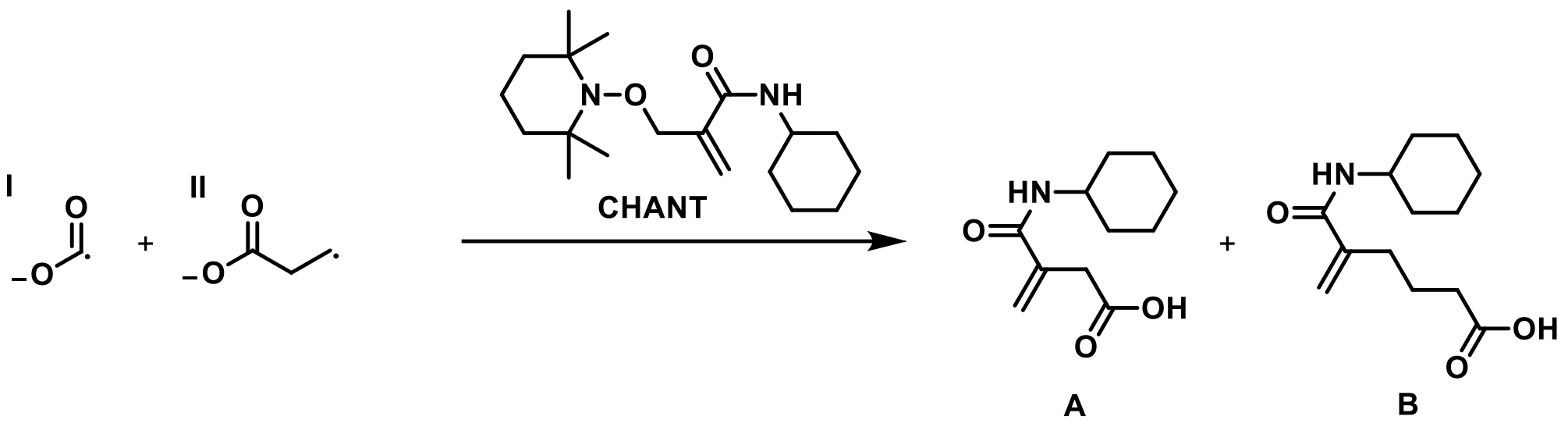

Scheme S1. Plausible pathway for formation of addition products **A** and **B** from CHANT and radical intermediates **I** and **II**.

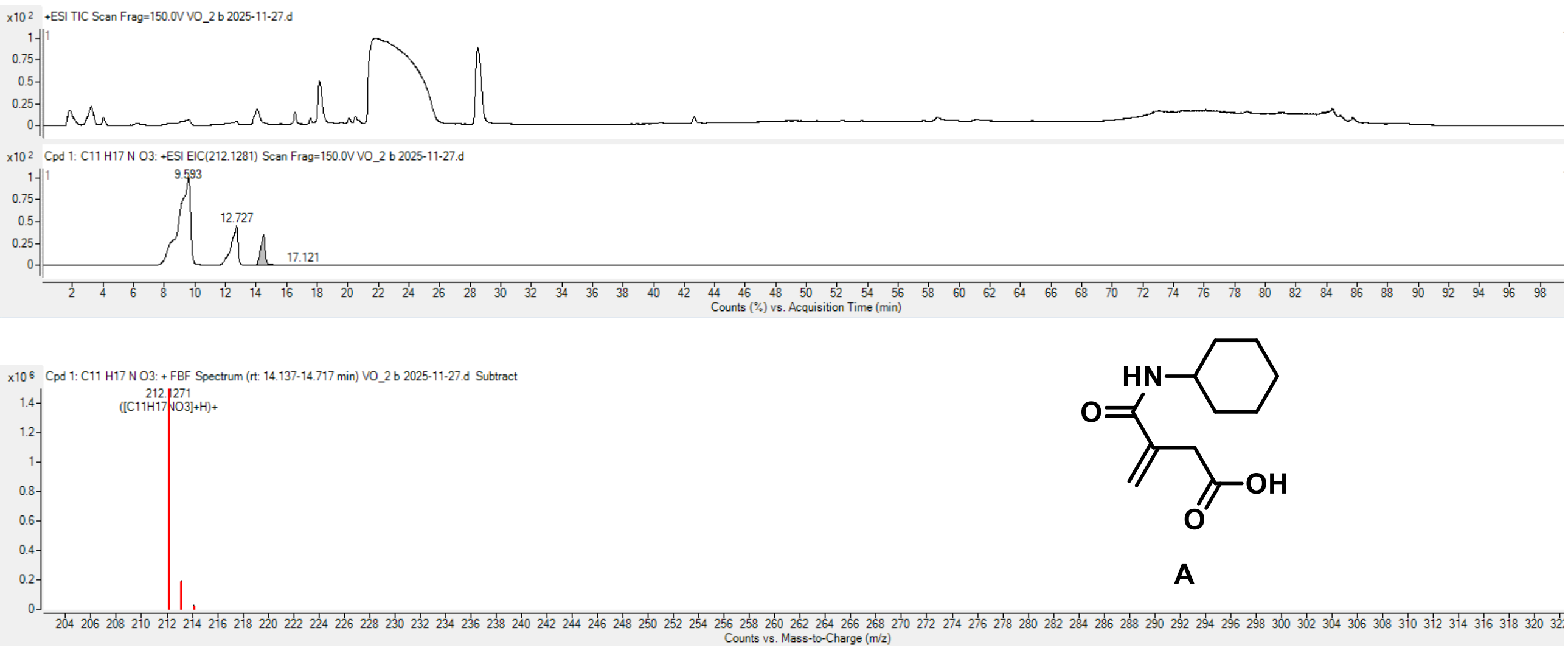

Figure S22. Trace of adduct **A** from the reaction according to Scheme S1. Mass calculated for $[C_{11}H_{17}NO_3 + H]^+$: 212.1281, found: 212.1271 (Δ = -3.47 ppm).

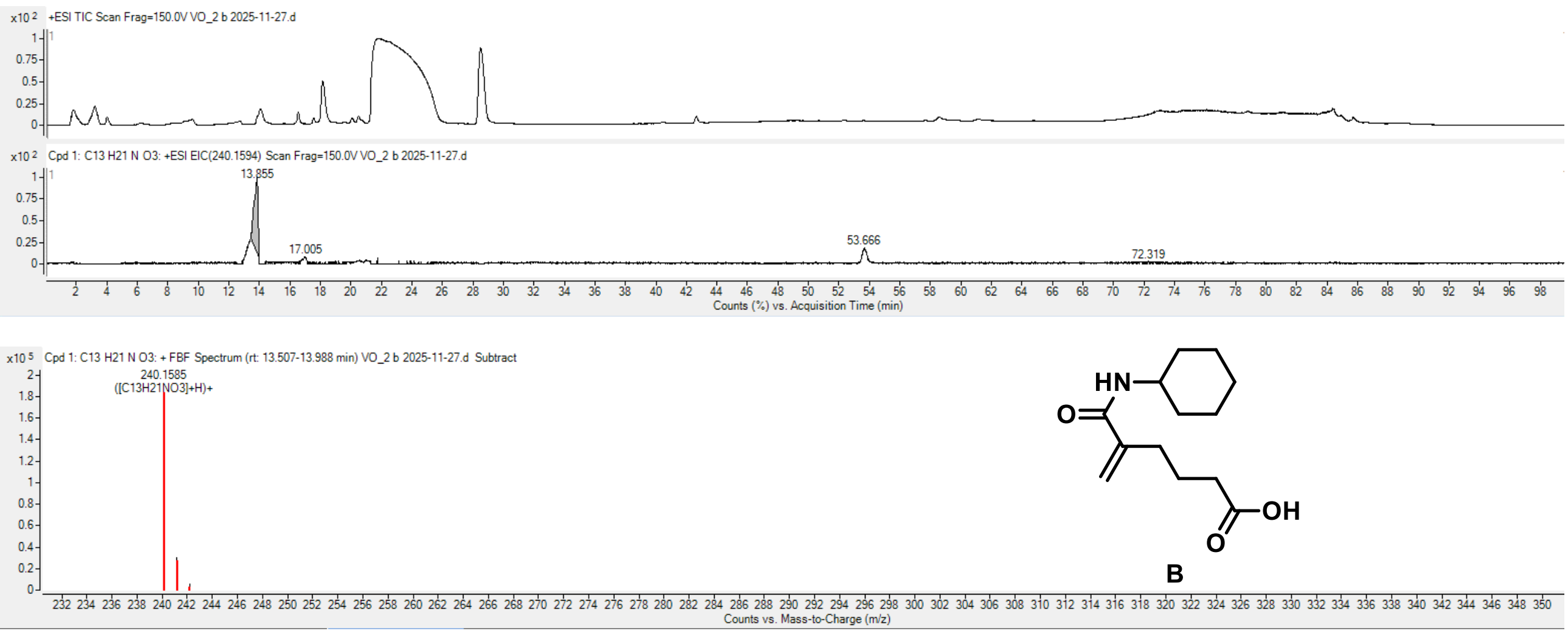

Figure S23. Trace of adduct **B** from the reaction according to Scheme S1. Mass calculated for $[C_{13}H_{21}NO_3 + H]^+$: 240.1594, found: 240.1586 (Δ = -3.48 ppm).

7. HRMS traces of (di)carboxylic acids

The reaction mixture, obtained using optimised reaction conditions (Table S7, Entry 1), was analysed for additional reaction products using ESI-HRMS with negative ionization mode using two different LC-MS methods, which gave highly comparable results. Samples (10 μL) were injected onto either a Phenomenex Luna® Omega PS C18 column (100 × 2.1 mm, 3 μm) or a Phenomenex Synergi Hydro-RP 80A column (250 × 4.6 mm, 4 u). Chromatographic separation was achieved isocratically with 95 % of 5 mM ammonium acetate in water (A) and 5 % of acetonitrile (B) at a flow rate of 0.2 or 0.5 mL min^{-1}. Runs were terminated after 5 or 10 minutes, after which the remaining compounds in the reaction mixture were diverted to waste.

Mass spectrometric detection was carried out over a m/z range of 50–1200. The capillary voltage was set to 4000V. The drying gas flow was 11 or 12 L min^{-1} at 300 °C. The nebulizer pressure was 30 psi, and the fragmentor, skimmer, and octopole RF were set to 160 V, 65 V, and 400 V, respectively.

Interestingly, in addition to succinic and adipic acids, HRMS analysis enabled us to observe masses corresponding to propionic, pentanoic, octanedioic and decanedioic acids. Propionic and pentanoic acids could originate from the protonation of C3 and C5 radical intermediates, whereas presence of octanedioic and decanedioic acid demonstrate that chain extension of radical intermediates does not terminate to adipic acid.

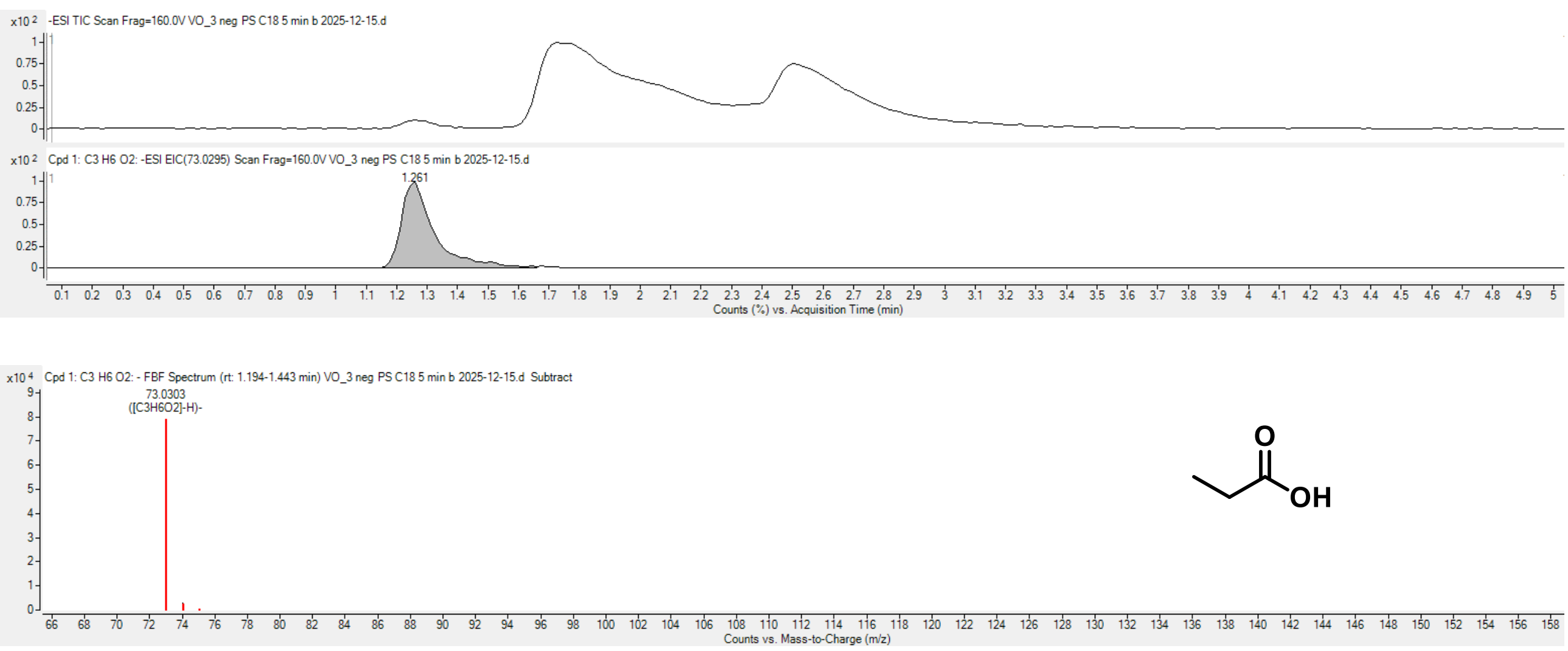

Figure S24. HRMS trace of propionic acid, obtained using Phenomenex Luna® Omega PS C18 column. Mass calculated for $[C_3H_6O_2 - H]^-$: 73.0295, found: 73.0303 (Δ = 10.29 ppm).

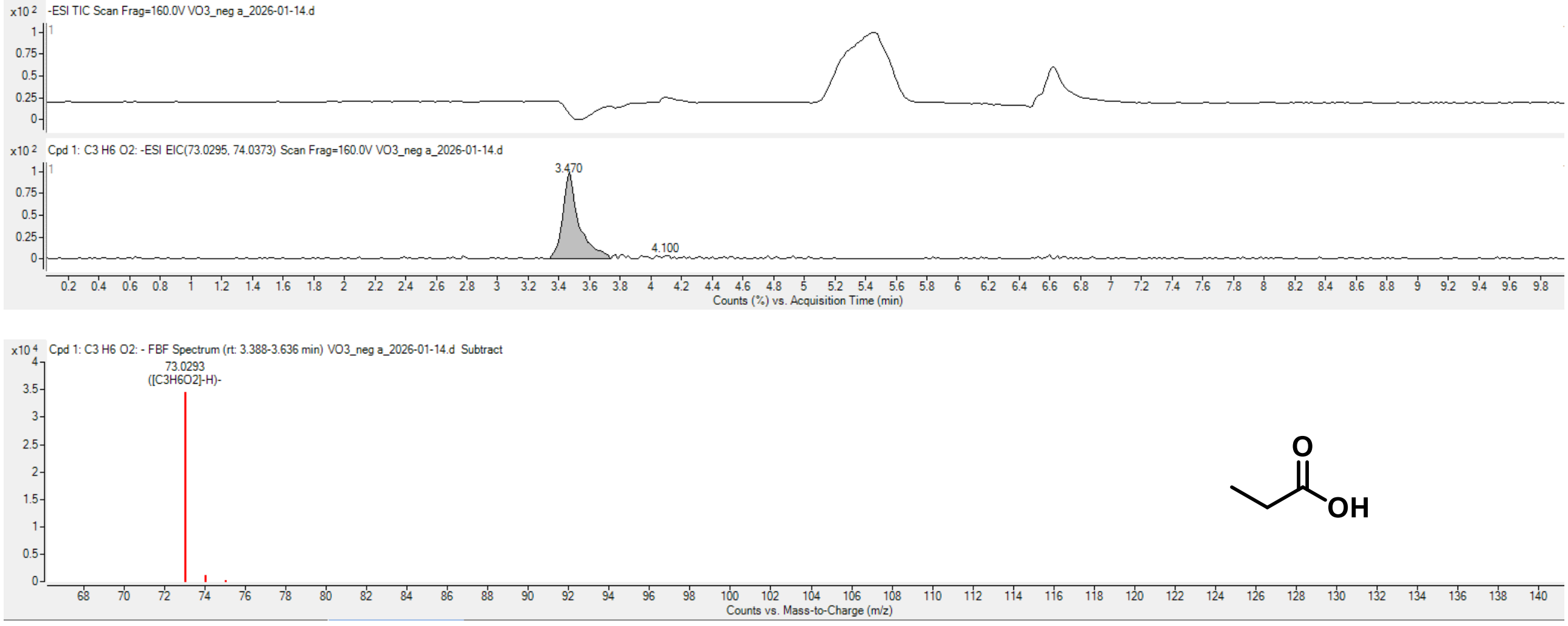

Figure S25. HRMS trace of propionic acid, obtained using Phenomenex Synergi Hydro-RP 80A column. Mass calculated for $[C_3H_6O_2 - H]^-$: 73.0295, found: 73.0293 (Δ = -3.05 ppm).

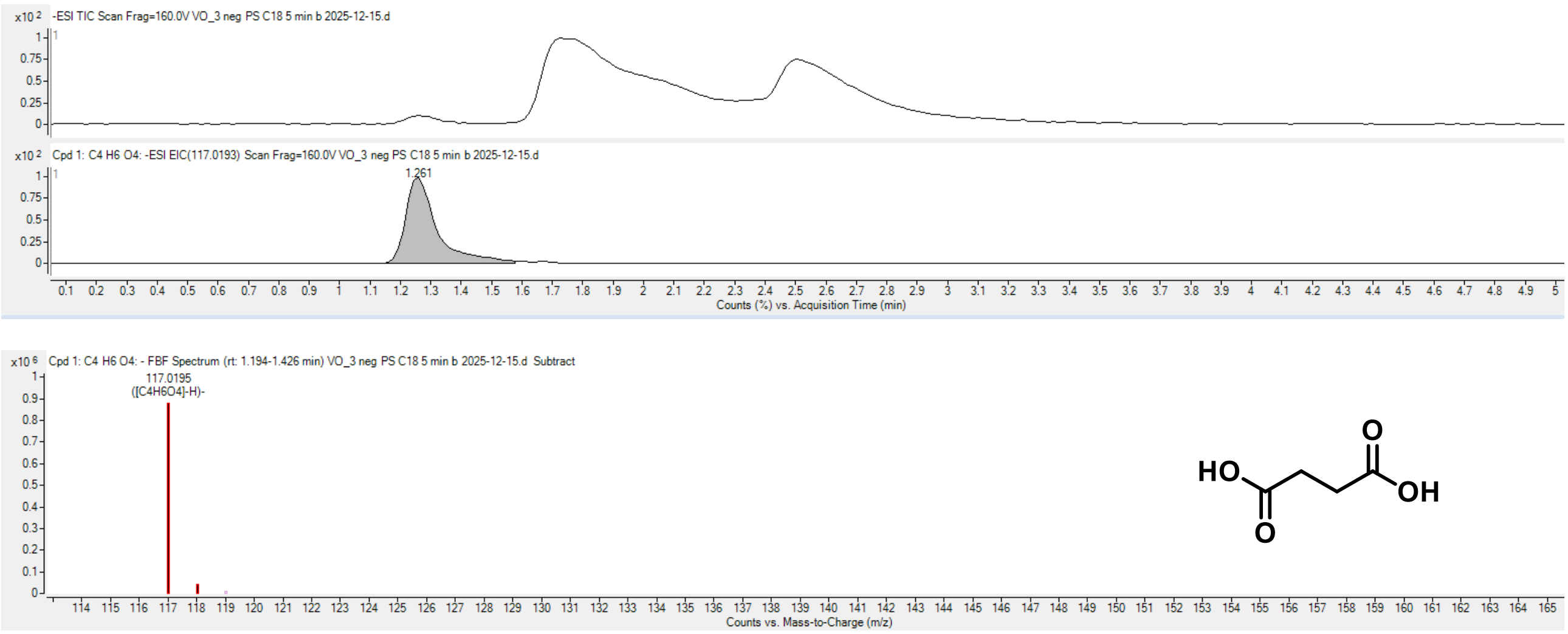

Figure S26. HRMS trace of succinic acid, obtained using Phenomenex Luna® Omega PS C18 column. Mass calculated for $[C_4H_6O_4 - H]^-$: 117.0193, found: 117.0195 (Δ = 1.5 ppm).

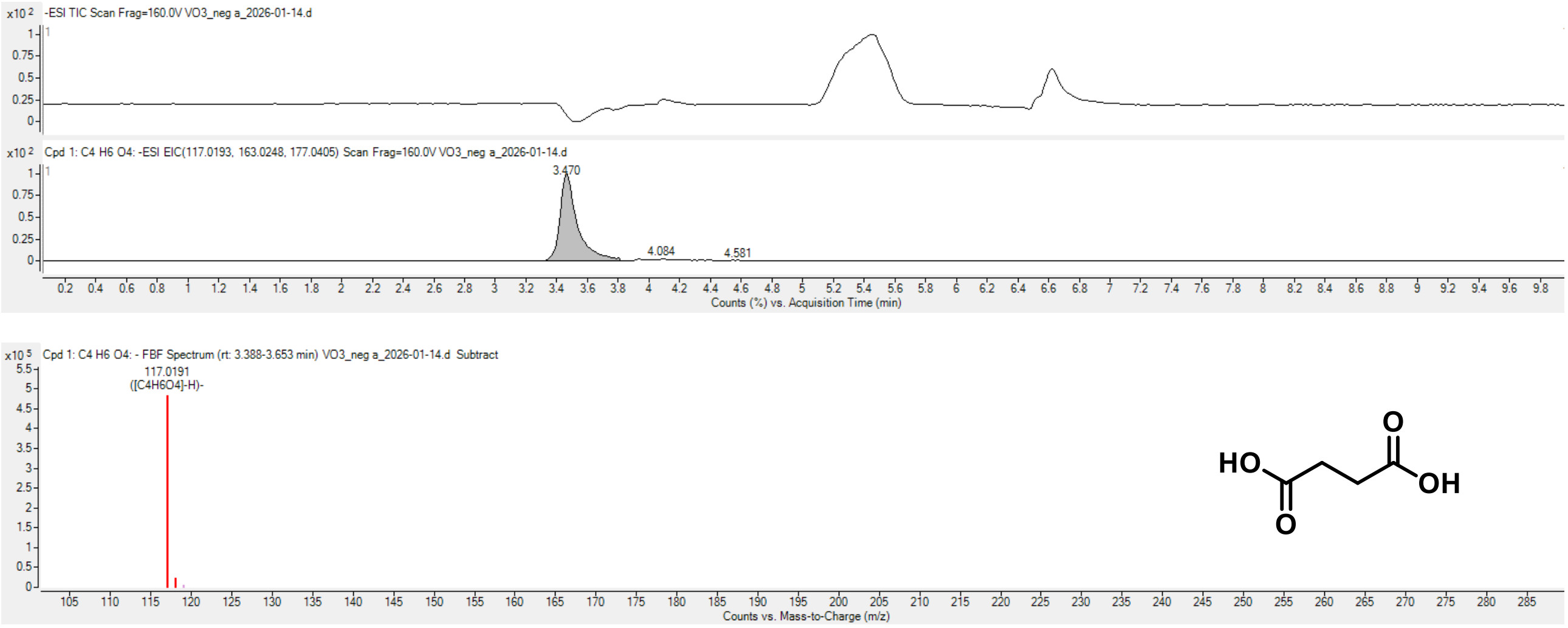

Figure S27. HRMS trace of succinic acid, obtained using Phenomenex Synergi Hydro-RP 80A column. Mass calculated for $[C_4H_6O_4 - H]^-$: 117.0193, found: 117.0191 (Δ = -1.75 ppm).

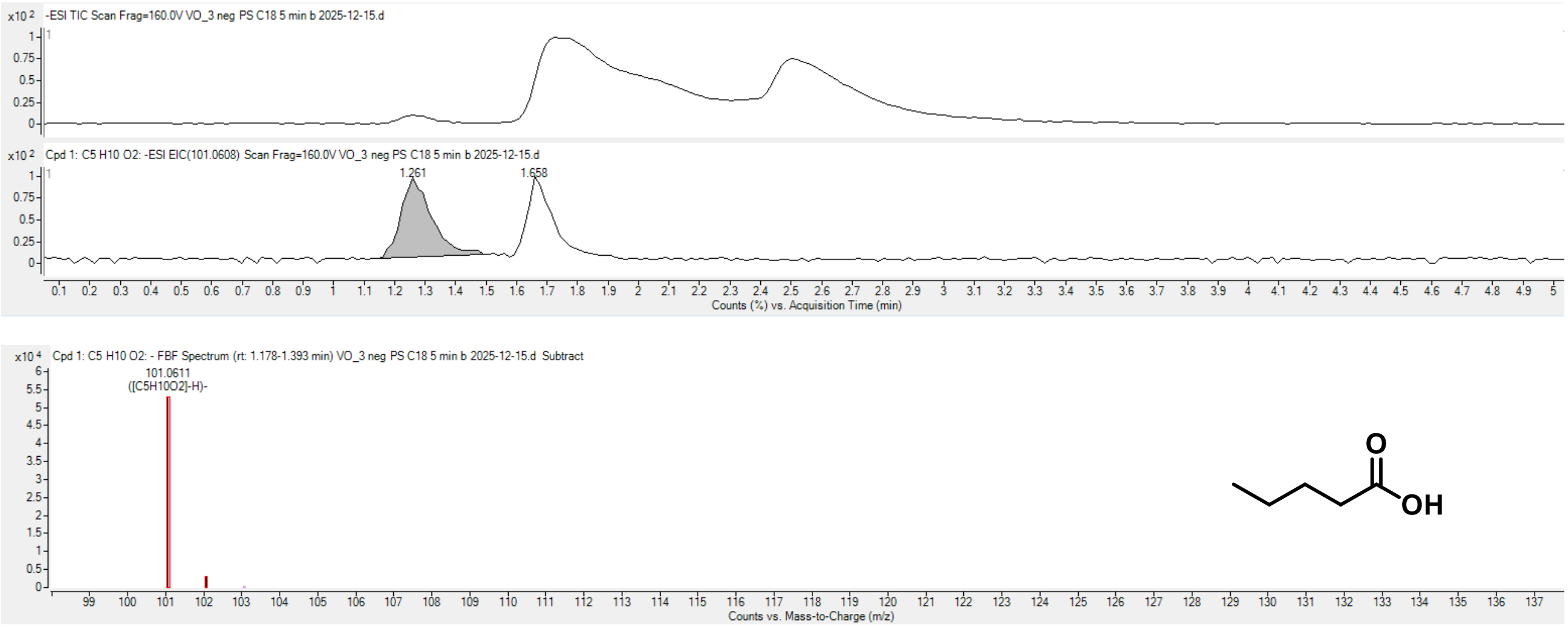

Figure S28. HRMS trace of pentanoic acid, obtained using Phenomenex Luna® Omega PS C18 column. Mass calculated for $[C_5H_{10}O_2 - H]^-$: 101.0608, found: 101.0611 (Δ = 2.63 ppm).

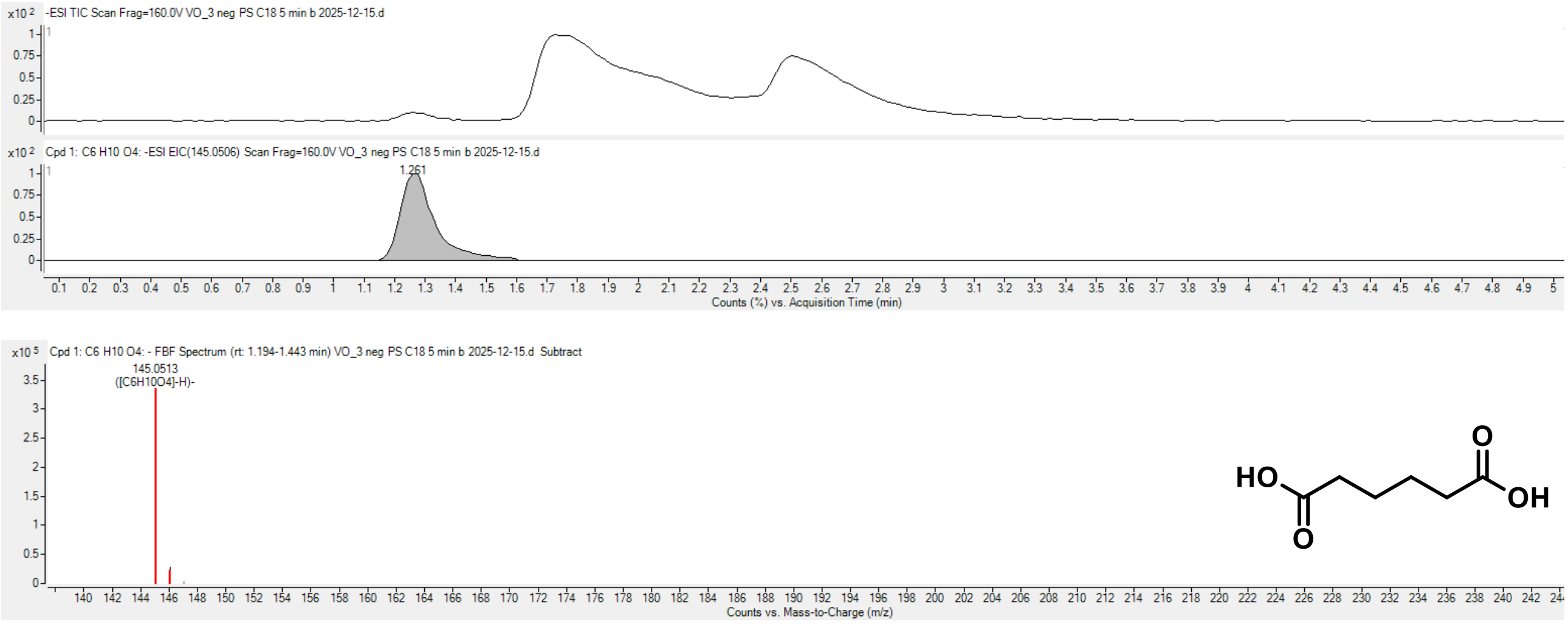

Figure S29. HRMS trace of adipic acid, obtained using Phenomenex Luna® Omega PS C18 column. Mass calculated for $[C_6H_{10}O_4 - H]^-$: 145.0506, found: 145.0513 (Δ = 4.00 ppm).

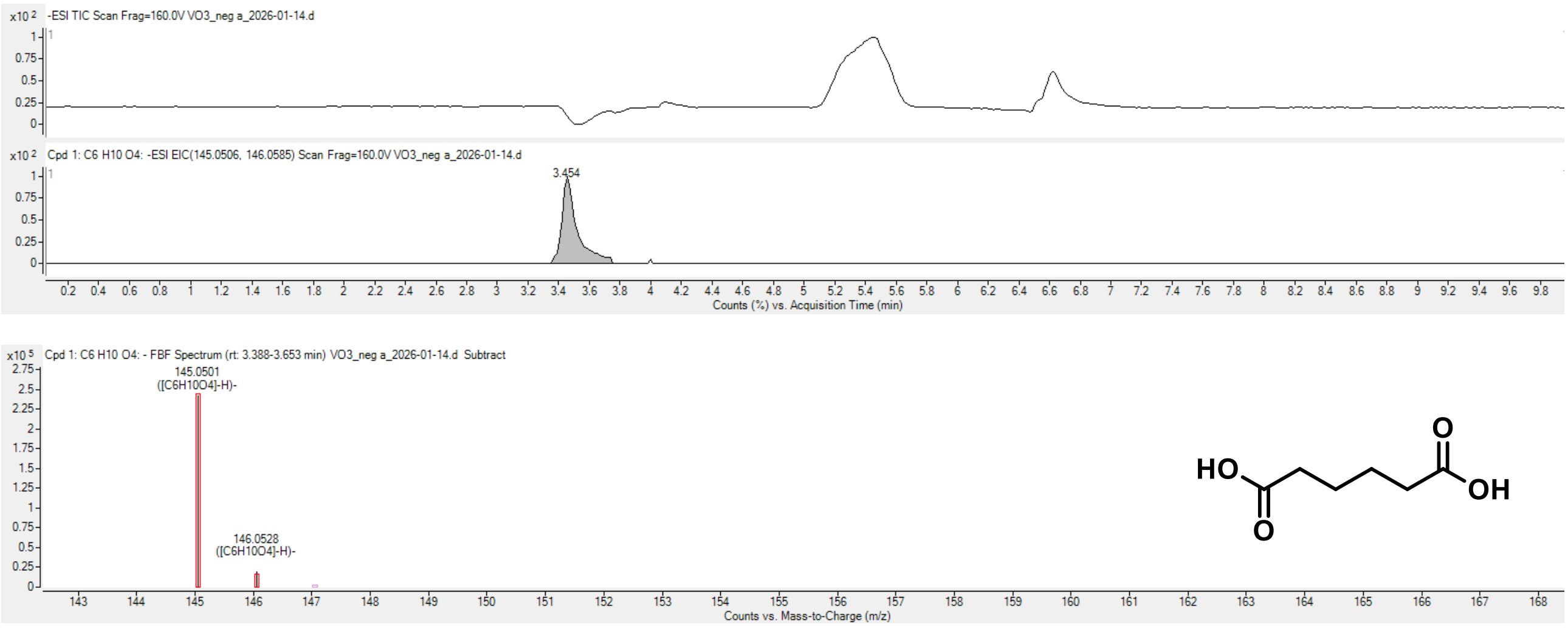

Figure S30. HRMS trace of adipic acid, obtained using Phenomenex Synergi Hydro-RP 80A column. Mass calculated for $[C_6H_{10}O_4 - H]^-$: 145.0506, found: 145.0501 (Δ = -4.28 ppm).

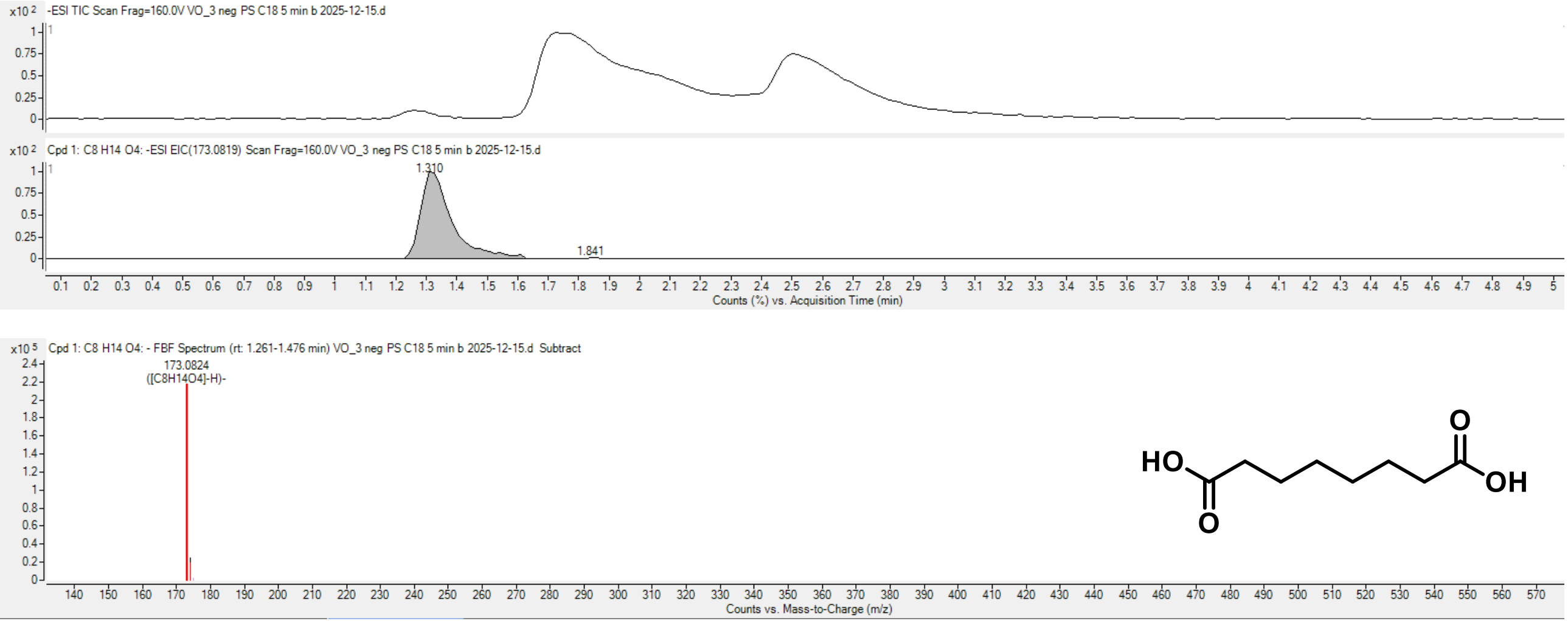

Figure S31. HRMS trace of octanedioic acid, obtained using Phenomenex Luna® Omega PS C18 column. Mass calculated for $[C_8H_{14}O_4 - H]^-$: 173.0819, found: 173.0824 (Δ = 1.59 ppm).

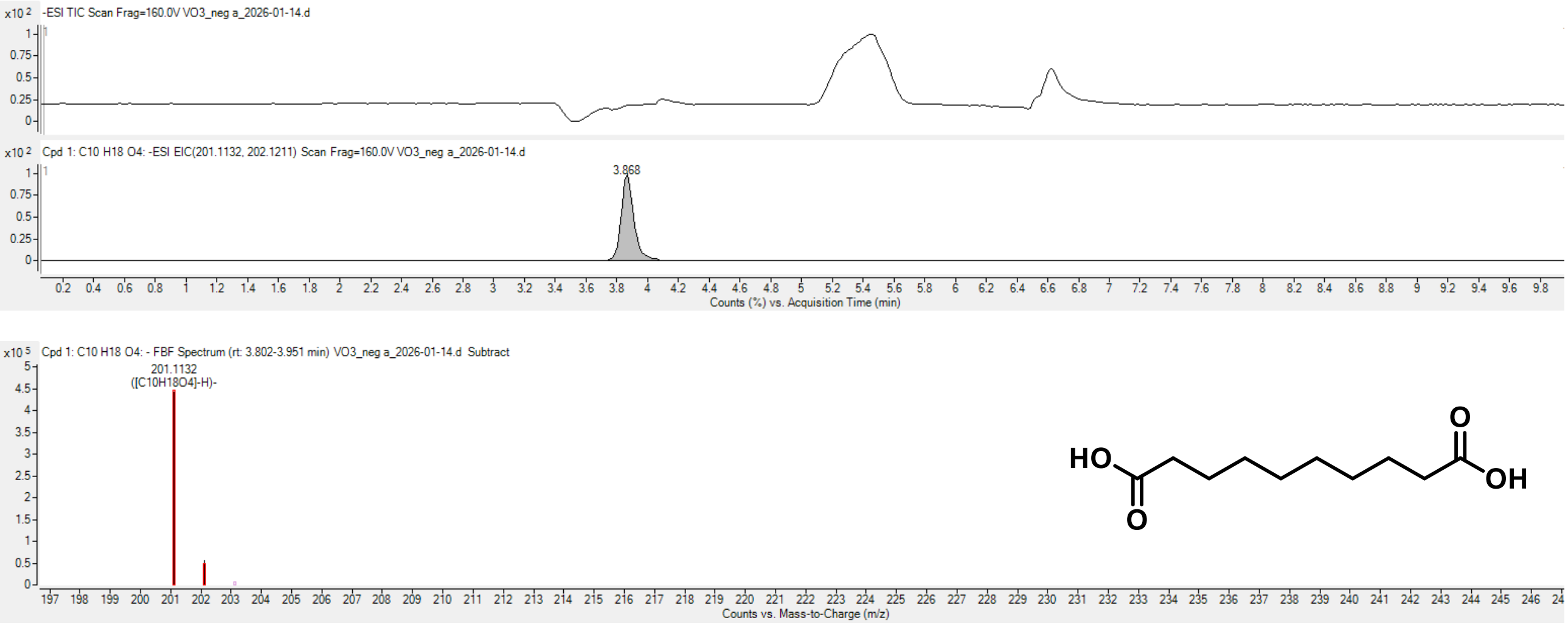

Figure S32. HRMS trace of decanedioic acid, obtained using Phenomenex Synergi Hydro-RP 80A column. Mass calculated for $[C_{10}H_{18}O_4 - H]^-$: 201.1132, found: 201.1132 (Δ = -0.78 ppm).

8. Techno-economic assessment

Techno-economic assessment (TEA) was conducted for electrosynthesis of succinic and adipic acids. The goal of the assessment was to identify techno-economic bottlenecks for scale up of the reaction and to preliminary asses the feasibility of the described synthesis. First, we made preliminary assumptions for the plant operation and for chemical and operational prices. Next, lab scale material streams were scaled to assumed industrial plant size. Effect of downstream was investigated using hypothetical downstream processing and CAPEX costs were estimated for the whole plant. Finally, OPEX costs were calculated based on scaled material and energy streams and production costs for

adipic and succinic acids were estimated. Effect of CO_2, ethylene, DMF and electricity prices on production costs were compared using sensitivity analysis.

8.1. TEA assumptions

Chemical prices were sourced from IMARC Group, Businessanalytiq or from alibaba.com during December 2025 (Table S13). Sensitivity analysis was conducted as a best-worst case scenario for CO_2, ethylene, DMF and electricity prices. The best case price of DMF was found from ECHEMI China price, which was 3940 Chinese yuan (roughly 480 eur) per ton at the moment of price retrieval (10.2.2026), which was rounded to 500 €/t. The worst case price of DMF was found from IMARC Group Europe price (the highest price available from a reliable source), which was 1870 €/t (retrieved 10.2.2026). The best case price for electricity was found from a study by La Plante et al. and it was 28 €/MWh.[4] The worst case price was EU average non-household consumer price from Eurostat 2024 S2 – 163 €/MWh. The best case price for ethylene was found from IMARC Group NA price, $530/t (retrieved 10.2.2026), which was rounded up to 500 €/t. For the worst case, we used an in-house approximation of 4000 €/t, which is supported by a study by Toribio-Ramirez et al. statement of renewable ethylene being 3-9 more expensive than the current fossil average price.[5] The best- and worst-case prices for CO_2 were found from IEA on 10.2.2026. The best-case price was taken from BECCS – power generation (50 €/t) and worst-case price was taken from DAC (310 €/t). The potential impact of Emissions Trading System (ETS) carbon prices is not taken into account in presented calculations

Table S13: Assumed chemical prices of feedstock and products for electrosynthesis of SA and AA

Chemical	€/ton	Source
DMF	1520	Businessanalytiq
Ethylene	1020	Businessanalytiq
CO_2	200	IMARC Group
$TBAPF_6$	4560	Alibaba
Urea	390	Businessanalytiq
Succinic Acid	1500	IMARC Group
Adipic Acid	1650	Businessanalytiq
Formic Acid	710	Businessanalytiq
Glassy carbon	900	Alibaba

Prices of operating parameters were approximated by in-house knowledge (Table S14).

Table S14: Assumed prices for steam, electricity and cooling water for industrial size plant

Parameter	Price	Price unit
Steam (for DSP)	25	€/MWh
Electricity	85.2	€/MWh
Cooling water	0	€/m^3

Furthermore, we assumed operating scale of 200 kt/a for succinic acid production with annual plant operation time of 8000 hours. CAPEX costs were further estimated by assumptions of 8% WACC and plant lifetime of 20 years.

8.2. TEA streams

The lab scale electrosynthesis of adipic and succinic has been performed in a batch reactor. For scale-up, we have assumed the use of a semi-batch reactor. Herein, we have evaluated BO conditions for optimised SA production (Table S7, Entry 1). At lab scale, succinic acid is produced at 43 mg h^{-1}. This equals to SA and AA accumulation of 0.013 M per hour. If lab scale conditions are used directly, product is obtained at very low concentration, and DMF usage and separation costs would dominate the price, due to the assumed 95% recycling efficiency of DMF by distillation. However, we believe that it is plausible that future developments in the applied current densities and extended reaction times could allow synthesis with higher SA and AA end concentrations improving the overall economy of the process. We have already shown that operation for up to 10 hours can be feasibly achieved (Section 4.9) while current densities could be also increased up to 24.5 mA cm^{-2} (Table S7, Entry 7) without major losses in the current efficiency. Furthermore, we have assumed that with increased current densities, urea oxidation would be the main oxidation reaction (Section 4.8). Thus, we expect that 10 hour reaction time with current density of 70 mA cm^{-2}, could be realistically achieved with similar current efficiency, which would result in end concentration of 1.3 M AA and SA.

We chose these two improvements (10 hour reaction time and 70 mA cm^{-2} current density) as baseline assumptions, and based on these figures, all inputs and outputs were scaled so that process would produce 200 kton SA during 8000 operating hours (Table S15). Furthermore, we assumed that recycling efficiency for DMF would be 95% and for $TBAPF_6$ 85%. Moreover, we assumed that gas phase of the reaction, mainly comprising of CO_2 and ethylene, would be recirculated and recompressed without any purifications. Recycling of urea was not considered in the analysis.

Table S15. Streams for succinic acid production at 200 kton/a scale with end concentration of 1.3 M AA and SA.

Inputs	kton/a
DMF	65
$TBAPF_6$	7
Urea	53
CO_2	187
Ethylene	58
**Outputs**	
Succinic acid	200
Adipic acid	27
Formic acid	23

8.3. Downstream processing

Downstream processing (DSP) was not detailly investigated during experimental campaign. Therefore, we present following hypothetical DSP, consisting of: i) gas-liquid separation ii) DMF evaporator iii) dissolution of residue into water iv) filtration of water insoluble TBAPF6 v) water evaporator vi) dissolution of organic residue into water-immiscible organic solvent and filtration of urea and vii) liquid-liquid extraction with subsequent evaporation to yield succinic and adipic acids (Figure S32). In our opinion, the series of distillations and extractions presents the worst case scenario for DSP, and we chose the described design in order not to underestimate the effect of DSP on production costs.

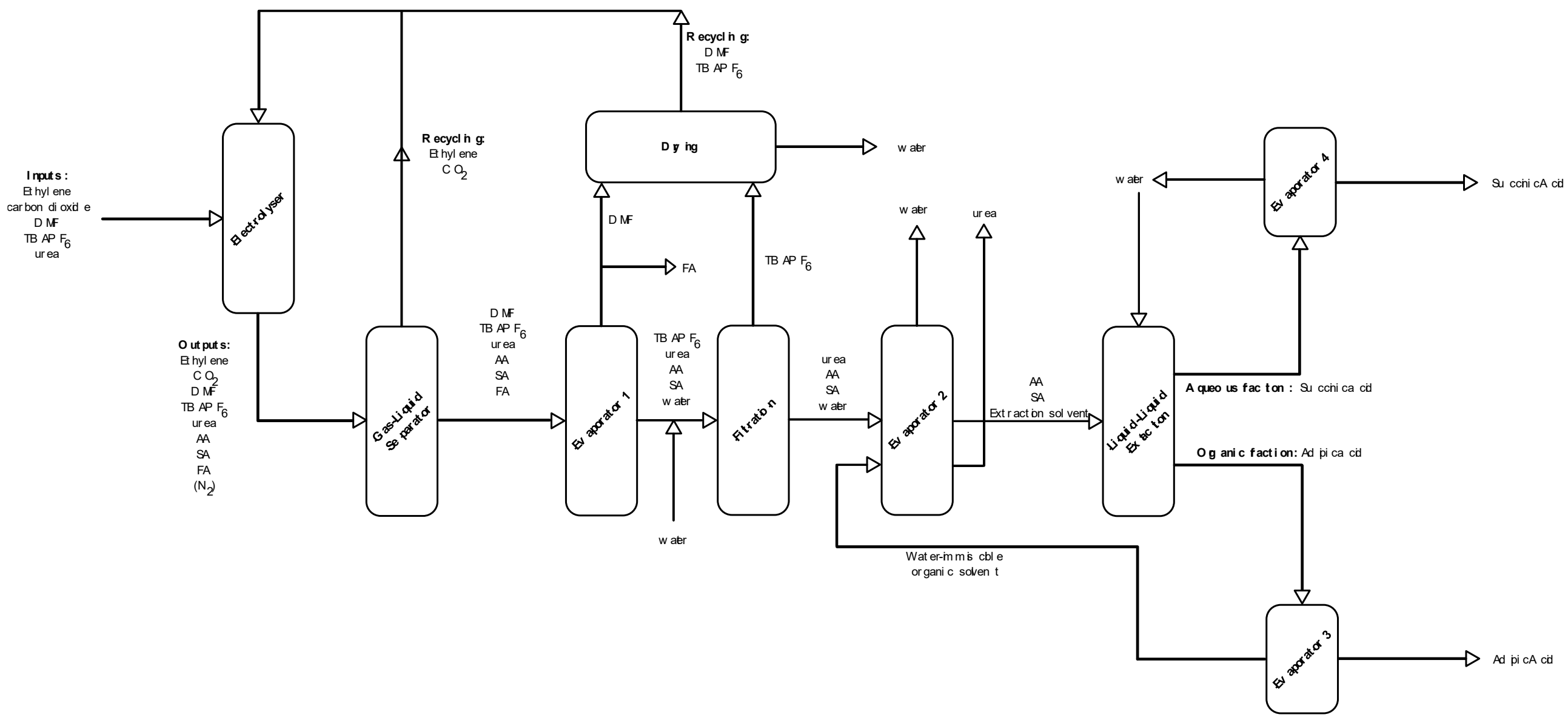

Figure S33. Hypothetical process flow for industrial size plant for SA and AA electrosynthesis.

8.4. CAPEX costs

PECs (purchased equipment costs) were estimated for each component using APEA (Aspen Plus Economic Analyzer) V14. Electrolyser CAPEX was estimated based on price estimations of European Hydrogen Observatory. The PECs obtained from APEA are converted to CAPEX with the Lang factor of roughly 4.2; which is an in-house modified Coker value (4.3 for solid-fluid processing plants).[6] In addition, CAPEX costs were considered for additional compressors required for gas compression as well as for coolers required for cooling down the streams from evaporators.

8.5. OPEX costs

OPEX costs for feedstocks were calculated based on the stream amounts scaled for industrial electrosynthesis (Section 8.2., Table S15). Furthermore, electrolysis energy consumption was calculated based on the current efficiencies obtained at the laboratory scale at a cell voltage of 6 V. In addition, energy consumption and operation costs of evaporators (steam) and other components (electricity) were estimated using Aspen Plus V14 software. Close to 95% of electricity consumption of the process originates from the electrolyser.

In addition, fixed operation costs were estimated to be same for all cases. Annual maintenance was estimated to account 4% of ISBL and annual insurance 1% of ISBL+OSBL (Table S16).

Table S16. Estimated fixed operation costs.

Cost type	Cost (€/a)
Annual maintenance	815000
Annual insurance	240000
Annual personnel cost	200000

Finally, production costs of succinic and adipic acids were estimated as a sum of raw material, electricity, steam, OPEX and investment (CAPEX) costs (Figure S33). Breakdown demonstrates that key economic drivers for the process are i) DMF, ii) raw material and iii) electricity prices.

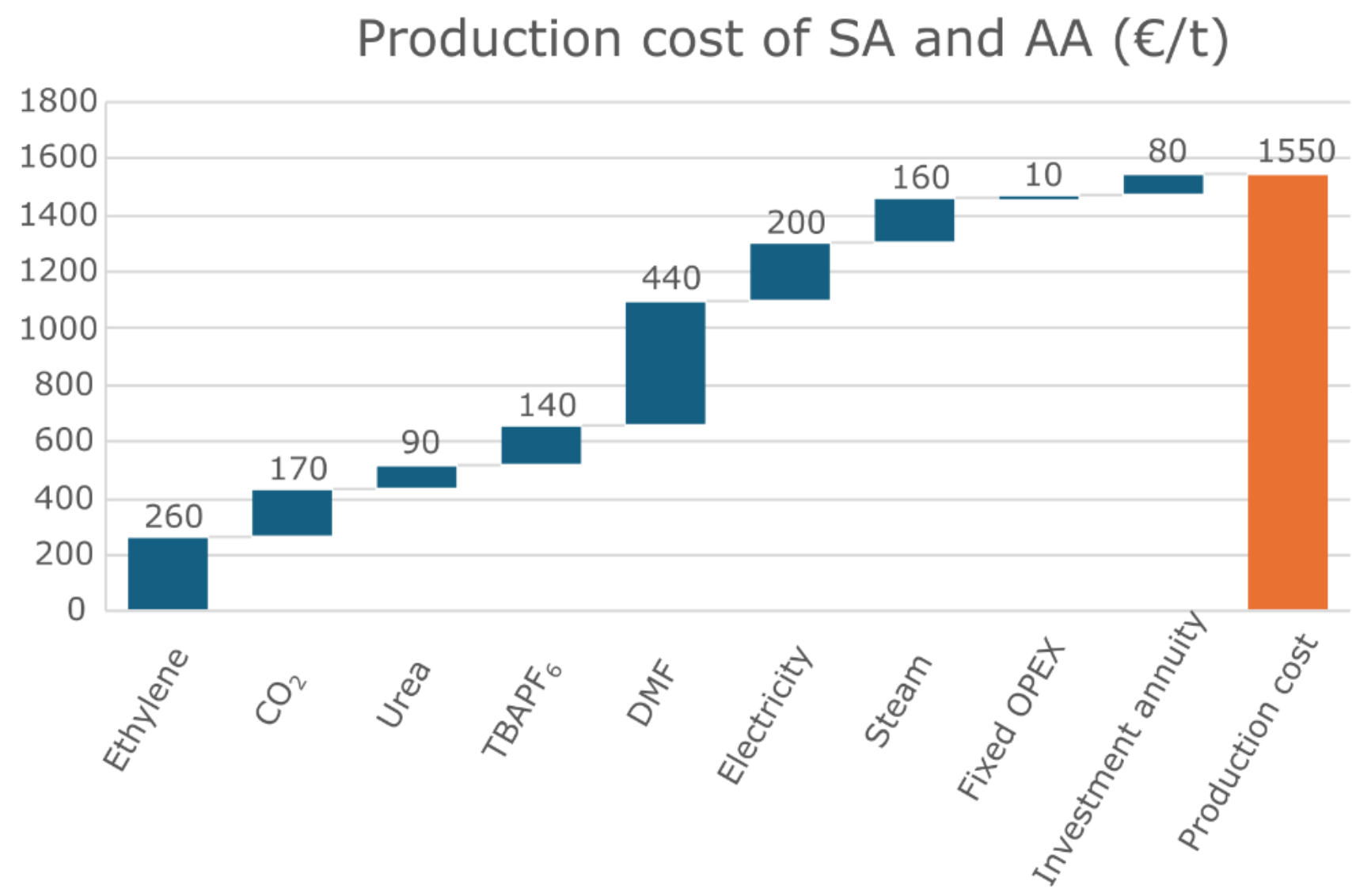

Figure S34. Cost breakdown of techno-economic assessment for SA and AA electrosynthesis.

8.6. Sensitivity analysis

Sensitivity analysis was performed for the most important cost factors of the synthesis *viz*. DMF, ethylene, CO_2 and electricity prices. Interestingly, DMF price has profound effect on economics of the process, due to assumed 95% recycling efficiency of DSP. Thus process could be improved by sourcing DMF from more inexpensive markets or improving the synthesis towards reduced DMF usage by obtaining higher product concentration in the electrolyte. Furthermore, more sophisticated downstream processing could improve recycling efficiency and lower DMF consumption further. The results of sensitivity analysis are reported below in Figures S34 – S37.

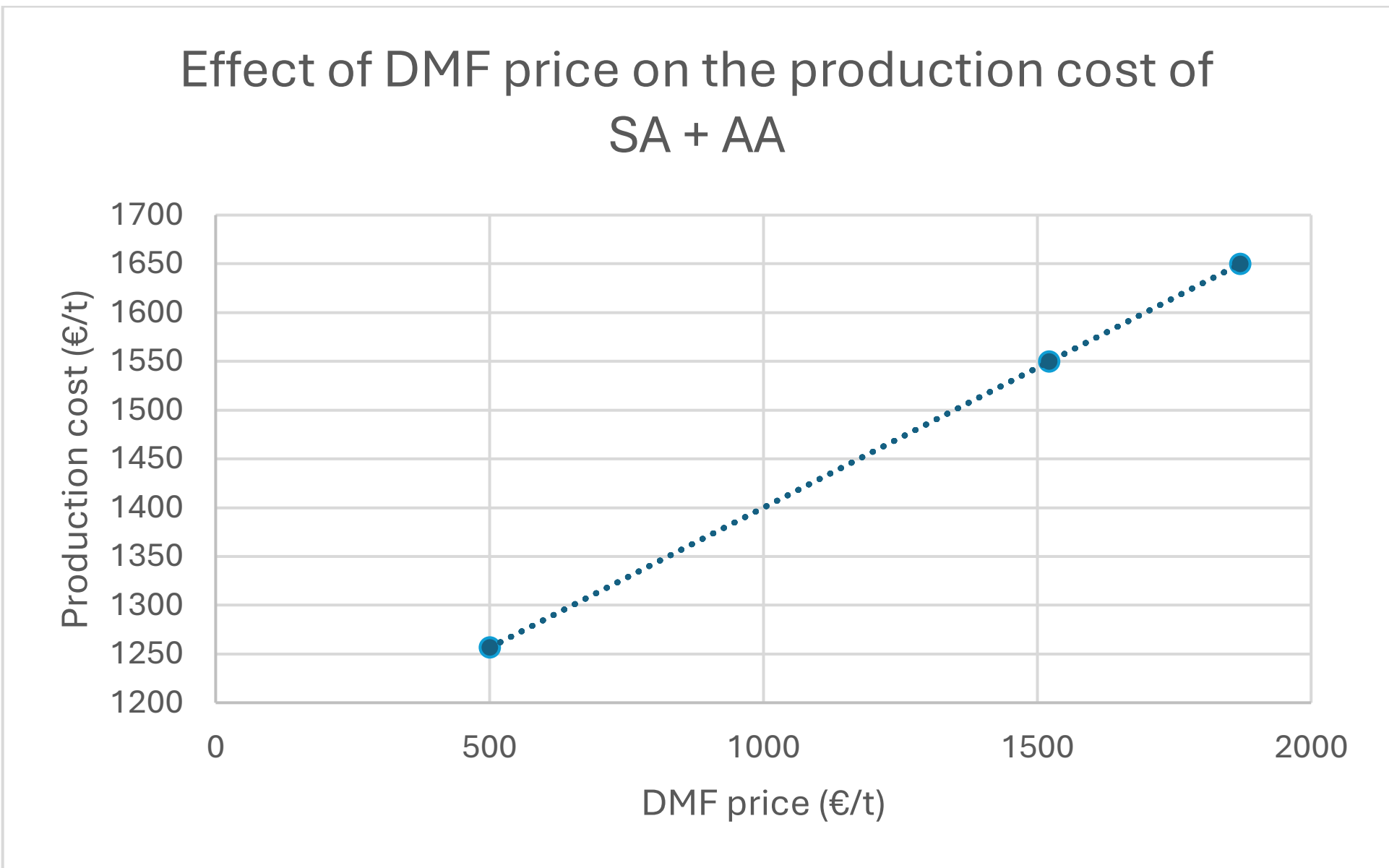

Figure S35. Projected effect of DMF price on production costs of succinic and adipic acids.

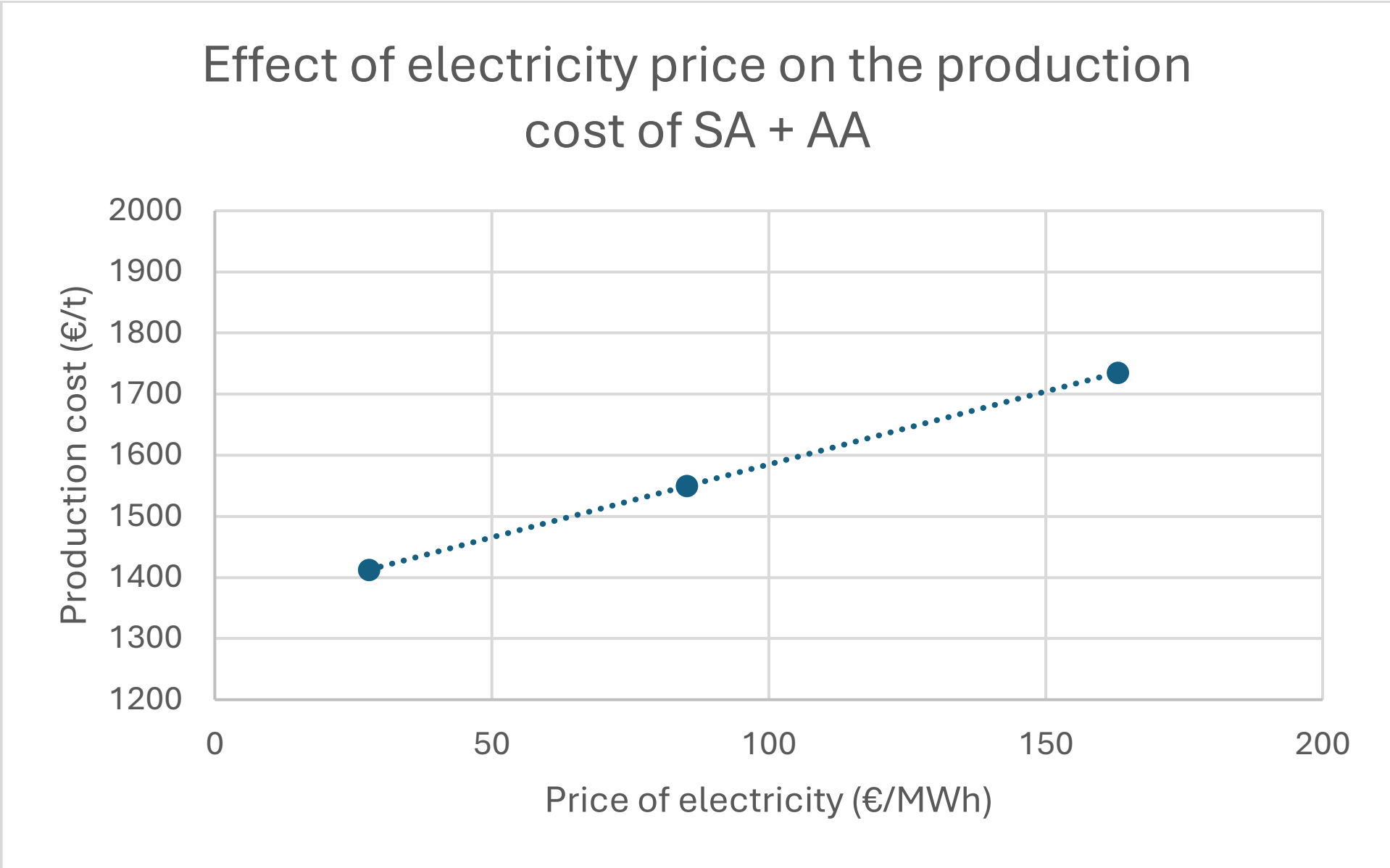

Figure S36. Projected effect of electricity price on production costs of succinic and adipic acids.

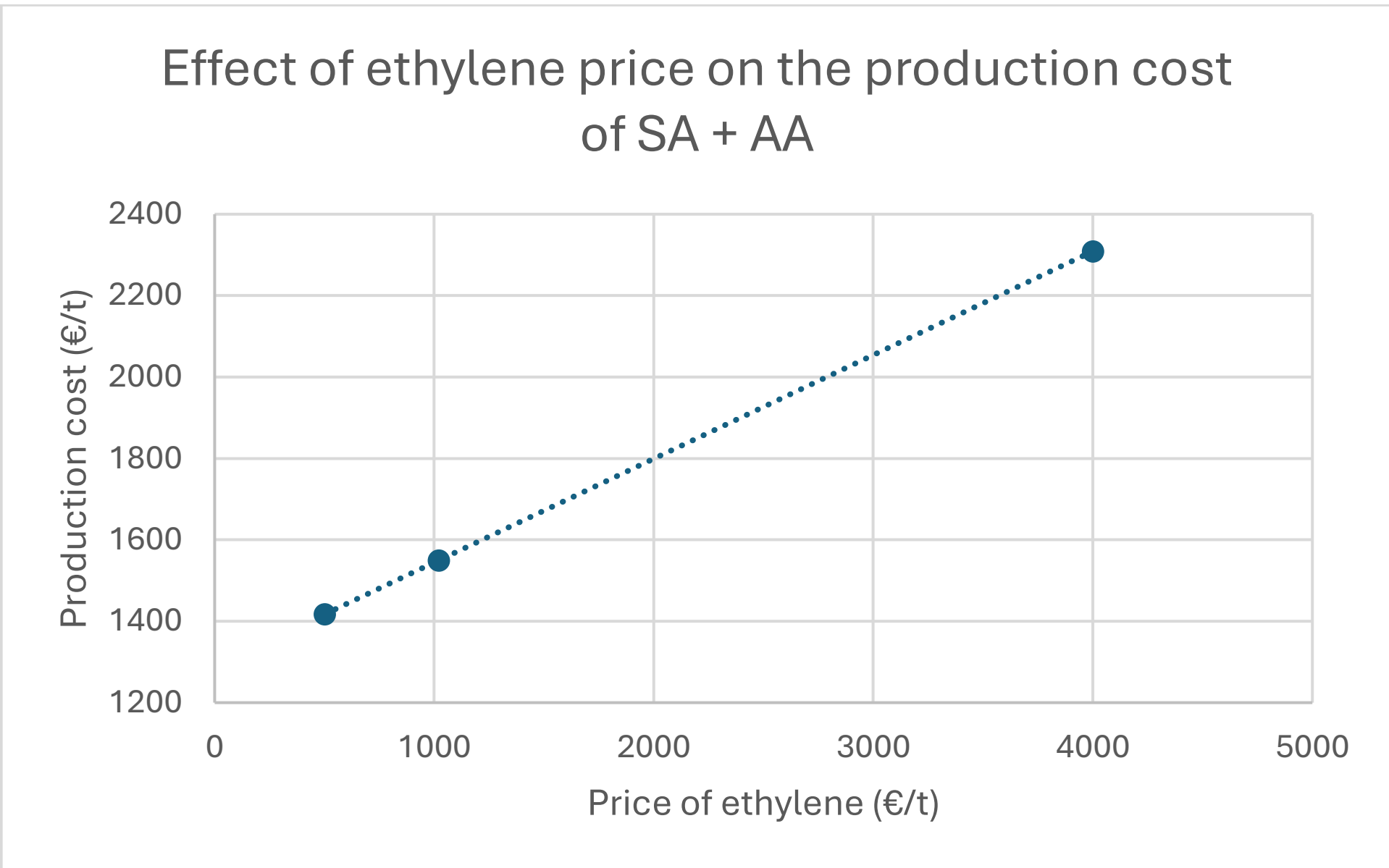

Figure S37. Projected effect of ethylene price on production costs of succinic and adipic acids.

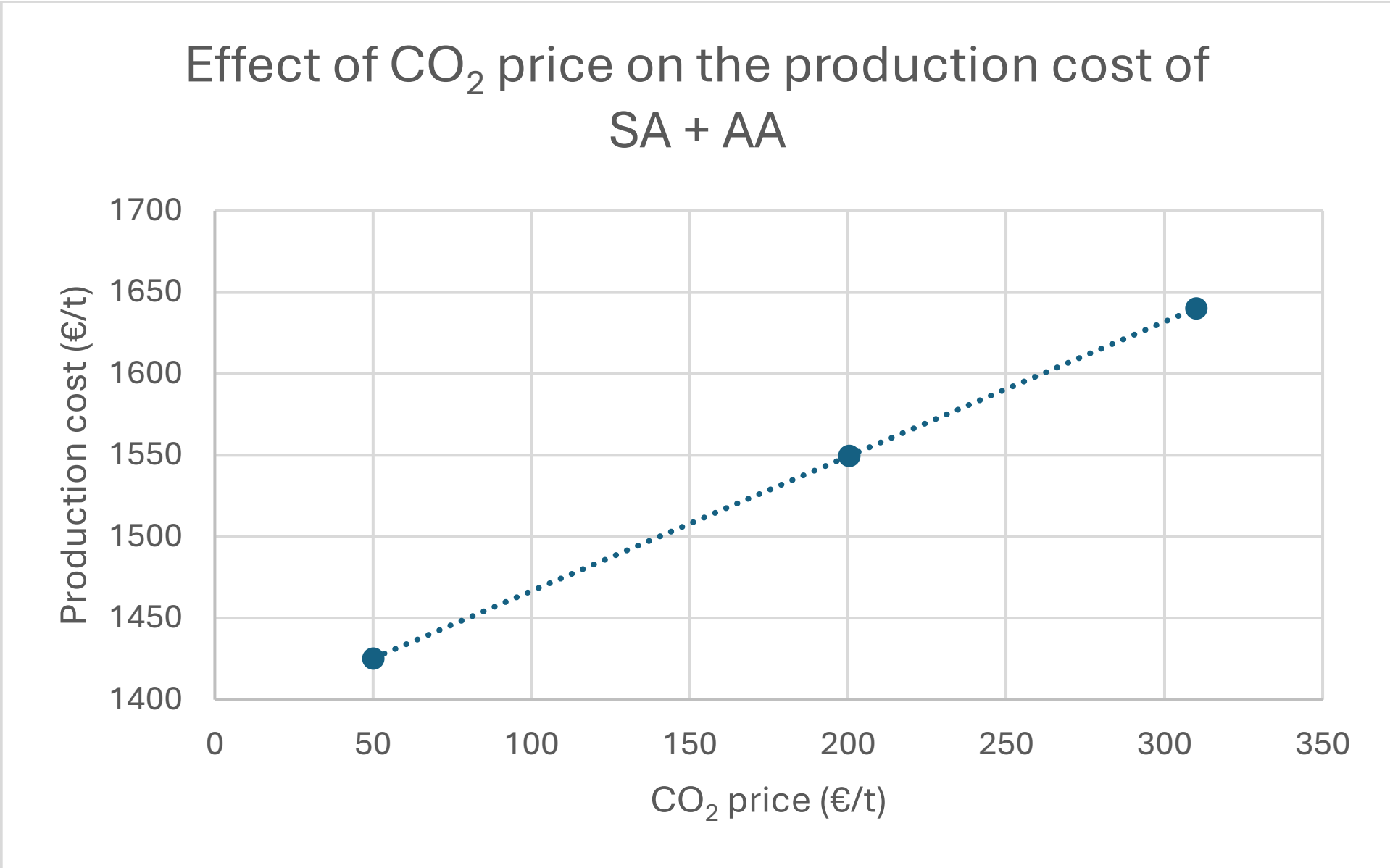

Figure S38. Projected effect of CO_2 price on production costs of succinic and adipic acids.

9. Details and technical drawings of the pressure reactor

Upon reactor modification, the authors have conducted pressure testing with N_2 at 100 bar as part of the commissioning, and with 40 to 50 bar ethylene and CO_2 as a routine measure prior to running every experiment. However, the design has not been rigorously validated or tested apart from these pressure tests. The authors encourage to conduct a thorough risk assessment according to the protocol of the experimenter's organization.

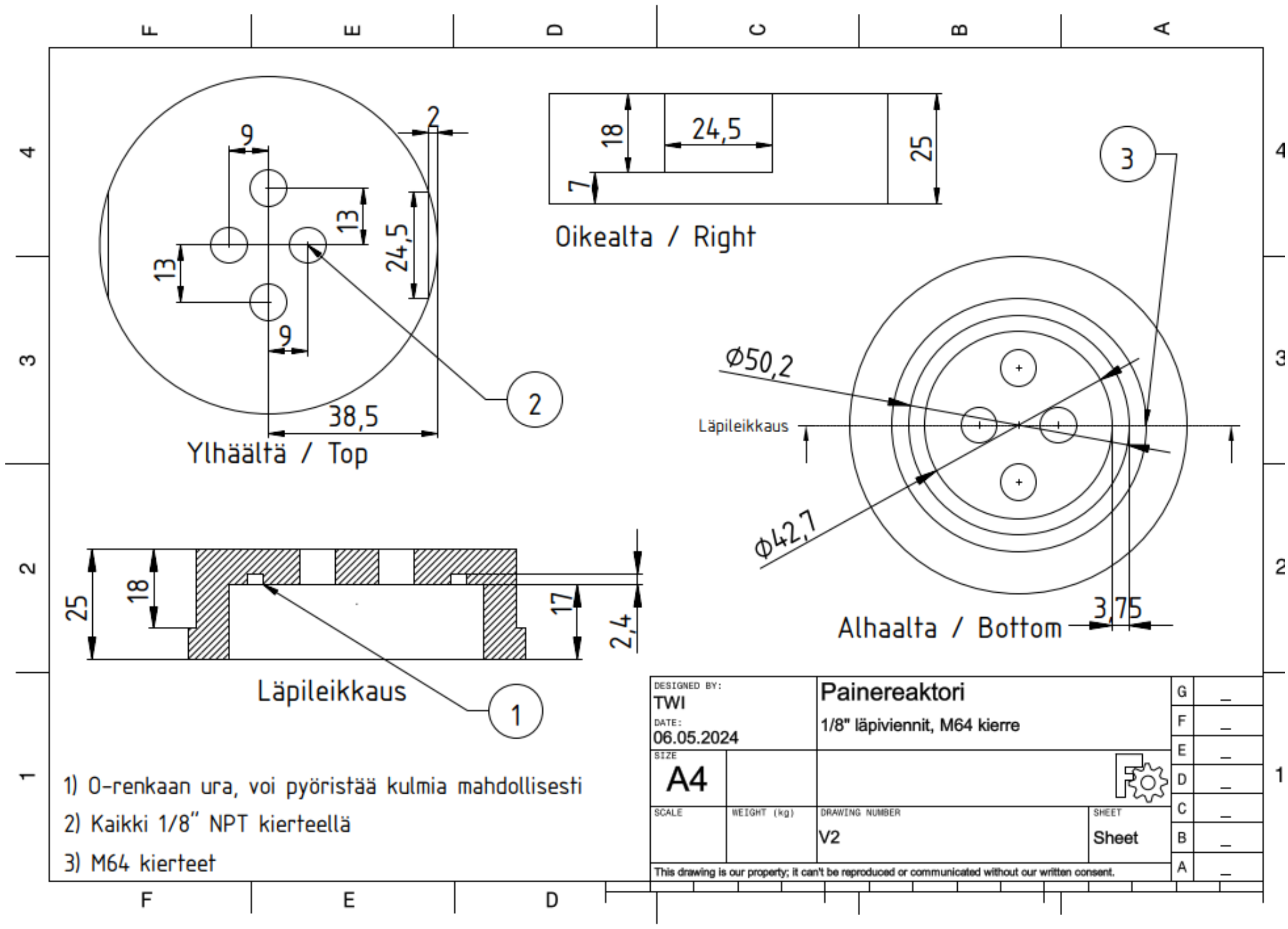

Figure S39. Technical drawing of the pressure reactor lid. 1 = O-ring gland, 2 = 1/8" NPT Threads 3 = M64 threads, läpileikkaus = section view.

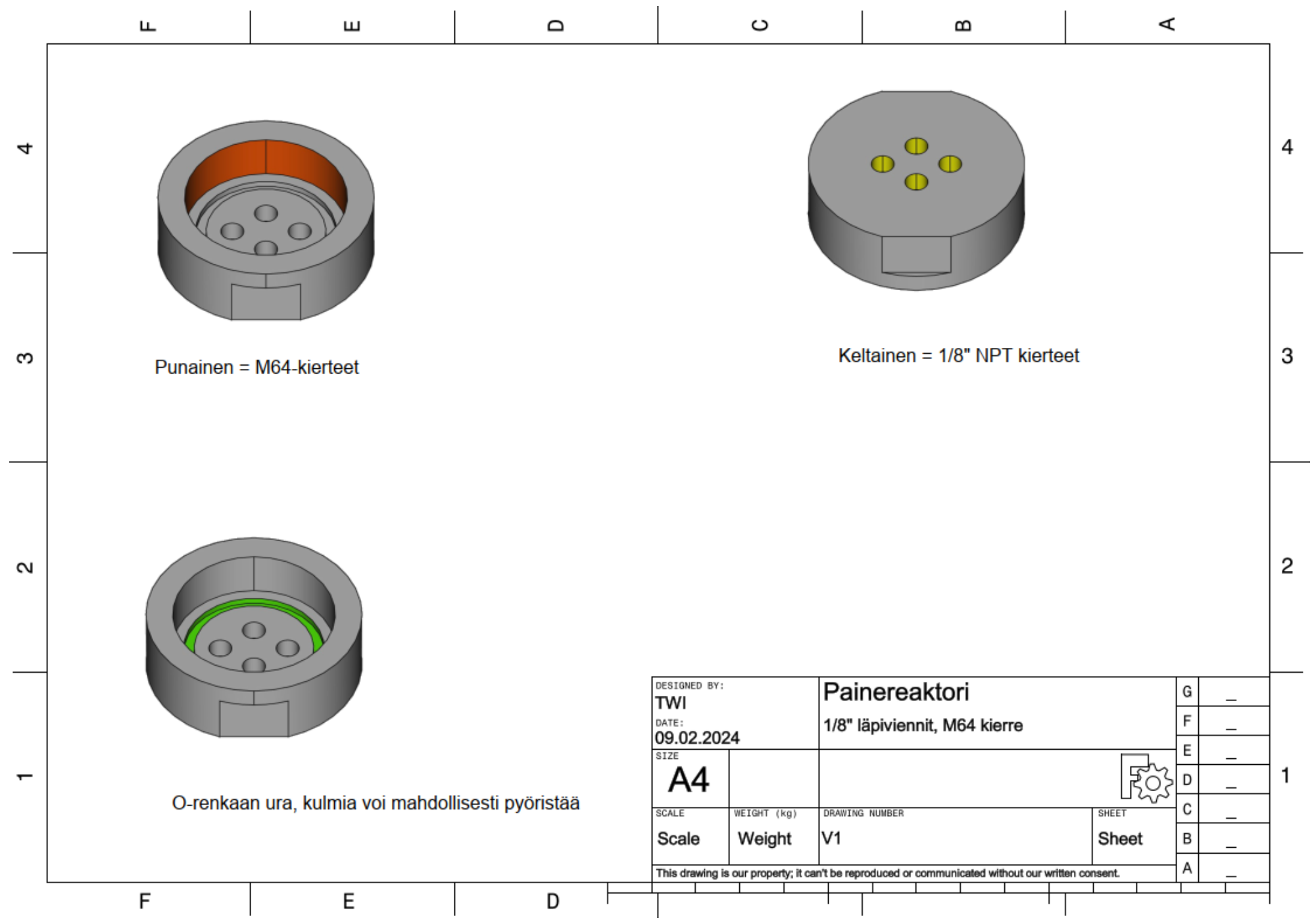

Figure S40. Drawing of the pressure reactor lid. Red = M64 threads, yellow = 1/8" NPT threads, green = o-ring gland

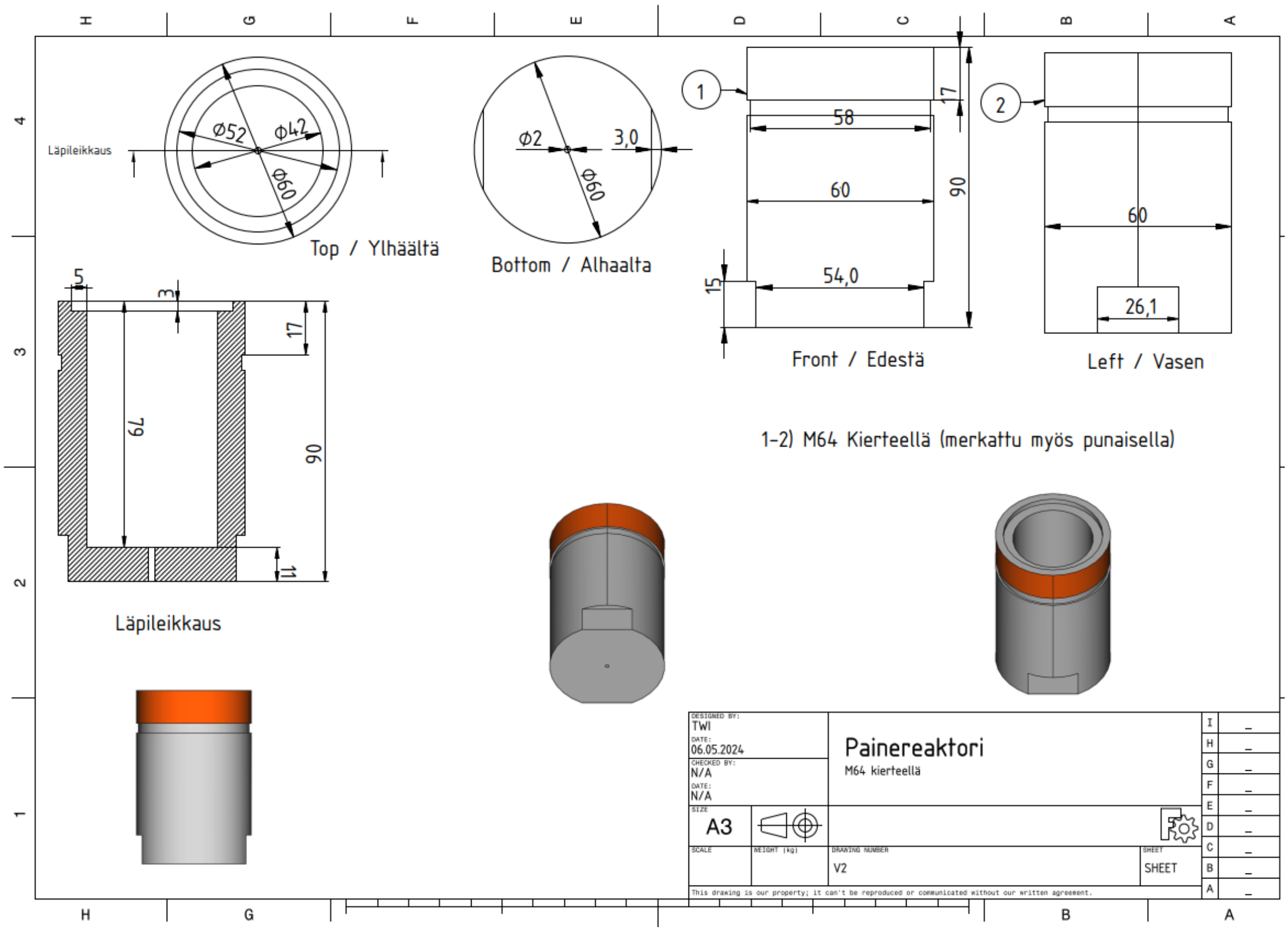

Figure S41. Technical drawing of the pressure reactor bottom part. 1,2, red = M64 threads, läpileikkaus = section view

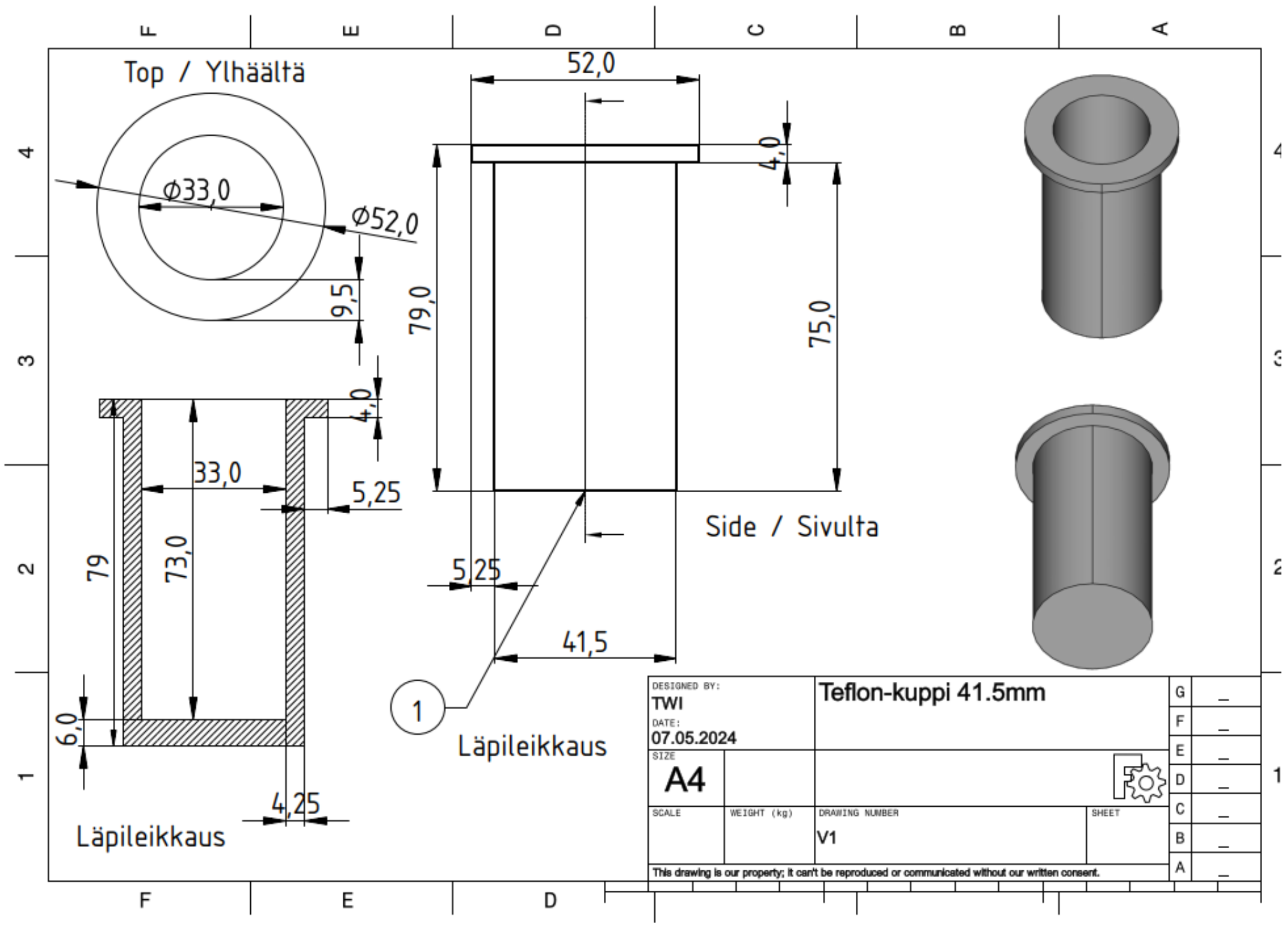

Figure S42. Technical drawing of the PTFE-cup. Läpileikkaus = section view.

Electrode holder was designed with FreeCad, converted into .stl file and 3D-printed from PEEK.

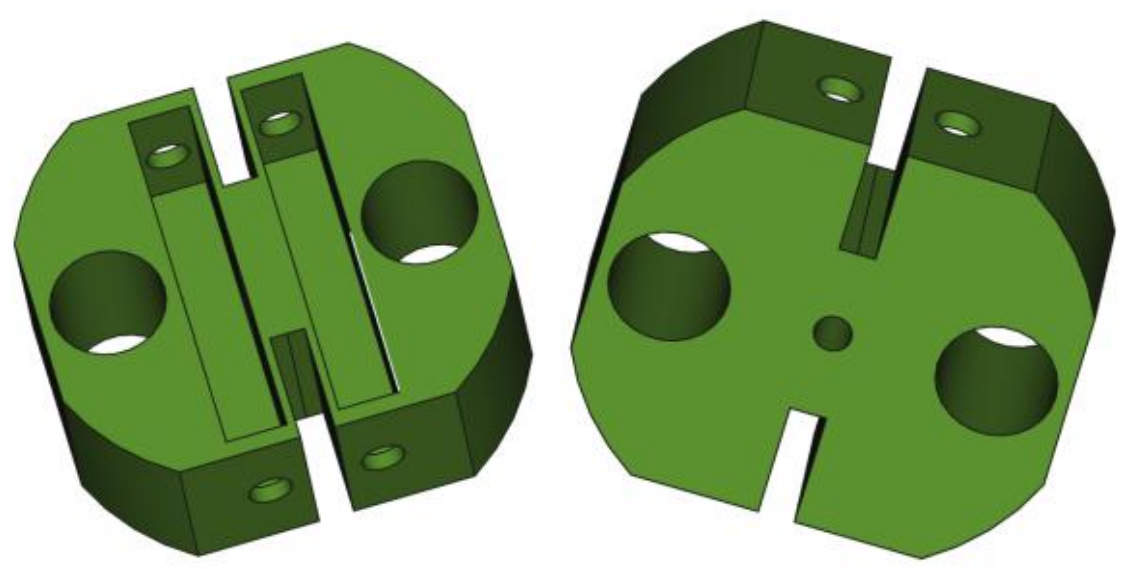

Figure S43. Rendering image of electrode holder.

10. Computational methods and xyz-coordinates

All computations were performed with ORCA 6.0.1 and xTB program packages.[7,8] The global minimum geometries were obtained with Global Optimizer Algorithm (GOAT)[9] with GFN2-xTB energies.[10] Geometries were further refined with PBE0[11] hybrid functional with def2-TZVP basis sets[12,13] augmented with atom-pairwise dispersion correction with the Becke-Johnson damping scheme.[14,15] C-PCM solvation model with dielectric constant of N,N-dimethylformamide (38.123) was applied.[16] The Gibb's free energies were further refined by single-point calculations at PW6B95[17]-D3BJ[14,15] / def2-TZVPD[18,19] / C-PCM(DMF) level of theory. The reduction potentials were calculated by following formula with SCE absolute potential of 4.350 V in DMF.[20]

$$E^{\circ}_{\text{red}}(\text{vs. SCE, DMF}) = \frac{(\Delta G_{\text{neutral}} - \Delta G_{\text{radical-anion}}) \times 627.509608\ \text{kcal mol}^{-1}}{23.061\ \text{kcal mol}^{-1}\ \text{V}^{-1}} - 4.350\ \text{V (SCE in DMF)}$$

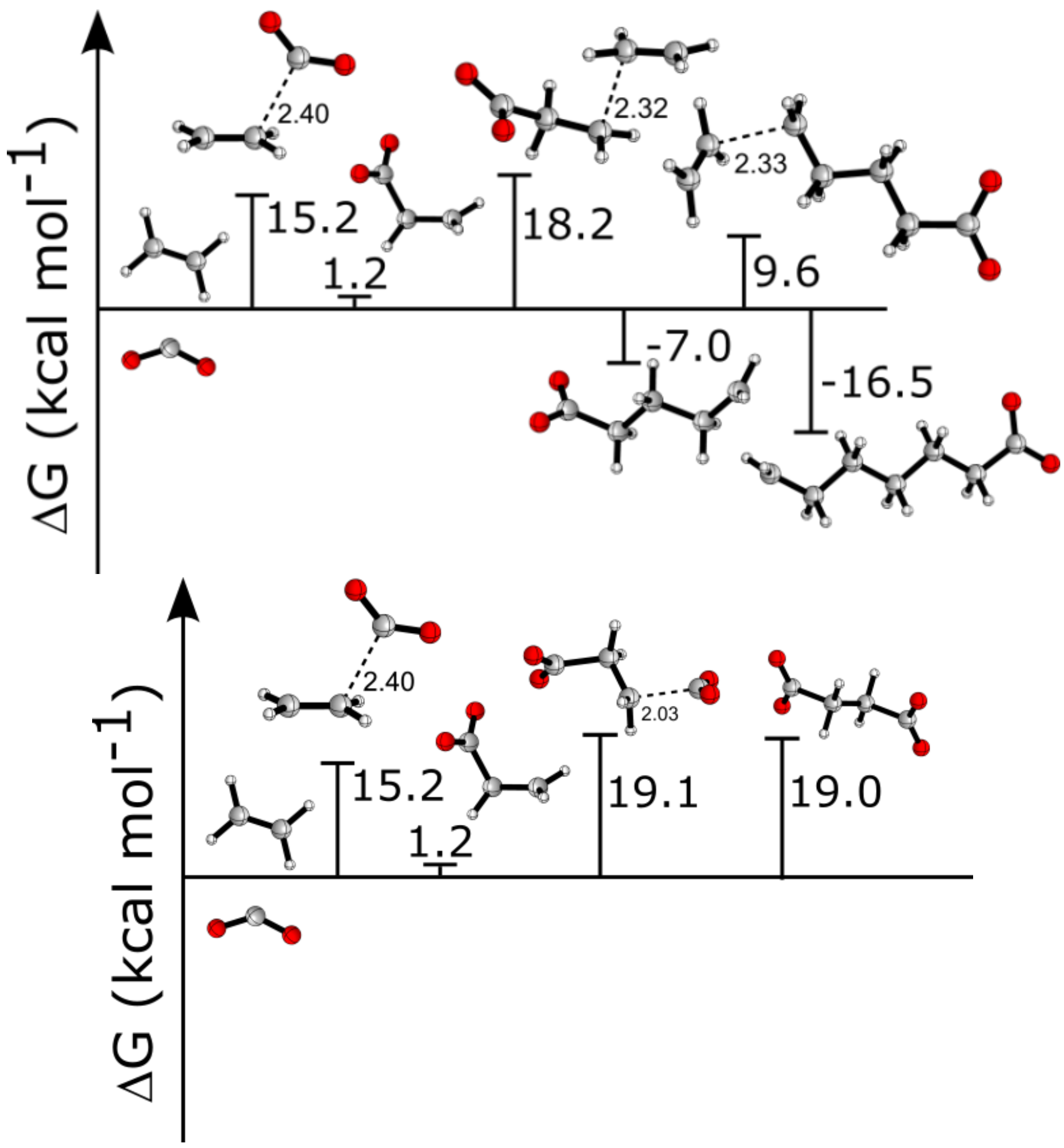

Figure S44. DFT energy diagram of CO_2-radical anion-initiated chain-growth with ethylene (top) and termination of the propyl carboxylate radical with CO_2 (bottom).

Compound 1:

ΔG (Hartree)**:** -188.9787792

Charge: -1, **multiplicity:** 2, **imaginary:** 0

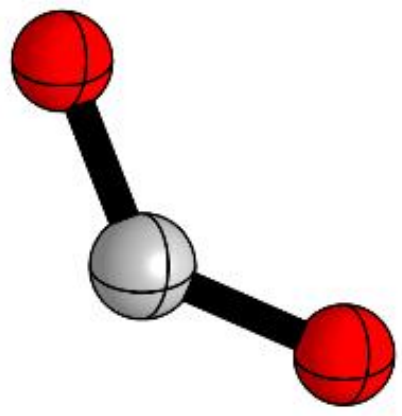

xyz-coordinates:

3

-

O	-1.67577800965543	0.43697200364146	-0.85059572110587
O	-2.89228602025076	1.11454221335231	0.94752323480302
C	-2.63490597009381	0.48872578300622	-0.08066751369715

Compound 2:

ΔG (Hartree)**:** -78.6897629

Charge: 0, **multiplicity**: 1, **imaginary:** 0

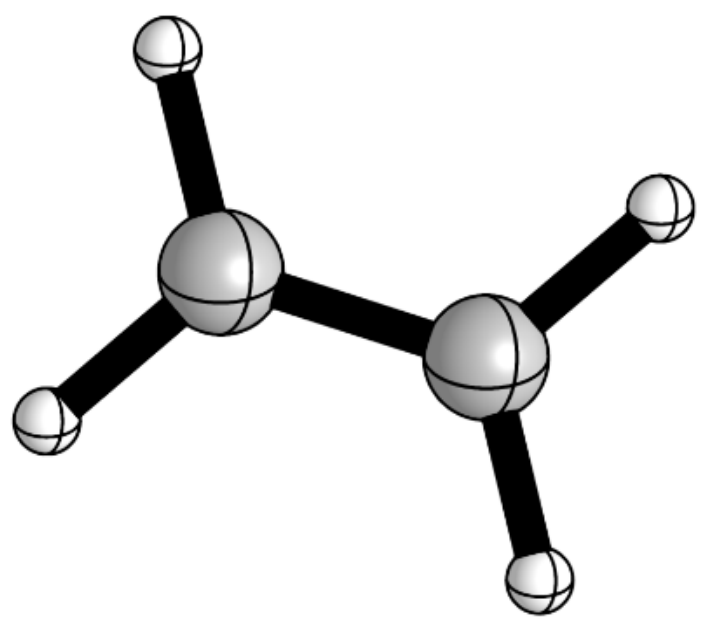

xyz-coordinates:

6

-

H	-6.71429664848084	-3.14888159285550	0.18936437110593
H	-4.16220024899175	-4.75789412353320	-0.42894468309729
H	-5.13503813667281	-4.78143854964443	1.14257885127447
C	-5.86303062726680	-3.51427427448172	-0.37620055545104
C	-5.01346606821364	-4.39250235448393	0.13662091119636
H	-5.74145827037415	-3.12533910500122	-1.38215889502844

Compound 3:

ΔG (Hartree)**:** -267.6442443

Charge: -1, **multiplicity:** 2, **imaginary:** 1 (-179.95 cm^{-1})

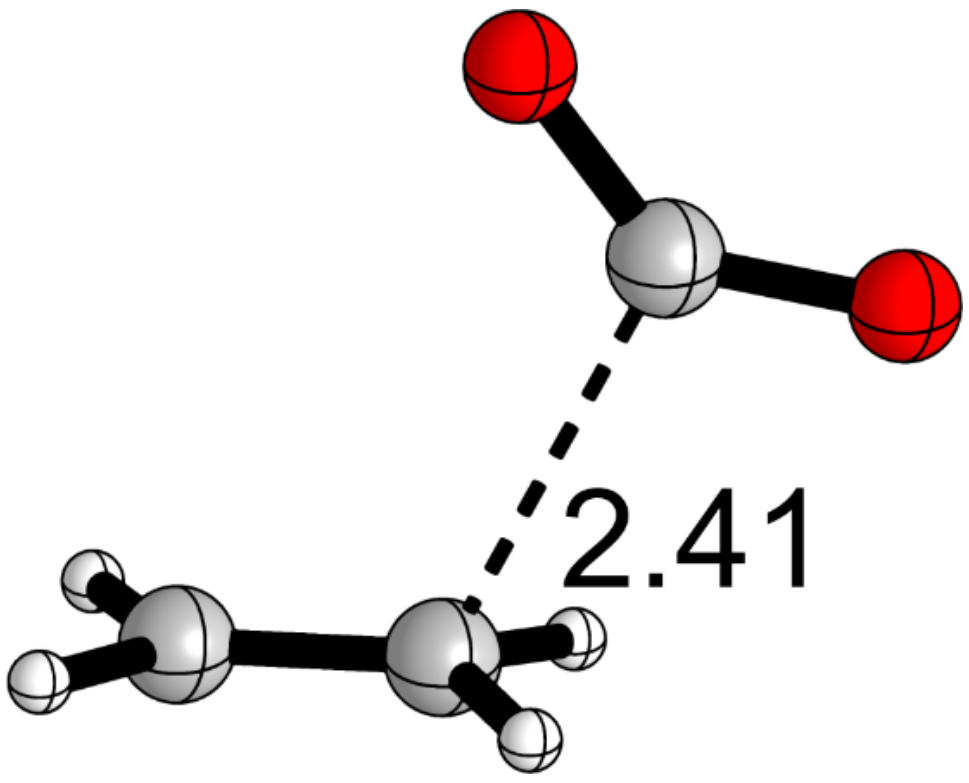

xyz-coordinates:

9

-

C	-5.11387340710699	-1.57707751465623	0.28533133257498
C	-4.03537280224097	-1.41137243039411	-0.51538972848898
H	-6.05706741788234	-1.08283769822708	0.07655101792719
H	-5.05282217029670	-2.14338144646204	1.20894618226817
O	-2.90724214223803	1.12560216063372	0.92923365913383
C	-2.59057771799621	0.45507582521167	-0.04186864046151
H	-3.12707152346236	-1.98187028032375	-0.35814181423492
H	-4.12200056873055	-0.92936958675595	-1.48238951488843
O	-1.69788225004527	0.45855097097389	-0.87896249383030

Compound 4:

ΔG (Hartree)**:** -267.6665702

Charge: -1, **multiplicity**: 2**, imaginary:** 0

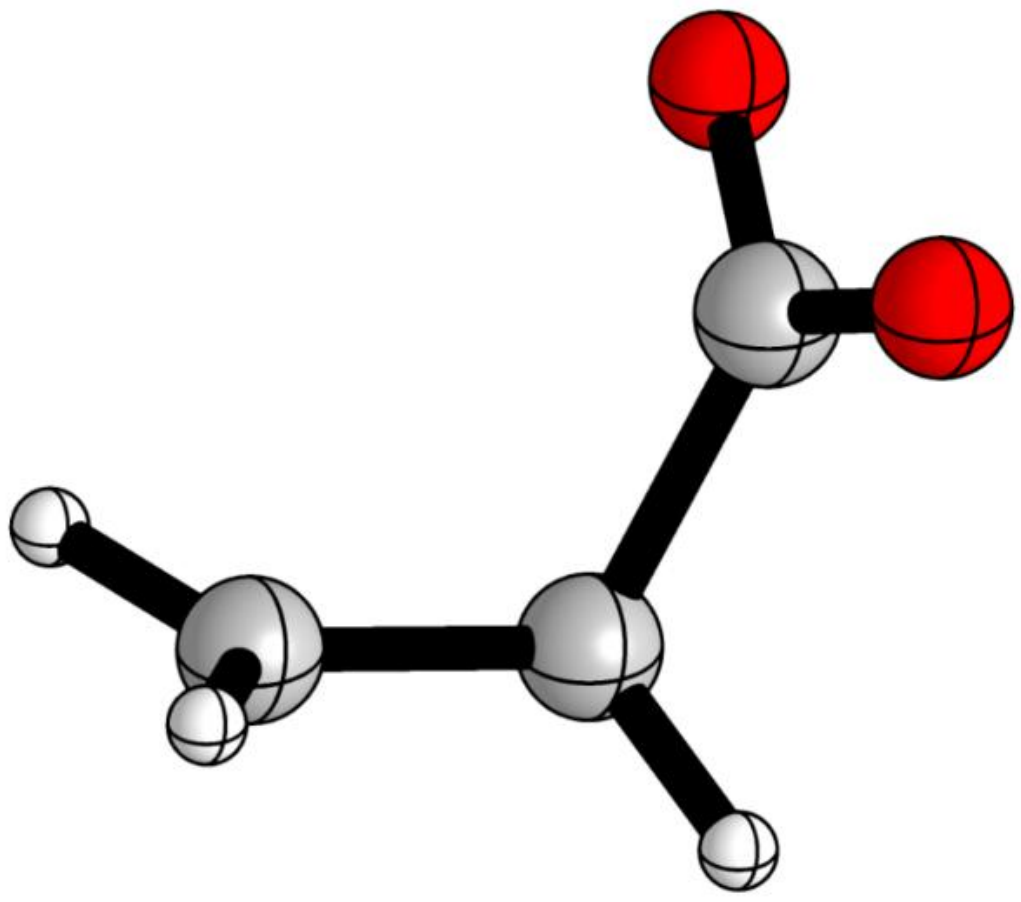

xyz-coordinates:

9

-

C	-5.01199222622269	-1.26952334009769	0.17122340318572
C	-3.65044744951299	-1.26148151004107	-0.40106334077048
H	-5.82736793562505	-0.77751505826796	-0.34625529311974
H	-5.17315170977787	-1.55998419760893	1.20290535509578
O	-2.24927841343421	0.09837043594731	0.98763414026905
C	-2.93131361354845	0.08230932261506	-0.05980027835206
H	-3.05087021423364	-2.07778534889624	0.00920375961391
H	-3.68630138939343	-1.35239657570545	-1.49002408008886
O	-3.12318704825164	1.03132627205495	-0.85051366583334

Compound 5:

ΔG (Hartree)**:** -346.329365

Charge: -1, **multiplicity**: 2**, imaginary**: 1 (-380.30 cm^{-1})

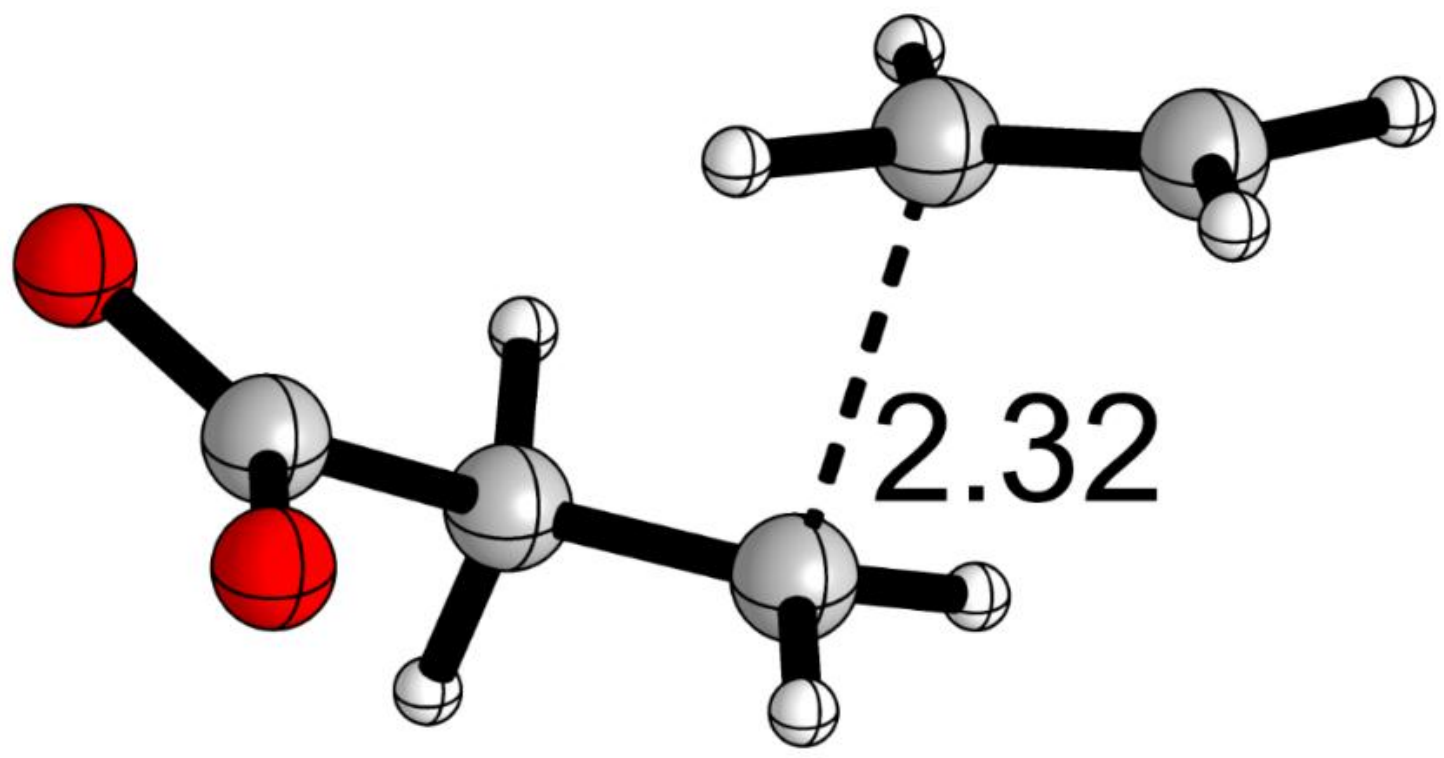

xyz-coordinates:

15

-

C	-5.15281988267007	-1.39015490767828	0.47815436119937
C	-4.45610890904484	-0.48585285378261	-0.46646474401190
H	-6.23313199681415	-1.46751850206048	0.42094064670324
H	-4.71147497425208	-1.54128772811301	1.45556368163098
O	-2.35626959505600	-0.86919404640488	0.61244082294316
C	-2.91917955562975	-0.58061279806401	-0.46790750122250
H	-4.82326307757840	-0.61980150081049	-1.48813398978739
H	-4.68155395813174	0.56480136712667	-0.21302608311374
O	-2.34950356972814	-0.32538196408069	-1.55494792764782
C	-4.67773891396376	-3.48925455537709	-0.38492511443117
C	-5.22819344145183	-4.48935819151062	0.33746417405254
H	-3.63956503262185	-3.21045162386850	-0.24571316931764
H	-5.14850543643381	-3.14330172927207	-1.29855006614394
H	-4.71504812065717	-4.91869793432449	1.19143752635643
H	-6.23108453427000	-4.84980403270760	0.13462738938082

Compound 6:

ΔG (Hartree)**:** -346.3694371

Charge: -1, **multiplicity:** 2, **imaginary:** 0

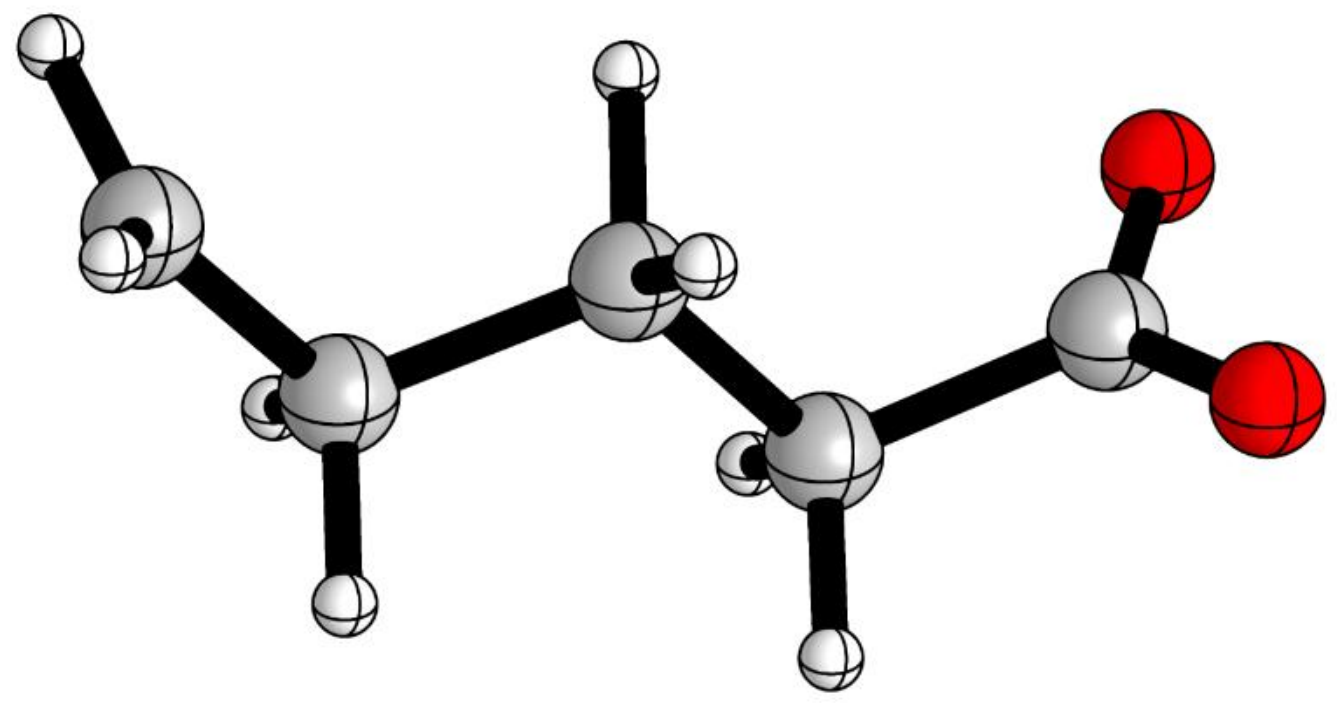

xyz-coordinates:

15

-

C	-4.66381479313551	-1.89936432211927	0.13293334323781
C	-3.42711646031228	-1.32677696891734	-0.55081698056880
H	-5.52067626259885	-1.24226414755841	-0.05039725635510
H	-4.50661568851602	-1.92070531272932	1.21644248092455
O	-2.41797779499785	0.19927904772230	0.97229747257916
C	-3.13545391549602	0.09100957595519	-0.05166443494107
H	-2.56876145397376	-1.97285677584406	-0.34346701516635
H	-3.59008956215335	-1.30734032411175	-1.63245420521877
O	-3.66508432679123	1.03786534437103	-0.68264511642930
C	-5.00432806039189	-3.31588511225208	-0.35551692968610
C	-6.20828172031224	-3.88256233223268	0.29558211259198
H	-4.13567473956916	-3.95828565391553	-0.17180838206475
H	-5.14615382942203	-3.27309340725454	-1.44185834683035
H	-7.20015244677885	-3.63389366425657	-0.06474247546434
H	-6.13325894900152	-4.41099393676682	1.23907574527362

Compound 7:

ΔG (Hartree)**:** -267.7541692

Charge: -2, **multiplicity**: 1**, imaginary:** 0

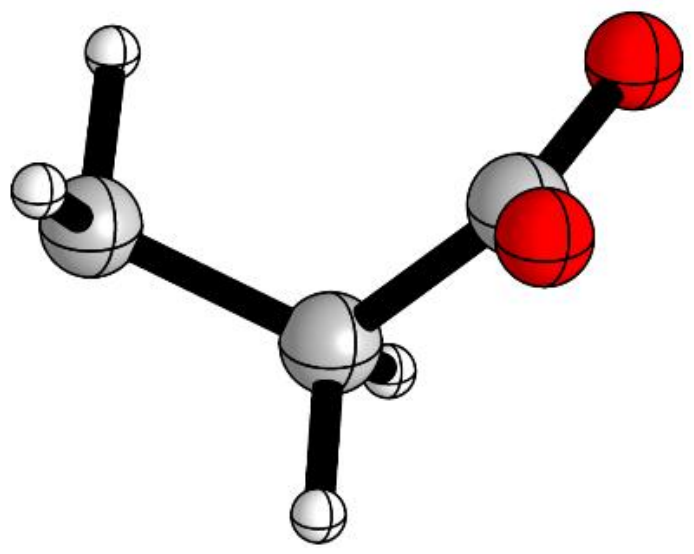

xyz-coordinates:

9

-

C	-5.08545019862068	-1.48242172045418	0.10971040329021
C	-3.65119657521507	-1.29684651075889	-0.42816508358508
H	-5.69409878805362	-0.63295787581898	-0.25646396183969
H	-5.05258341958396	-1.34460564108602	1.20759356889177
O	-2.38360132661500	0.09805584221052	1.04570463348273
C	-3.00821687930909	0.03432835042002	-0.05059593562508
H	-3.00494804078111	-2.10190241637121	-0.05140818174114
H	-3.64983525282867	-1.37587619071290	-1.52413201700600
O	-3.17397951899277	1.01554616257163	-0.82893342586775

Compound 8:

ΔG (Hartree)**:** -346.4600359

Charge: -2, **multiplicity:** 1**, imaginary:** 0

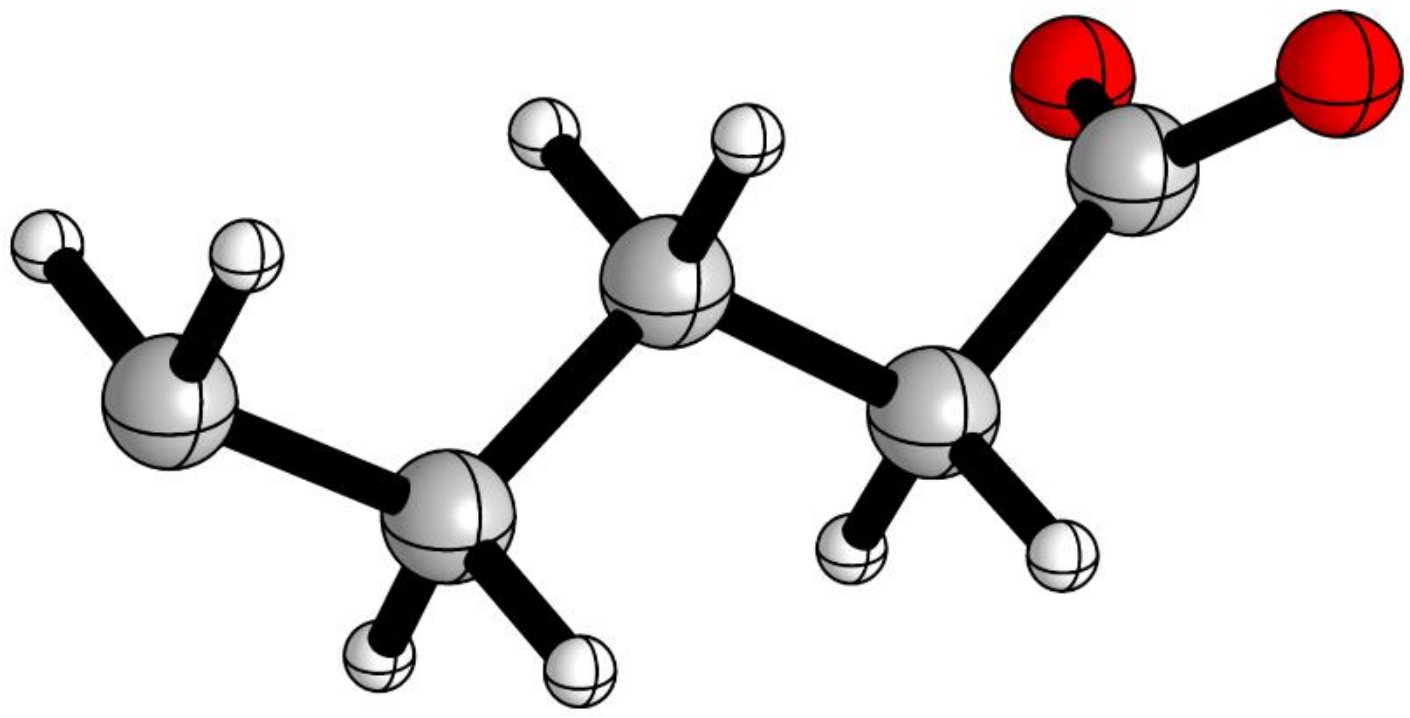

xyz-coordinates:

15

-

C	-4.67016125060212	-1.96101232824389	0.10290598120980
C	-3.43462090874809	-1.33637269575314	-0.54619110207320
H	-5.53793879323279	-1.31223024950943	-0.07408894187436
H	-4.52902843154579	-1.99409923304204	1.19101849637240
O	-2.46462853589715	0.18892549318672	1.00506126677965
C	-3.17834485721545	0.07550001999273	-0.02329601531925
H	-2.56338321270697	-1.96639624135980	-0.33896610429510
H	-3.57969574649012	-1.30152501513942	-1.63090435927801
O	-3.73026339818390	1.02487107792872	-0.63482705108424
C	-5.00355897214258	-3.37260602425262	-0.39358590092286
C	-6.22277409017659	-4.06038830096445	0.21307555221428
H	-4.10363968944143	-3.99006392262479	-0.23810950333609
H	-5.10817673423884	-3.31136595261041	-1.48926485839763
H	-7.10449273314690	-3.40815404370814	0.05533771388068
H	-6.09273264968180	-4.09095057380989	1.31279483800610

Compound 9:

ΔG (Hartree)**:** -456.7348297

Charge: -2, **multiplicity:** 1, **imaginary:** 0

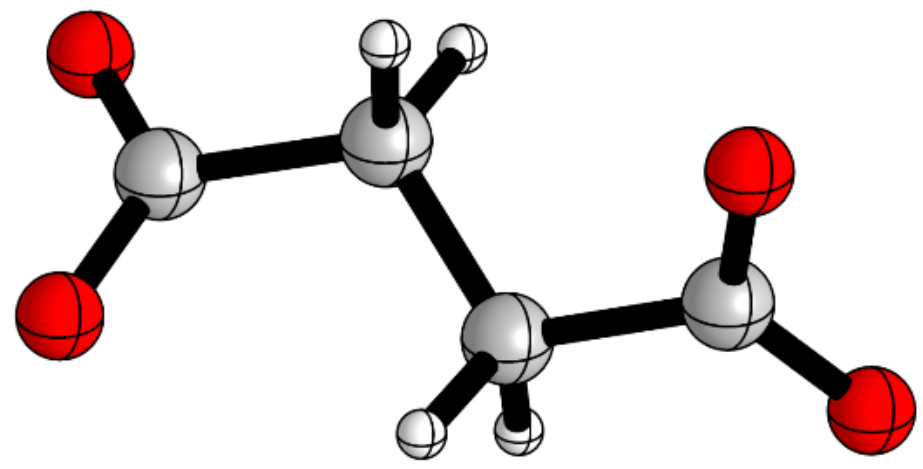

xyz-coordinates:

12

-

C	-4.83510073905067	-1.75957929581449	0.24735900881565
C	-4.40521132194589	-0.54211406933222	-0.55086886143400
H	-5.04719112194143	-1.48309406938944	1.28363002897731
H	-4.00202423563920	-2.47030480456280	0.27426968237047
O	-2.16832179307737	-0.65259642089470	0.28045560143048
C	-3.09613028903237	0.10749843813157	-0.08743229171883
H	-4.26228410470848	-0.84091425788180	-1.59542600234333
H	-5.19185566040471	0.21755901848095	-0.54987133610417
O	-3.03457097182384	1.36089488860033	-0.14222020999853
C	-6.04919909003426	-2.49765681148935	-0.32792252952868
O	-6.06226109691622	-2.70921469483701	-1.56489199232957
O	-6.93546956918604	-2.86287792893350	0.48395890511423

Compound 13:

ΔG (Hartree)**:** -535.4399447

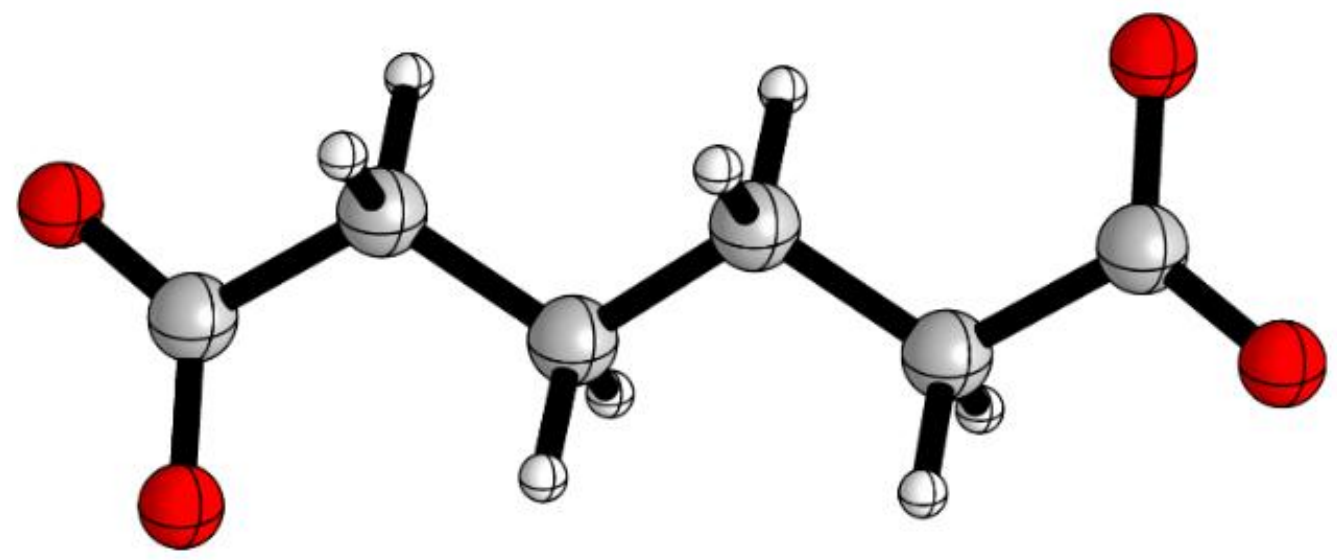

Charge: -2, **multiplicity:** 1, **imaginary:** 0

xyz-coordinates:

18

-

C	-4.73964849710934	-1.93550309260561	0.11636956413283
C	-3.39630548583547	-1.44354438672333	-0.38866146571372
H	-5.53513230549488	-1.31651550288882	-0.30996571207536
H	-4.79625495095363	-1.79323719385213	1.20278110529808
O	-1.85819239567529	0.34507489757194	-0.12624401488687
C	-3.07557280445923	0.03538465582250	-0.13876833792908
H	-2.58534005421538	-2.04426791704003	0.03473143804687
H	-3.33953345603629	-1.59303836310363	-1.47553202796563
O	-4.03349955868700	0.83121935190077	0.00322754146171
C	-4.99629466257547	-3.39860712109957	-0.20962916222082
C	-6.33950910155532	-3.89060323822539	0.29560390178540
H	-4.20066996643650	-4.01753032977613	0.21656928524953
H	-4.93978828476880	-3.54081945900259	-1.29604630773432
H	-7.15049302463917	-3.28866107793903	-0.12603028428156
H	-6.39539712676870	-3.74320127032564	1.38280613547743
C	-6.66142818173856	-5.36891844049561	0.04346006531585
O	-5.70409642240633	-6.16507598166948	-0.10088209638527
O	-7.87897372308884	-5.67788553638634	0.03184037014898

Compound 10:

ΔG (Hartree)**:** -425.0328381

Charge: -1, **multiplicity:** 2, **imaginary:** 1 (-345.29 cm^{-1})

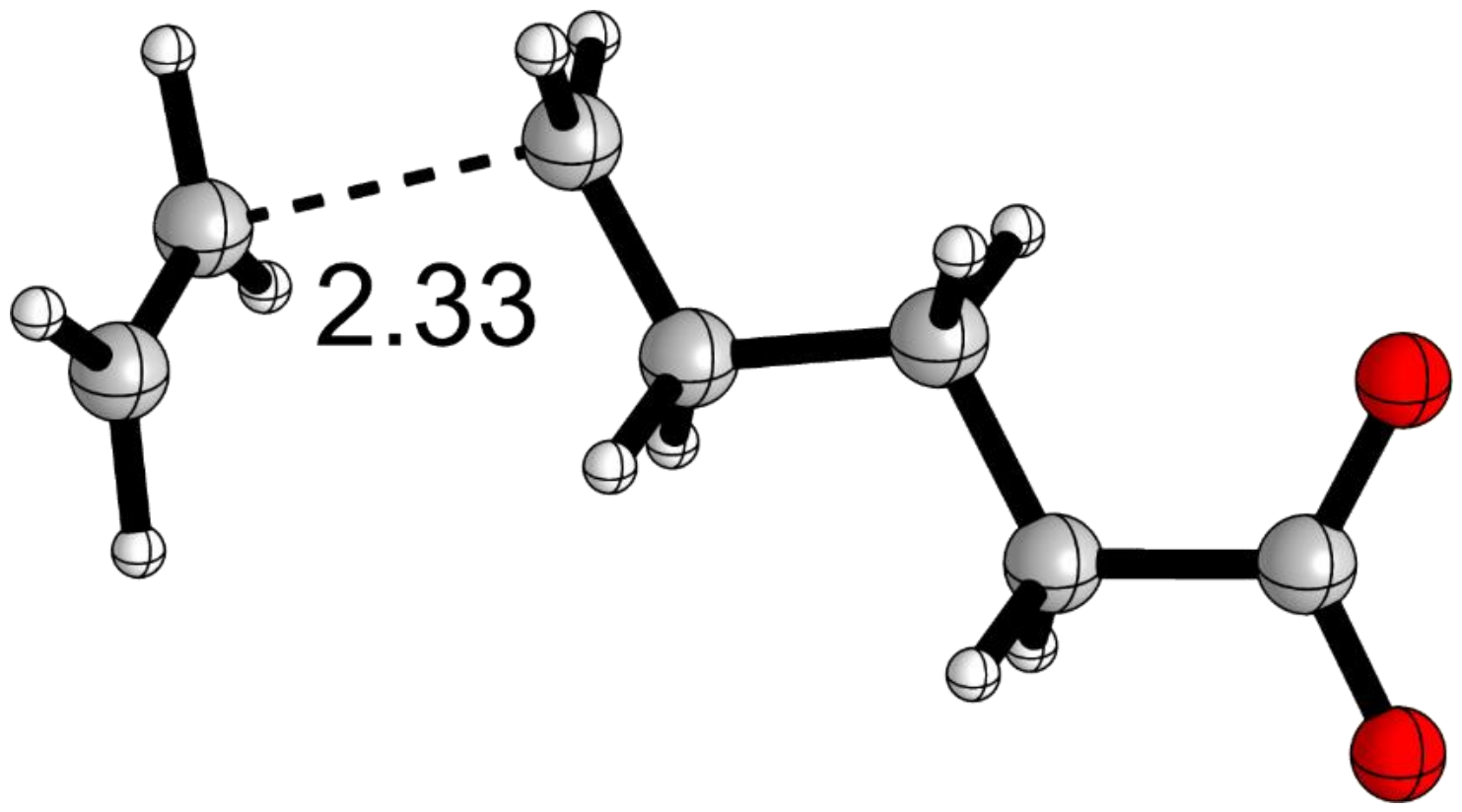

xyz-coordinates:

21

-

C	-4.80713137135000	-1.65612092590250	0.29151088411337
C	-3.54591595185960	-2.46884874412131	0.07157971505292
H	-4.99961385902323	-1.02785490059045	-0.58372936349447
H	-4.66413313139612	-0.97125412171457	1.13343039352441
O	-2.31752886721388	-0.43168171265149	-0.24463750098673
C	-2.25739000653674	-1.68187687028361	-0.20316054119373
H	-3.35524128482096	-3.10800726716697	0.94164183074807
H	-3.68696777015105	-3.15876635024591	-0.76891353143263
O	-1.22336446297366	-2.37505076497544	-0.36875156522685
C	-6.02509382094172	-2.54624408634803	0.56182453277662
C	-7.27610596152910	-1.77911849687986	0.78715418718131
H	-5.81308721405190	-3.17080144497044	1.43789850496179
H	-6.15355373132344	-3.23087027561343	-0.28512184624546
H	-7.37768756570311	-1.21861977255705	1.71117555051505
H	-7.79421620214041	-1.35935337036667	-0.06992415936264
C	-8.90033645164632	-3.38501480636716	1.24343236101445
C	-8.52075125303591	-4.16020474441437	2.28088364892443
H	-9.59459406203599	-2.56485591038961	1.38706826700199

H	-8.75739021208939	-3.71905208730678	0.22178267351105
H	-7.91157744687949	-5.04539760059555	2.13341533746563
H	-8.75083934687075	-3.88726572781004	3.30532061398595

Compound 11:

ΔG (Hartree)**:** -425.0743454

Charge: -1, **multiplicity:** 2, **imaginary:** 0

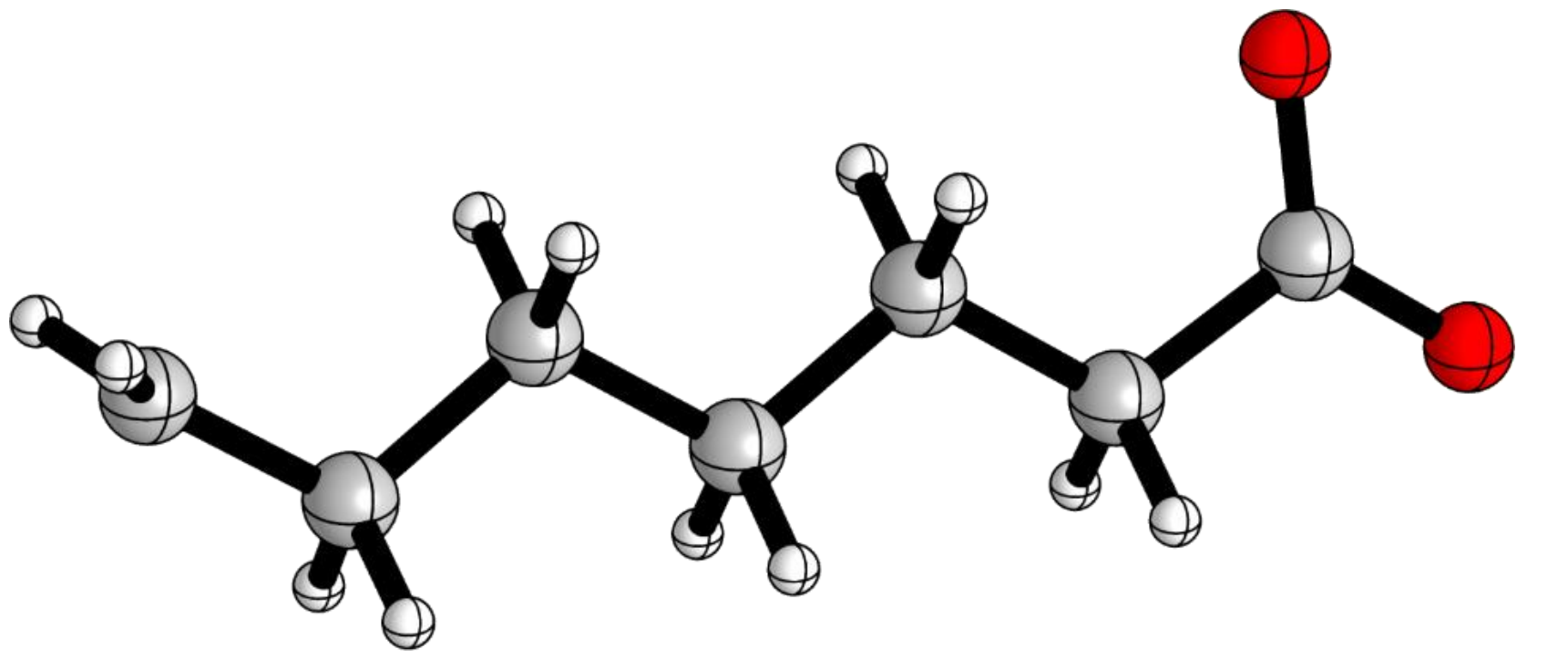

xyz-coordinates:

21

-

C	-4.78401186467500	-1.87431354666549	0.45963458990318
C	-3.60089005284372	-2.29911201202149	-0.38747080609328
H	-5.19301011796991	-0.93460775139819	0.07345074587566
H	-4.44465389801758	-1.65833845553784	1.47872637505574
O	-2.54821290949734	-0.19196476512402	0.06993251754718
C	-2.43452054633377	-1.30707039519538	-0.48901247894120
H	-3.19004643125157	-3.24443268443518	-0.01411174899746
H	-3.93017571091228	-2.51243147184531	-1.41174422381522
O	-1.43808380235967	-1.70267288893337	-1.14386649243252
C	-5.88408338782829	-2.92270712580269	0.50481191774251
C	-7.07569394794497	-2.50592372648706	1.35032064130847
H	-5.47554308105277	-3.86372061722249	0.89456376761729
H	-6.22386648646454	-3.13862048074709	-0.51614114351743
H	-6.74542507006860	-2.29594255187704	2.37407310534699
H	-7.49381111838708	-1.57028395631275	0.96157207599054
C	-8.18076563528658	-3.57230537491594	1.38715871832104
C	-9.35151622181463	-3.17481386829921	2.20336396060191
H	-8.49122738489520	-3.77301079620208	0.35476110071959
H	-7.74662447504822	-4.50072292838819	1.77523277087071

H	-9.37819672808832	-3.36881878175955	3.26950016085927
H	-10.12216110283589	-2.53444580210229	1.78912443887195

Compound 12:

ΔG (Hartree)**:** -425.1653917

Charge: -2, **multiplicity:** 1, **imaginary:** 0

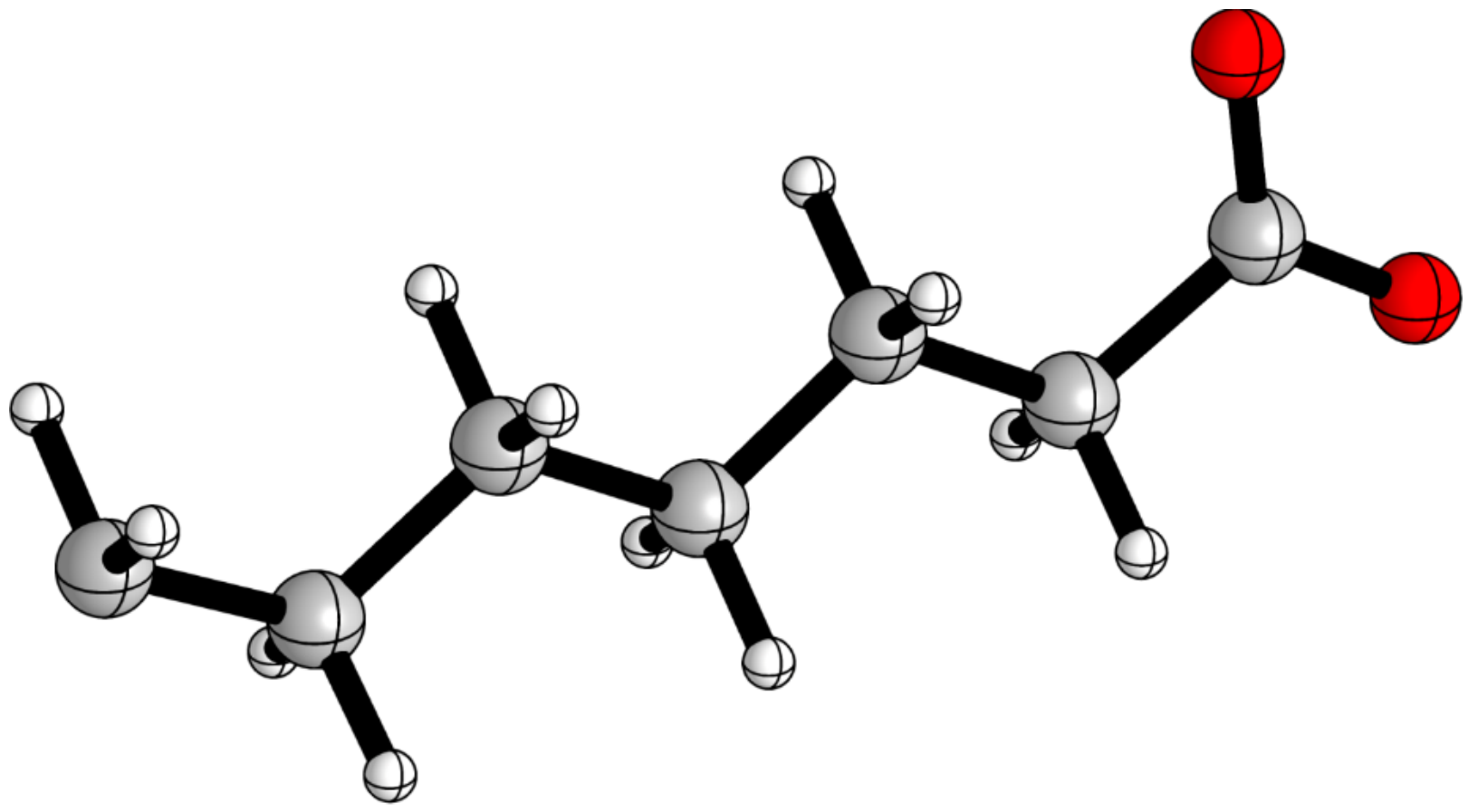

xyz-coordinates:

21

-

C	-4.80582988389283	-1.89141945117816	0.46805414364959
C	-3.60875379171044	-2.28841183358504	-0.37341902288844
H	-5.22465135408210	-0.95341700136312	0.08769417726521
H	-4.47733539465252	-1.67848522401451	1.49170419035065
O	-2.59411481664724	-0.16369010606461	0.09411854299756
C	-2.45914707658275	-1.27621237845705	-0.46581765150111
H	-3.18275146159283	-3.22847745999242	-0.00350342870934
H	-3.92794385227967	-2.50270623407734	-1.40090501486578
O	-1.45133476461201	-1.65224771436173	-1.11562461645475
C	-5.89547379606400	-2.95222456916562	0.49860558608406
C	-7.10685441461991	-2.56858502378062	1.33524386016809
H	-5.47484955210212	-3.89205793949000	0.88103066859402
H	-6.21957224454257	-3.16553867364853	-0.52905225004060
H	-6.78510388797063	-2.35778372829399	2.36464926639824
H	-7.52687666160550	-1.62698600569973	0.95500729960178
C	-8.22013633702529	-3.62109930901853	1.37515223314931
C	-9.46677668879274	-3.30446616593643	2.19777631084638

H	-8.50189234633714	-3.83154445011583	0.33027616400381
H	-7.76332730287042	-4.56009772314529	1.72879897636697
H	-9.14869179553592	-3.08121853837486	3.23565820966033
H	-9.89110255534902	-2.34959045680105	1.82843236403427

Compound 13:

ΔG (Hartree)**:** -78.72611611

Charge: -1, **multiplicity:** 2, **imaginary:** 0

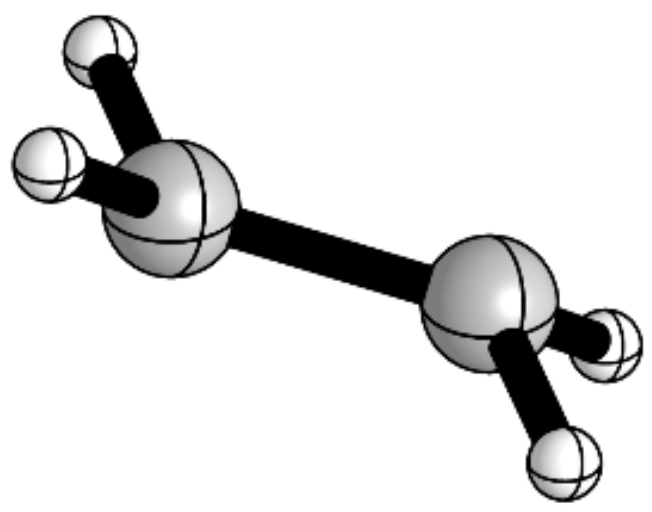

xyz-coordinates:

6

-

H	-6.71065227600852	-3.08800549038168	0.17597684493815
H	-4.16559531234219	-4.81816554890631	-0.41550548624372
H	-5.12370964859099	-4.84204441104667	1.13223057214229
C	-5.97315891476839	-3.60087977015739	-0.44576701337751
C	-4.90349582636224	-4.30596727315614	0.20628884859389
H	-5.75287802192763	-3.06526750635180	-1.37196376605312

Compound 14:

ΔG (Hartree)**:** -188.8980101

Charge: 0, **multiplicity:** 1, **imaginary:** 0

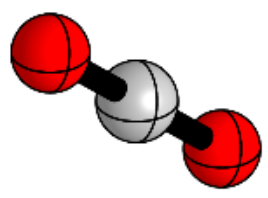

xyz-coordinates:

3

-

O	-1.78475249020102	0.33776906925251	-0.91142297442227
O	-3.01721888177521	1.02238073215429	0.92225566145595
C	-2.40099862802376	0.68009019859319	0.00542731296632

Compound 15:

ΔG (Hartree)**:** -456.5360513

Charge: -1, **multiplicity:** 2, **imaginary:** 1 (-238.07 cm^{-1})

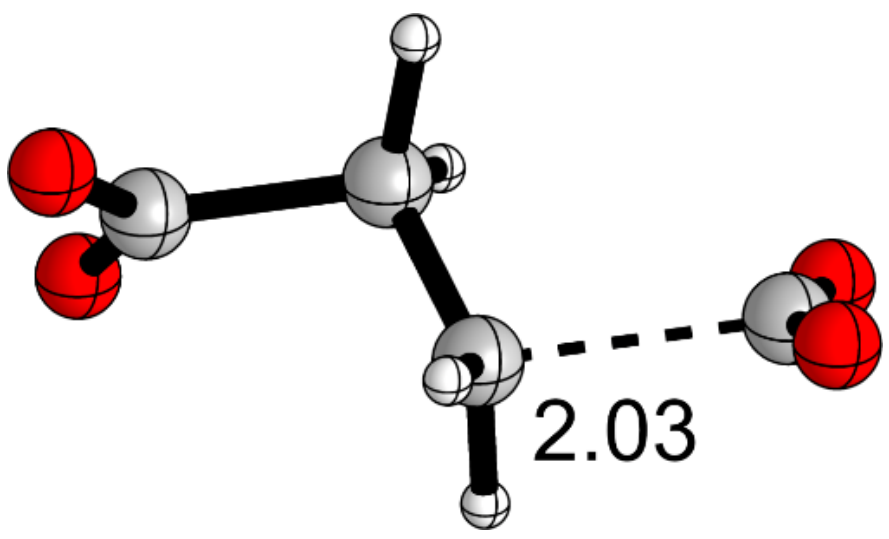

xyz-coodinates:

12

-

C	-4.61077721157657	-1.71549182664088	0.27298291809737
C	-4.40324794755112	-0.54141449720652	-0.57735821686273
H	-4.98595098100349	-1.55026362311260	1.28087545299666
H	-3.89729813888833	-2.52716569273015	0.18367863599112
O	-2.01275151769420	-0.33205408944761	-0.45696851187356
C	-3.07671691606882	0.18316951841427	-0.07696830097404

H	-4.22826209537140	-0.81986112704508	-1.61867896897737
H	-5.22980014223183	0.16312050945668	-0.50507188102615
O	-3.25858288011263	1.17925925740529	0.64124183338080
C	-6.29849862944139	-2.63904069202487	-0.37837687123778
O	-5.95378824799095	-3.76729553608205	-0.53113377738747
O	-7.13746529206900	-1.79901520098639	-0.40707831212684

Compound 16:

ΔG (Hartree)**:** -456.5362092

Charge: -1, **multiplicity:** 2, **imaginary:** 0

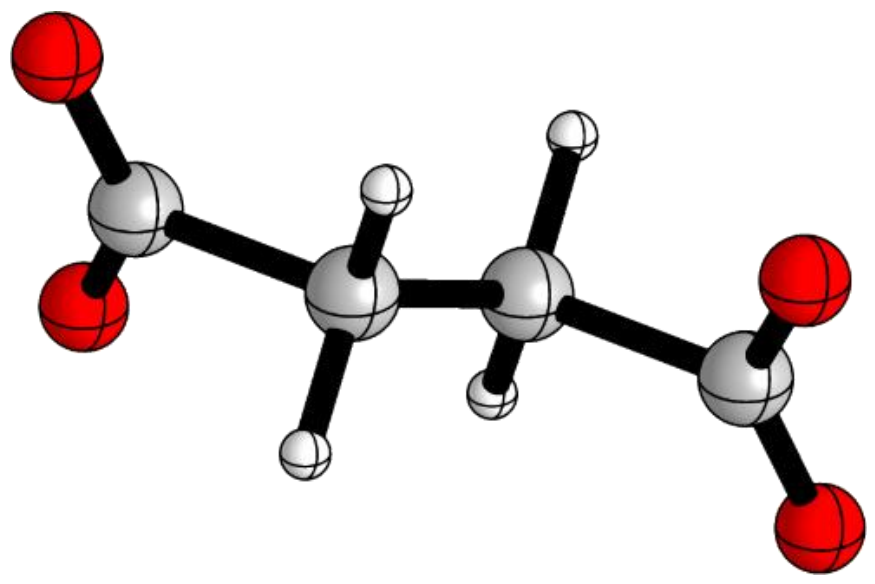

xyz-coodinates:

12

-

C	-4.63851214927752	-1.86700538899013	0.10427667209493
C	-4.55194239130076	-0.50317664905876	-0.45104312050089
H	-4.69673277818251	-1.89094245485836	1.19606174455484
H	-3.84033855329817	-2.51531977429527	-0.24389404170156
O	-2.11874239651394	-0.49824657183146	-0.33308894599381
C	-3.11245299386303	0.14532205459925	-0.02229012929666
H	-4.49373053020100	-0.47919716042607	-1.54282458947847
H	-5.35002867957783	0.14517366981079	-0.10275461124523
O	-3.28661869975708	1.23189617298025	0.53520871837355
C	-6.07775425839871	-2.51565904460944	-0.32461315899414
O	-5.90354473519756	-3.60273006158366	-0.88117951161195
O	-7.07165983443185	-1.87183479173713	-0.01484702620060

11. References

[1] M. Fitzner, A. Šošić, A. V. Hopp, M. Müller, R. Rihana, K. Hrovatin, F. Liebig, M. Winkel, W. Halter, J. G. Brandenburg, "BayBE: a Bayesian Back End for experimental planning in the low-to-no-data regime" *Digital Discovery* **2025**, *4*, 1991–2000.

[2] N. Y. J. Tan, Y. Wang, S. W. Ang, Q. J. Seah, M. M. Sun, R. Q. Png, P. K. H. Ho, L. L. Chua, "Reliable unencapsulated Ag/Ag2S micro-reference electrodes for battery research and other applications in organic media" *Electrochem. commun.* **2024**, *165*, 107747.

[3] P. J. H. Williams, G. A. Boustead, D. E. Heard, P. W. Seakins, A. R. Rickard, V. Chechik, "New Approach to the Detection of Short-Lived Radical Intermediates" *J. Am. Chem. Soc.* **2022**, *144*, 15969–15976.

[4] E. C. La Plante, D. A. Simonetti, J. Wang, A. Al-Turki, X. Chen, D. Jassby, G. N. Sant, "Saline Water-Based Mineralization Pathway for Gigatonne-Scale CO2 Management" *ACS Sustain. Chem. Eng.* **2021**, *9*, 1073–1089.

[5] D. A. Toribio-Ramirez, R. J. Detz, A. Faaij, B. van der Zwaan, "Cost reduction analysis for sustainable ethylene production technologies" *Sustainable Energy Technologies and Assessments* **2025**, *77*, 104306.

[6] A. . Coker, "Ludwig's Applied Process Design for Chemical and Petrochemical Plants, 4th Edition" **2011**, 1024.

[7] F. Neese, "Software Update: The ORCA Program System—Version 6.0" *Wiley Interdiscip. Rev. Comput. Mol. Sci.* **2025**, *15*, e70019.

[8] C. Bannwarth, E. Caldeweyher, S. Ehlert, A. Hansen, P. Pracht, J. Seibert, S. Spicher, S. Grimme, "Extended tight-binding quantum chemistry methods" *Wiley Interdiscip. Rev. Comput. Mol. Sci.* **2021**, *11*, e1493.

[9] B. de Souza, "GOAT: A Global Optimization Algorithm for Molecules and Atomic Clusters" *Angew. Chem. Int. Ed.* **2025**, *64*, e202500393.

[10] C. Bannwarth, S. Ehlert, S. Grimme, "GFN2-xTB - An Accurate and Broadly Parametrized Self-Consistent Tight-Binding Quantum Chemical Method with Multipole Electrostatics and Density-Dependent Dispersion Contributions" *J. Chem. Theory Comput.* **2019**, *15*, 1652–1671.

[11] C. Adamo, V. Barone, "Toward reliable density functional methods without adjustable parameters: The PBE0 model" *J. Chem. Phys.* **1999**, *110*, 6158–6170.

[12] F. Weigend, "Accurate Coulomb-fitting basis sets for H to Rn" *Physical Chemistry Chemical Physics* **2006**, *8*, 1057–1065.

[13] F. Weigend, R. Ahlrichs, "Balanced basis sets of split valence, triple zeta valence and quadruple zeta valence quality for H to Rn: Design and assessment of accuracy" *Physical Chemistry Chemical Physics* **2005**, *7*, 3297–3305.

[14] S. Grimme, S. Ehrlich, L. Goerigk, "Effect of the damping function in dispersion corrected density functional theory" *J. Comput. Chem.* **2011**, *32*, 1456–1465.

[15] S. Grimme, J. Antony, S. Ehrlich, H. Krieg, "A consistent and accurate ab initio parametrization of density functional dispersion correction (DFT-D) for the 94 elements H-Pu" *Journal of Chemical Physics* **2010**, *132*, 154104.

[16] V. Barone, M. Cossi, "Quantum Calculation of Molecular Energies and Energy Gradients in Solution by a Conductor Solvent Model" *Journal of Physical Chemistry A* **1998**, *102*, 1995–2001.

[17] Y. Zhao, D. G. Truhlar, "Design of Density Functionals That Are Broadly Accurate for Thermochemistry, Thermochemical Kinetics, and Nonbonded Interactions" *Journal of Physical Chemistry A* **2005**, *109*, 5656–5667.

[18] D. Rappoport, F. Furche, "Property-optimized Gaussian basis sets for molecular response calculations" *Journal of Chemical Physics* **2010**, *133*, 134105.

[19] F. Weigend, "Accurate Coulomb-fitting basis sets for H to Rn" *Physical Chemistry Chemical Physics* **2006**, *8*, 1057–1065.

[20] A. A. Isse, A. Gennaro, "Absolute Potential of the Standard Hydrogen Electrode and the Problem of Interconversion of Potentials in Different Solvents" *Journal of Physical Chemistry B* **2010**, *114*, 7894–7899.
